# Differentiation and Transplantation of Embryonic Stem Cell-Derived Cone Photoreceptors into a Mouse Model of End-Stage Retinal Degeneration

**DOI:** 10.1016/j.stemcr.2017.04.030

**Published:** 2017-05-25

**Authors:** Kamil Kruczek, Anai Gonzalez-Cordero, Debbie Goh, Arifa Naeem, Mindaugas Jonikas, Samuel J.I. Blackford, Magdalena Kloc, Yanai Duran, Anastasios Georgiadis, Robert D. Sampson, Ryea N. Maswood, Alexander J. Smith, Sarah Decembrini, Yvan Arsenijevic, Jane C. Sowden, Rachael A. Pearson, Emma L. West, Robin R. Ali

**Affiliations:** 1 Department of Genetics, UCL Institute of Ophthalmology, London EC1V 9EL, UK; 2 Department of Ophthalmology, Jules-Gonin Eye Hospital, University of Lausanne, 1004 Lausanne, Switzerland; 3 Stem Cells and Regenerative Medicine Section, UCL Great Ormond Street Institute of Child Health, University College London, London WC1N 1EH, UK; 4 NIHR Biomedical Research Centre, Moorfields Eye Hospital NHS Foundation Trust, City Road, London EC1V 2PD, UK

**Keywords:** blindness, cell- and tissue-based therapy, mouse embryonic stem cells, retina, retinal cone photoreceptor cells, retinal degeneration

## Abstract

The loss of cone photoreceptors that mediate daylight vision represents a leading cause of blindness, for which cell replacement by transplantation offers a promising treatment strategy. Here, we characterize cone differentiation in retinas derived from mouse embryonic stem cells (mESCs). Similar to in vivo development, a temporal pattern of progenitor marker expression is followed by the differentiation of early thyroid hormone receptor β2-positive precursors and, subsequently, photoreceptors exhibiting cone-specific phototransduction-related proteins. We establish that stage-specific inhibition of the Notch pathway increases cone cell differentiation, while retinoic acid signaling regulates cone maturation, comparable with their actions in vivo. MESC-derived cones can be isolated in large numbers and transplanted into adult mouse eyes, showing capacity to survive and mature in the subretinal space of *Aipl1*^*−*/*−*^ mice, a model of end-stage retinal degeneration. Together, this work identifies a robust, renewable cell source for cone replacement by purified cell suspension transplantation.

## Highlights

• Cone photoreceptor precursors form efficiently in mESC retinal organoids
• Notch signaling limits temporal competence for cone cell differentiation
• Reduced retinoic acid concentrations are required for cone maturation
• Cones transplanted into a model of advanced retinal degeneration survive and mature

## Graphical Abstract

**Figure:**
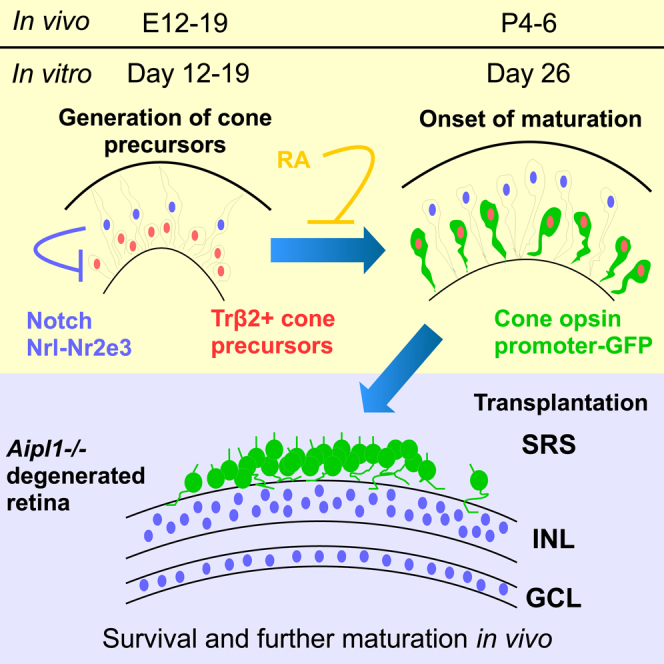

## Teaser

Ali and colleagues characterize the differentiation of cone photoreceptors in mESC retinal organoids, showing substantial numbers of committed cone precursors. Their differentiation is negatively regulated by the Notch pathway, expression of Nrl and Nr2e3, and high concentrations of retinoic acid. Isolated and transplanted into a severely degenerated retina, these cone precursors survive and express markers of advanced maturation.

## Introduction

Cone photoreceptors mediate high acuity and color vision in daylight. Cones are a rare subtype in most mammals, constituting approximately 3% of photoreceptors in mice and 5% in humans (Jeon et al., 1998). Due to scarcity, our understanding of their development and physiology is less advanced than that of rods, the major photoreceptor type responsible for night vision (Swaroop et al., 2010). A common feature of both inherited and age-related retinal degeneration is the death of photoreceptors. Loss of cones has a far greater effect on vision in patients, and replacement of small numbers of cones may result in substantial benefits (Jayakody et al., 2015). Interest in developing methods of photoreceptor differentiation from stem cells is driven by the potential to establish cell-based therapies to treat retinal degenerations (Jayakody et al., 2015), as well as providing in vitro models both to study disease mechanisms and for drug discovery. Two main approaches for photoreceptor replacement have been developed in pre-clinical research. In the first, a whole neural retina sheet is placed into the diseased environment (Seiler and Aramant, 2012). The second is transplantation of cells in suspension (Jayakody et al., 2015). Retinal sheet transplantation has the advantage of supplying a structured tissue, although injection of a flat, thick sheet of cells with the correct polarity into the subretinal space is surgically challenging (Reh, 2016, Seiler and Aramant, 2012). Moreover, inclusion of interneurons limits the connectivity of graft photoreceptors to host inner retinal circuitry (Assawachananont et al., 2014, Reh, 2016, Shirai et al., 2016). By contrast, dissociated photoreceptors can be purified to remove other cell types that may prevent them from forming synaptic contacts with the host interneurons (Singh et al., 2013). Postmitotic photoreceptor precursors isolated from donor mice injected subretinally as a suspension are capable of driving visual function in models of retinal degeneration (Barnea-Cramer et al., 2016, MacLaren et al., 2006, Pearson et al., 2012, Santos-Ferreira et al., 2015, Singh et al., 2013).

Despite the importance of cones for human vision, few studies have looked at the possibility of transplanting isolated donor-derived cones (Lakowski et al., 2010, Santos-Ferreira et al., 2015, Smiley et al., 2016). We have reported transplantation of a mixed population of embryonic cone rod homeobox-positive (CRX^+^) photoreceptors, enriched for cones compared with postnatal stages (Lakowski et al., 2010), while Ader and colleagues used cone-like cells from mice lacking NRL (neural retina leucine), a transcription factor essential for rod genesis, reporting an improvement in light-evoked responses in mice lacking cone function (Santos-Ferreira et al., 2015). In a recent proof-of-concept study, cones purified using a novel reporter mouse line were transplanted into wild-type retina (Smiley et al., 2016). However, the cones observed in the host photoreceptor layer resembled rods morphologically and have subsequently been shown to result from uptake of cytoplasmic components by host photoreceptors from the graft (Decembrini et al., 2017, Ortin-Martinez et al., 2016, Pearson et al., 2016). Therefore, the true morphology and maturation characteristics of transplanted cones remain largely unknown. Notably, none of the previous studies examined transplantation into a model exhibiting extensive photoreceptor loss, a feature observed in patients with advanced retinal disease that would benefit most from photoreceptor transplantation (Reh, 2016).

Another limitation of these earlier studies is the use of primary donor-derived cells, while successful clinical translation of these findings will require a renewable, efficient, and scalable cell source. Advances in retinal differentiation using mouse and human pluripotent stem cells (PSCs) (Eiraku et al., 2011, Gonzalez-Cordero et al., 2013, Meyer et al., 2009, Nakano et al., 2012, Zhong et al., 2014), including the generation of optic cups in 3D floating culture of mESCs, enabled the transplantation of ESC- and induced PSC-derived retinal sheets (Assawachananont et al., 2014, Shirai et al., 2016) as well as suspensions of purified rod precursors (Decembrini et al., 2014, Gonzalez-Cordero et al., 2013). However, cone differentiation in these cultures has not been characterized, nor has their transplantation potential been assessed.

In early stages of commitment to the cone lineage, co-expression of transcription factors ONECUT1 and OTX2 in retinal progenitors positive for OLIG2 leads to transcriptional activation of thyroid hormone receptor β2 (TRβ2) (Emerson et al., 2013), an early cone marker (Ng et al., 2009). An important negative regulator of cone and rod genesis is the Notch pathway, acting to promote non-photoreceptor fates (Mizeracka et al., 2013). Little is known about the soluble signals cooperating with intrinsic determinants to regulate cone differentiation. In zebrafish, application of exogenous retinoic acid (RA) results in precocious rod differentiation, while the accompanying cones retain immature morphology (Hyatt et al., 1996). Similarly, RA facilitates rod gene expression in mESC-derived retinal organoids (Gonzalez-Cordero et al., 2013). Nevertheless, its effects on mammalian cone differentiation remain undetermined.

In this work, we characterize formation of mESC-derived cones and establish that in vitro the Notch pathway and RA signaling regulate their differentiation and maturation, respectively. Furthermore, we demonstrate that mESC-derived retinas generate a significant proportion of cone precursors. This permitted the isolation of large numbers of purified cones for transplantation into the *Aipl1*^*−*/*−*^ mouse model of end-stage degeneration, in which nearly all host photoreceptors are lost by postnatal day 30 (P30) (Ramamurthy et al., 2004). In this environment, transplanted mESC-derived cone photoreceptors show survival and maturation features that cannot result from cytoplasmic material transfer. Together, we provide a proof of concept for cone cell replacement via purified cell suspension transplantation.

## Results

### Recapitulation of Stepwise Commitment to the Cone Lineage in mESC-Derived Retinas

**Figure 1:**
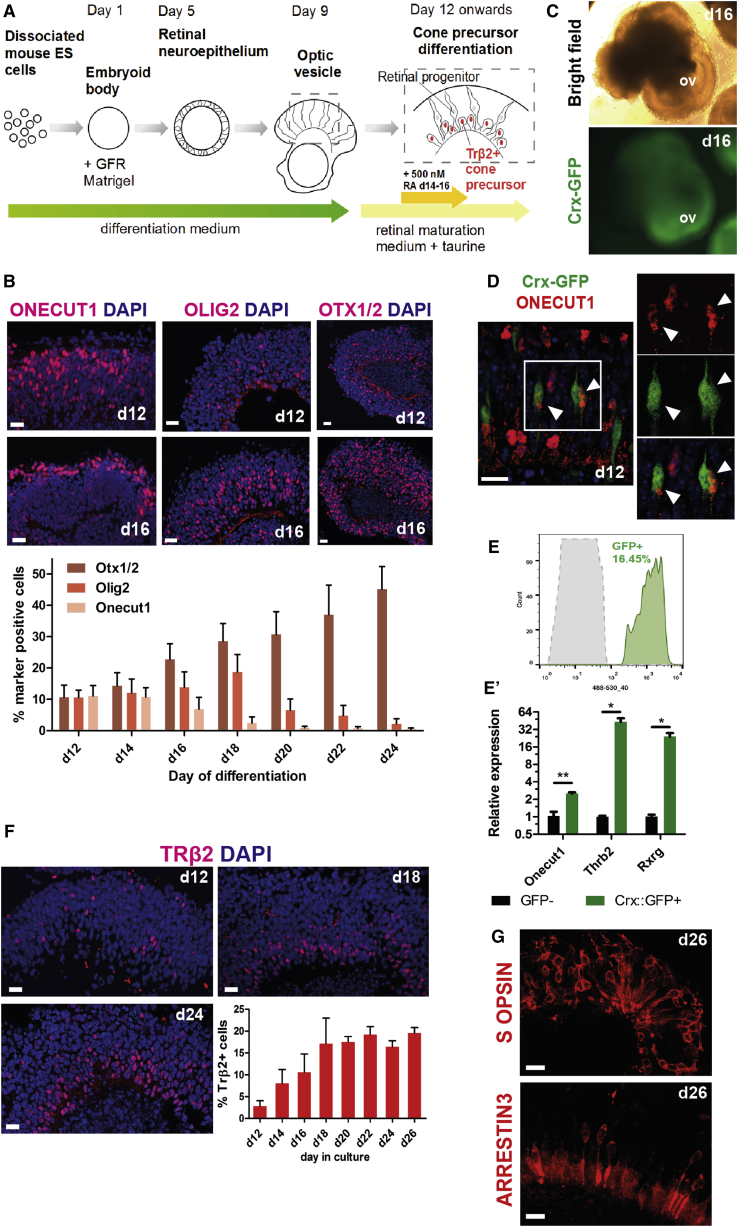
Sequential Commitment to the Cone Photoreceptor Lineage Is Recapitulated In Vitro in mESC-Derived Retinas (A) Schematic depiction of the differentiation protocol used in the study. (B) Expression of ONECUT1, OLIG2, and OTX2 determined by immunostaining. d, day. Scale bar, 20 μm. Quantification: for each time point n > 10 images of neural retinal regions from different organoids, N = 3 differentiation cultures. Mean ± SD. (C) Crx-GFP retinal organoids showing expression of the fluorescent reporter. ov, optic vesicle. (D) Co-staining of Crx-GFP^+^ photoreceptor precursors with ONECUT1 (arrowheads). Scale bar, 10 μm. (E) Flow-cytometry histogram showing GFP reporter expression in dissociated Crx-GFP line aggregates at day 16 of differentiation. (E′) qPCR analysis of *Onecut1*, *Thrb2*, and *Rxrg* expression in flow-sorted Crx-GFP^+^ versus GFP^−^ populations. N = 3, Mean ± SD. ^∗^p < 0.05, ^∗∗^p < 0.01, Student's t test. (F) Immunostaining for TRβ2 in mESC retinal organoids at days 12, 18, and 24 of differentiation. Quantification showing percentage of positive nuclei at indicated time points. n > 10 neural retina regions in individual organoids from N = 3 differentiation cultures quantified for each time point. Mean ± SD. Scale bar, 20 μm. (G) Antibody staining for S OPSIN and ARRESTIN3 at day 26 in culture. Scale bar, 10 μm.

To examine cone differentiation from mESCs, we adapted an established protocol for the generation of retinal organoids recapitulating early retinal histogenesis (Figures 1A and S1A–S1D; Decembrini et al., 2014, Eiraku et al., 2011, Gonzalez-Cordero et al., 2013). In vivo, a subpopulation of retinal progenitors biased toward cone genesis is marked by co-expression of the transcription factors ONECUT1, OTX2, and OLIG2 (Emerson et al., 2013, Hafler et al., 2012). Cone genesis is completed before birth in murine retina (Carter-Dawson and LaVail, 1979). On day 12 (d12) to d18 in culture, which corresponds to between embryonic day 12 (E12) and E18 in vivo (see Figure 4E for comparison with in vivo development [Decembrini et al., 2014, Eiraku et al., 2011, Gonzalez-Cordero et al., 2013, Swaroop et al., 2010]), gene expression analysis (Figure S1E) and immunohistochemistry (Figure 1B) showed expression of ONECUT1, OTX2, and OLIG2 in retinal organoids. Quantification of the number of cells expressing these proteins in the neural retina-like regions of the organoids revealed a dynamic temporal pattern. The percentage of ONECUT1^+^ cone and horizontal cell progenitors decreased markedly between equivalent of embryonic (d12, 11% ± 3%) and neonatal (d20, 1% ± 0.5%) stages (n > 10 images of individual organoids for each time point; N = 3 differentiation cultures). Conversely, the percentage of OTX2^+^ cells, which marks all photoreceptor precursors together with bipolar cells (Nishida et al., 2003), continued to rise (11% ± 4% on d12 versus 31% ± 7% on d20). OLIG2 was most widely expressed at d18 (19% ± 6%), correlating with the peak of rod birth in these cultures (Eiraku et al., 2011) and consistent with its expression in progenitors giving rise to both rods and cones (Hafler et al., 2012). Since ONECUT1 is initially expressed both in cones and horizontal cells, we sought to examine its expression in the early photoreceptor precursor population. We utilized organoids derived from the previously characterized Crx-GFP reporter mESC line (Decembrini et al., 2014; Figure 1C), in which GFP localizes to developing photoreceptor precursors. Immunostaining for ONECUT1 only showed co-localization in a small subpopulation of Crx-GFP^+^ cells at d12 of differentiation (Figure 1D) and was no longer detectable at d20 (Figure S1F), consistent with its transient expression in developing cones in vivo (Emerson et al., 2013). As predicted, in the neural retina which constitutes most of the organoid tissue, OTX2 staining overlapped significantly with the GFP signal (shown at d24 in Figure S1G). Together, these observations suggest that the temporal appearance of markers of progenitor competence for cone genesis is largely recapitulated in vitro.

In early postmitotic cone precursors, OTX2 and ONECUT1 bind to regulatory sequences in the *Thrb2* gene locus to stimulate its transcription (Emerson et al., 2013). These cells are also characterized by expression of retinoid receptor gene *Rxrg* (Roberts et al., 2005). To compare gene expression in photoreceptors from retinal organoids with previously reported data for the parental Crx-GFP transgenic mouse line at E16 (Muranishi et al., 2010), we purified GFP^+^ cells at d16 (Figure 1E) by fluorescence-activated cell sorting (FACS). Consistent with the expression pattern in E16 Crx-GFP^+^ mouse cells, Crx-GFP^+^ photoreceptors from d16 retinal organoids were significantly enriched for *Oc1* transcripts (2.5-fold over GFP^−^ cells), *Thrb2* and *Rxrg* (43- and 23-fold, respectively; N = 3, ^∗^p < 0.05, ^∗∗^p < 0.01; unpaired t test Figure 1E′). We next examined the appearance of proteins specific to developing cones. Immunostaining identified TRβ2 protein in the neural retina from d12, with the number of positive nuclei increasing until around d20 (3% ± 1.3% on d12 versus 18% ± 4.0% on d20, n > 10, N = 3; Figure 1F) in a pattern resembling in vivo retina (Ng et al., 2009). These cone precursors were present at least until d26 (∼P6 in vivo) in large numbers (20% ± 6%, n = 23, N = 3). Detection of the visual pigment protein S OPSIN and the phototransduction protein ARRESTIN3 suggested onset of maturation around d26 (Figure 1G). These data indicate sequential commitment to the cone lineage in our differentiation protocol equivalent to embryonic and early postnatal development.

### Stage-Specific Cone Genesis In Vitro Is Limited by Notch Signaling

**Figure 2:**
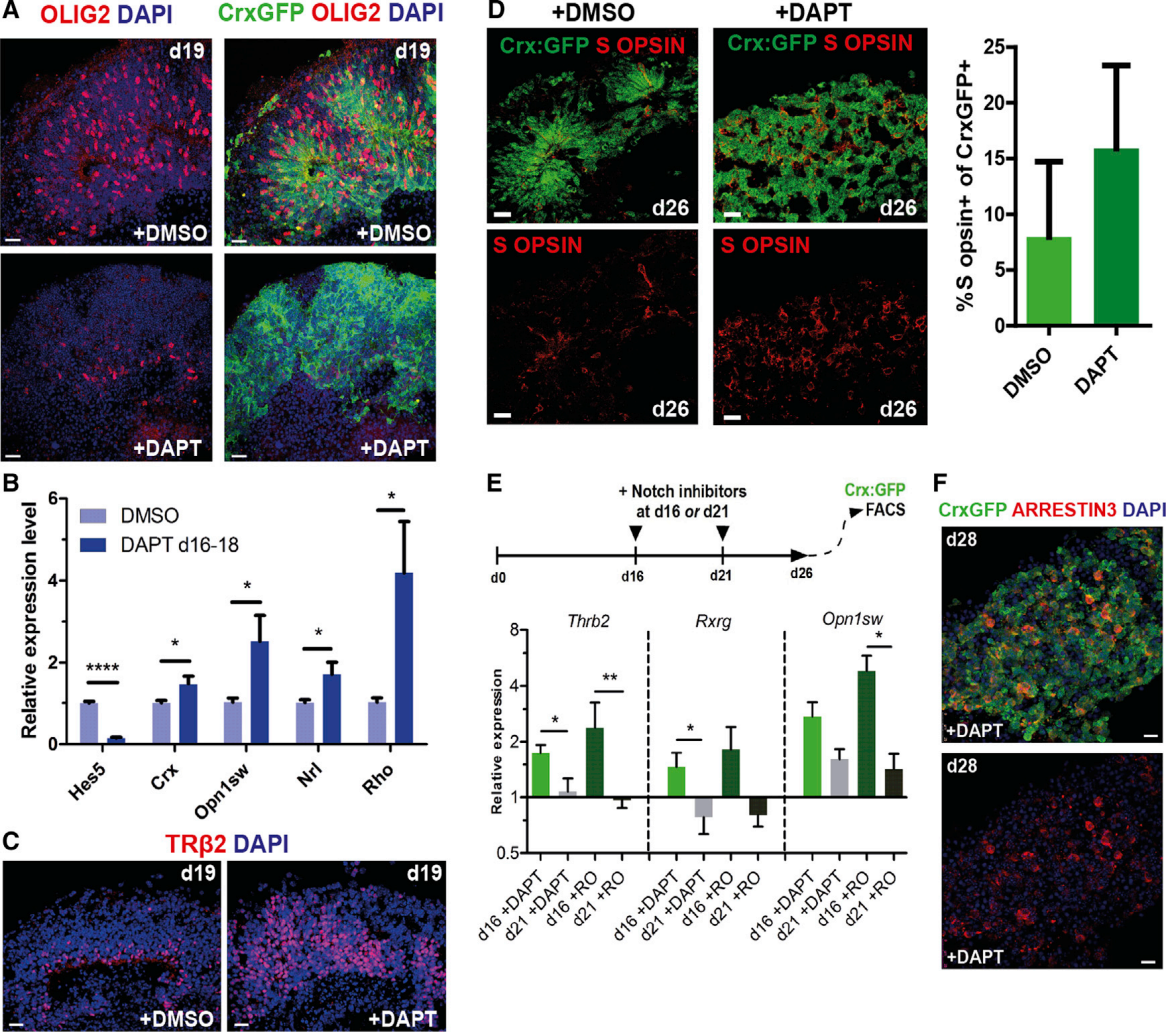
Notch Signaling Limits Developmental Stage-Restricted Cone Genesis (A) Fluorescent microscopy showing staining for OLIG2 and GFP reporter in control Crx-GFP retinal organoids (upper panel) and treated with 10 μM DAPT at days 16–18. Note the loss of OLIG2 staining following DAPT treatment. (B) qPCR gene expression analysis of control and DAPT-treated organoids at day 22, n = 5. Mean ± SD. ^∗^p < 0.05, ^∗∗∗∗^p < 0.0001, Student's t test. (C) Antibody staining for TRβ2 in control and DAPT-treated organoids. Note the increase in TRβ2^+^ nuclei. (D) S OPSIN immunostaining in control versus DAPT-treated Crx-GFP organoids. Quantification of the proportion of Crx-GFP^+^ cells co-expressing S OPSIN. N = 2, n > 30. Mean ± SD. (E) qPCR gene expression analysis of RNA samples from organoids treated with 10 μM DAPT or 10 μM RO4929097 at day 16 or day 21 of differentiation. Expression normalized to DMSO control cultures. N = 6. Mean ± SD. ^∗^p < 0.05, ^∗∗^p < 0.01, Student's t test. (F) Fluorescent microscopy showing expression of the Crx-GFP reporter and ARRESTIN3 at day 28 in an organoid treated with DAPT at day 16 of culture. All scale bars, 10 μm.

In the developing mouse retina, cone precursor birth is confined to the early phase of retinogenesis, between E11 and E19 (Carter-Dawson and LaVail, 1979). The number of progenitors exiting the cell cycle to give rise to cones is limited by the activity of NOTCH1 receptor, which maintains a sufficient pool of progenitors for the generation of other retinal neurons (Jadhav, 2006, Nelson et al., 2007, Yaron, 2006). Notch inhibition during the period of cone genesis should therefore raise the proportion of cone precursors. To determine whether this Notch-regulated program of neurogenesis is recapitulated in vitro, we treated the cultures with the Notch signaling inhibitor DAPT ((S)-tert-butyl 2-((S)-2-(2-(3,5-difluorophenyl)acetamido)propanamido)-2-phenylacetate) on days 16–18 of differentiation (∼E16–E18 in vivo, when cone genesis is still incomplete [Carter-Dawson and LaVail, 1979]). Treatment with 10 μM DAPT resulted in the loss of dividing progenitors (determined by a reduction in Ki67 and SOX9 staining, Figures S2A and S2B), including a reduction in OLIG2^+^ progenitor cells, which give rise predominantly to cones and rods (Figure 2A). These changes correlated with an increase in the proportion of postmitotic Crx-GFP^+^ photoreceptor precursors, detected by both GFP immunohistochemistry (Figures 2A and S2C) and flow cytometry on dissociated cultures (Figure S2D, N = 5), without affecting cell viability, but leading to some reduction in cell yields at d26 (Figures S3A–S3C, N = 3). qPCR analysis of gene expression in DAPT-treated cultures showed a significant reduction in the expression of the Notch target gene *Hes5* (6.7-fold, n = 5 RNA samples, 12 organoids each, N = 3, p < 0.0001, unpaired Student's t test) and an upregulation of cone and rod photoreceptor-specific genes, including *Crx*, *Opn1sw*, *Nrl*, and *Rho* (p < 0.05), compared with control cultures (Figure 2B). Immunohistochemistry showed increased immunoreactivity for TRβ2 (Figure 2C). Furthermore, the proportion of Crx-GFP^+^ cells co-expressing the cone visual pigment S OPSIN, which in control cultures was very similar to the early postnatal mouse retina (8% ± 7% at d26 versus 7% ± 2% at P8 in vivo; Figures S3D and S3E), was substantially increased (16% ± 7%, n > 30, N = 2; Figures 2D and S3E). The abundance of early born horizontal cells (CALBINBIN^+^) remained unchanged (Figures S3F and S3G); however, late progenitors/Müller glia (SOX9^+^) were significantly depleted (14 ± 2 versus 7 ± 1 per 10^4^ μm^2^ of organoid area; n = 26, N = 3, p = 0.0002, unpaired t test; Figures S3H and S3I). Together, this suggests that Notch inhibition had enhanced the level of cone differentiation at this stage.

To further examine the effect of Notch signaling on the timing of cone genesis, we treated cultures for 48 hr with 10 μM DAPT or 10 μM RO4929097 (another Notch signaling inhibitor) at either d16 or d21 (∼E16–E18 or ∼P1–P3 in vivo, respectively) and analyzed gene expression in Crx-GFP^+^ photoreceptors isolated and purified by FACS at d26 (Figure 2E). Transcripts of the cone-specific gene *Thrb2* were significantly more abundant (1.7- and 2.4-fold in cultures treated with DAPT and RO4929097, respectively; p < 0.05; p < 0.01, unpaired Student's t test) when Notch inhibition was performed at d16 of differentiation, compared with treatment at d21 (∼E16 versus ∼P2 in vivo). A similar pattern was detected for *Rxrg* and *Opn1sw* (Figure 2E), indicating a relatively higher level of cone differentiation following Notch signaling inhibition at an early, compared with a late, culture stage. Together, these data suggest a peak competence for cone genesis at an early phase of retinal histogenesis, as in vivo. Despite loss of laminar organization, further culture of organoids treated with DAPT at d16 led to expression of ARRESTIN3 (Figure 2F), RECOVERIN, and RHODOPSIN by d28 (Figure S2E), indicating that both cones and rods continue to mature after early Notch inhibition. Collectively, these observations demonstrate that mESC-derived retinal organoids respond to Notch signaling inhibition in a manner similar to that observed in vivo, and that timed application of Notch pathway inhibitors can be utilized to promote commitment to the cone fate.

### Knockdown of *Nrl* and *Nr2e3* Reveals Limited Plasticity of Postmitotic Photoreceptor Precursors

**Figure 3:**
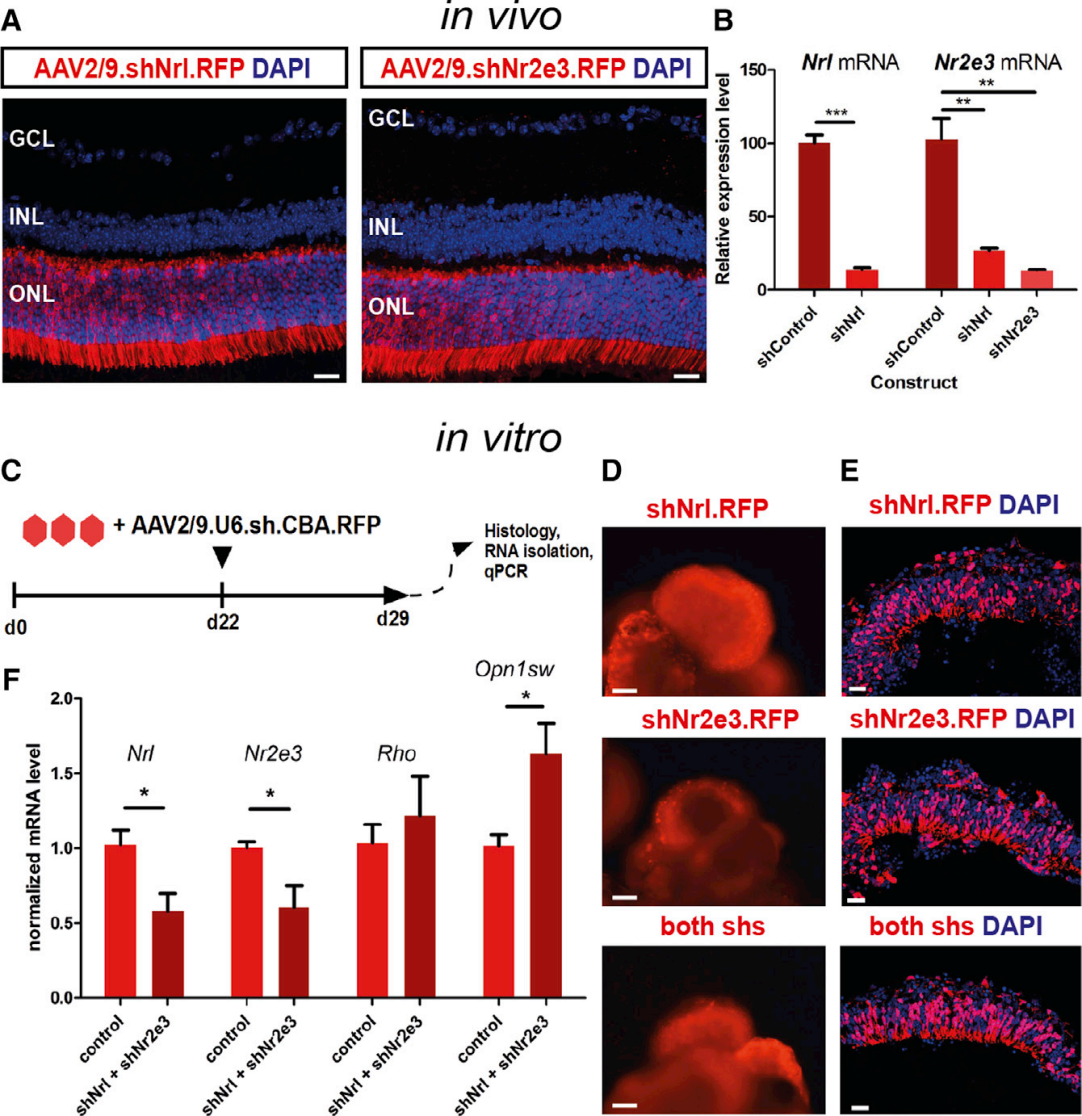
Knockdown of *Nrl* and *Nr2e3* Derepresses S OPSIN Transcription In Vitro (A) RFP fluorescent reporter expression following subretinal injection of AAV2/9 vectors encoding *shNrl* or *shNr2e3* driven by *U6* promoter and *CBA* promoter driving RFP transcription. GCL, ganglion cell layer; INL, inner nuclear layer; ONL, outer nuclear layer. Note confinement of transduction to the photoreceptor layer (ONL). Scale bar, 25 μm. (B) qPCR gene expression analysis of *Nrl* and *Nr2e3* mRNAs in RFP^+^ photoreceptors isolated by FACS normalized to non-targeting shControl-transduced photoreceptors; n = 3 retinas for each construct. ^∗∗^p < 0.01, ^∗∗∗^p < 0.001, Student's t test (C) Graphical depiction of the vector transduction experiment in vitro. (D and E) transduced organoids at day 29 showing expression of RFP. (D) Whole organoids; (E) sections through optic vesicle regions. Scale bars, 100 μm (D) and 20 μm (E). Mean ± SD. (F) qPCR expression analysis of *Nrl*, *Nr2e3*, *Rho*, and *Opn1sw* in infected versus control organoids. Note significant decreases in *Nrl* and *Nr2e3* associated with induction of S OPSIN (*Opn1sw*) expression. N = 4. Mean ± SD. ^∗^p < 0.05, Student's t test.

In early postmitotic photoreceptor precursors, NRL directs commitment to the rod lineage (Mears et al., 2001). Conversely, NRL loss of function leads to acquisition of an S cone-like photoreceptor phenotype (Mears et al., 2001) and has been utilized to obtain enriched populations of S cone-like cells for transplantation studies (Santos-Ferreira et al., 2015, Smiley et al., 2016). To determine whether mESC-derived photoreceptor precursors show an equivalent fate plasticity, with a view to generating cone-enriched populations for transplantation, we developed knockdown constructs targeting the coding sequences of *Nrl* and *Nr2e3* with an RFP reporter. Since these genes are only expressed in rod photoreceptor precursors, the effect of the knockdown is limited to postmitotic rods. These constructs mediated very efficient *Nrl* and *Nr2e3* transcriptional silencing, as assessed by qPCR, when packaged into AAV2/9 serotype viral vectors and delivered subretinally into adult mouse retina (Figures 3A and 3B; reduction by 86.2% and 86.5%, n = 3; p < 0.001 and p < 0.01, respectively, unpaired Student's t test). Retinal organoids were cultured as depicted in Figure 1A and transduced at d22 (∼P2 in vivo; Figures 3C–3E), at the end of peak of rod genesis and at which point relatively small numbers of actively dividing progenitors are present (Decembrini et al., 2014, Eiraku et al., 2011; Figures S4A and S4B). A significant increase in S OPSIN transcripts was detected in transduced versus control cultures (N = 4, p < 0.05, unpaired t test; Figure 3F), consistent with the S cone enrichment observed in vivo. However, consistent with an analysis of early postnatal *Nrl*^*−*/*−*^ mouse retina (Emerson et al., 2013), we could not detect upregulation of early cone differentiation genes *Thrb2* or *Rxrg* nor of a more mature marker *Arr3* in purified transduced photoreceptors, despite significant downregulation of *Nrl* and strong trends for *Nr2e3* and *Rorb* mRNA depletion (Figure S4G). There was no effect on RA signaling-related transcripts other than *Rorb* or the percentage of photoreceptors in culture (Figures S4F and S4H). Such an expression profile fits with the recent report describing evidence for S cone features during early rod development such as expression of S OPSIN transcripts in immature rods (Kim et al., 2016). Our results suggest that in cultures that received the *Nrl* and *Nr2e3* knockdown, developing rods remain at this immature stage, due to depletion of *Nrl* and *Nr2e3* transcripts. Thus, *Nrl*-depleted photoreceptors may represent an immature form of rods rather than true cone precursors, which may limit their utility in cone cell replacement.

### Retinoic Acid Signaling Regulates Cone Precursor Maturation in mESC-Derived Retinas

**Figure 4:**
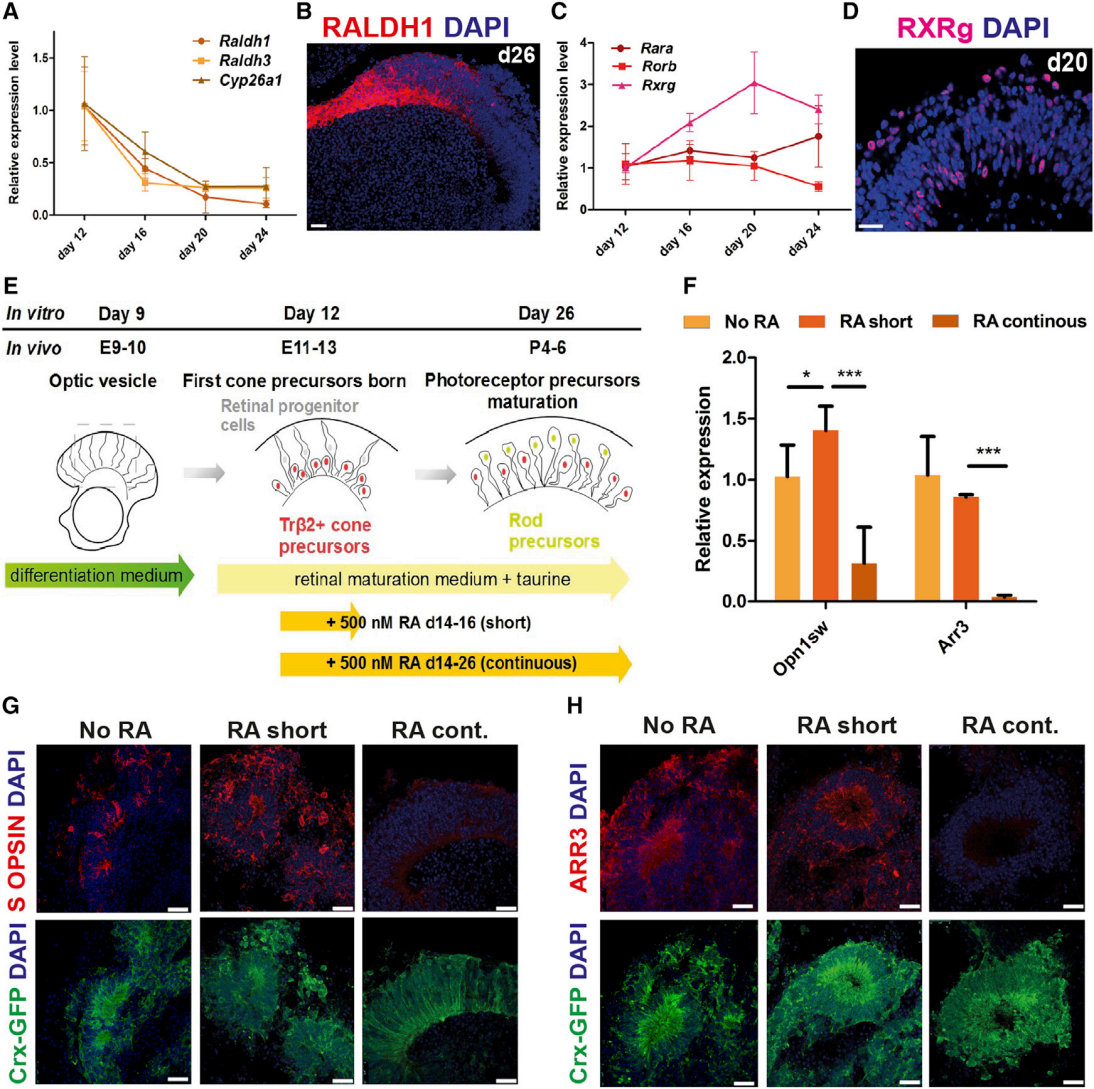
Cone Precursor Maturation in mESC-Derived Retinas Is Regulated by Retinoic Acid Signaling (A) qPCR expression time course of RA metabolism enzymes *Raldh1*, *Raldh3*, and *Cyp26a1* in mESC retinal organoids; n = 3. Mean ± SD. (B) Immunostaining for RALDH1 at day 26 in culture. (C) qPCR expression time course of RA receptors *Rara*, *Rorb*, and *Rxrg*; n = 3. Mean ± SD. (D) RXRγ antibody staining at day 20 of differentiation. (E) Graphical illustration of the RA stimulation experiments. (F) qPCR analysis of *Opn1sw* and *Arr3* expression at day 26 in cultures treated with RA as indicated. N = 4. Mean ± SD. ^∗^p < 0.05; ^∗∗∗^p < 0.001, Student's t test. (G and H) Fluorescent microscopy showing expression of cone-specific proteins S OPSIN (G) and ARRESTIN3 (H) and the Crx-GFP reporter in cultures treated as depicted in (E). Note the loss of cone marker immunoreactivity following continuous exposure to RA. All scale bars, 20 μm.

To optimize cone differentiation in vitro, we sought to determine further the extrinsic signals regulating this process, focusing on RA signaling as a candidate pathway. Synthesis of RA is high in embryonic murine retina, where it appears to induce S OPSIN expression but declines postnatally (Alfano et al., 2011, McCaffrery et al., 1993). Conversely, application of exogenous RA at a late stage of zebrafish photoreceptor development blocks cone maturation (Hyatt et al., 1996). We first examined expression of RA signaling components during in vitro retinogenesis by qPCR. RA metabolism enzymes *Raldh1*, *Raldh3*, and *Cyp26a1* showed strong downregulation from d12 onward (Figure 4A), whereas levels of the receptors *Rara*, *Rorb*, and *Rxrg* remained relatively stable (Figure 4C). Immunostaining confirmed RALDH1 and retinoid X receptor γ (RXRγ) expression, localized to the neural retina (Figures 4B and 4D, respectively). Given that RA at embryonic stages is critical for S OPSIN induction (Alfano et al., 2011), we tested the effects of supplementing cultures with a pulse of 500 nM all-*trans* RA at d14–d16 (∼E14–E16 in vivo; Figure 4E). This treatment significantly increased expression of the rod-specific transcription factor *Nrl*, but not the cone-specific genes *Thrb2* and *Rxrg*, by d21 in culture (Figure S5A). S OPSIN expression was upregulated by d26, but without change in the expression of *Arr3*, a cone phototransduction gene (Figure 4F). However, continued supplementation of RA (from d14 onward) strongly suppressed both S OPSIN and ARRESTIN3 expression by d26 (4.4- and 23.8-fold reduction, respectively, N = 4; ^∗^p < 0.05; ^∗∗∗^p < 0.001, Student's t test; Figure 4F). This transcriptional repression also correlated with decreased immunoreactivity for both S OPSIN and ARRESTIN3 in organoids continuously exposed to RA (Figures 4G and 4H). RA treatments did not affect cell viability, but early exposure to RA (d14–d16) modestly increased cell proliferation, determined by a bromodeoxyuridine pulse, which led to increased cell yields at later stages (Figures S5B–S5E). Despite the presence of S OPSIN^+^ and ARRESTIN3^+^ cells and expression of genes associated with cone maturation, including *Opn1mw* (Figures S5F–S5H), we did not observe M OPSIN protein by immunostaining in the organoids, even in prolonged culture (until d37). Collectively these data show that, as in development in vivo, temporal regulation of RA levels is important for onset of mESC-derived S cone precursor maturation and pulse administration of RA at a critical stage facilitates S OPSIN expression.

### M OPSIN Is Induced in mESC-Derived Cone Precursors Transplanted into the Adult Mouse Retina

**Figure 5:**
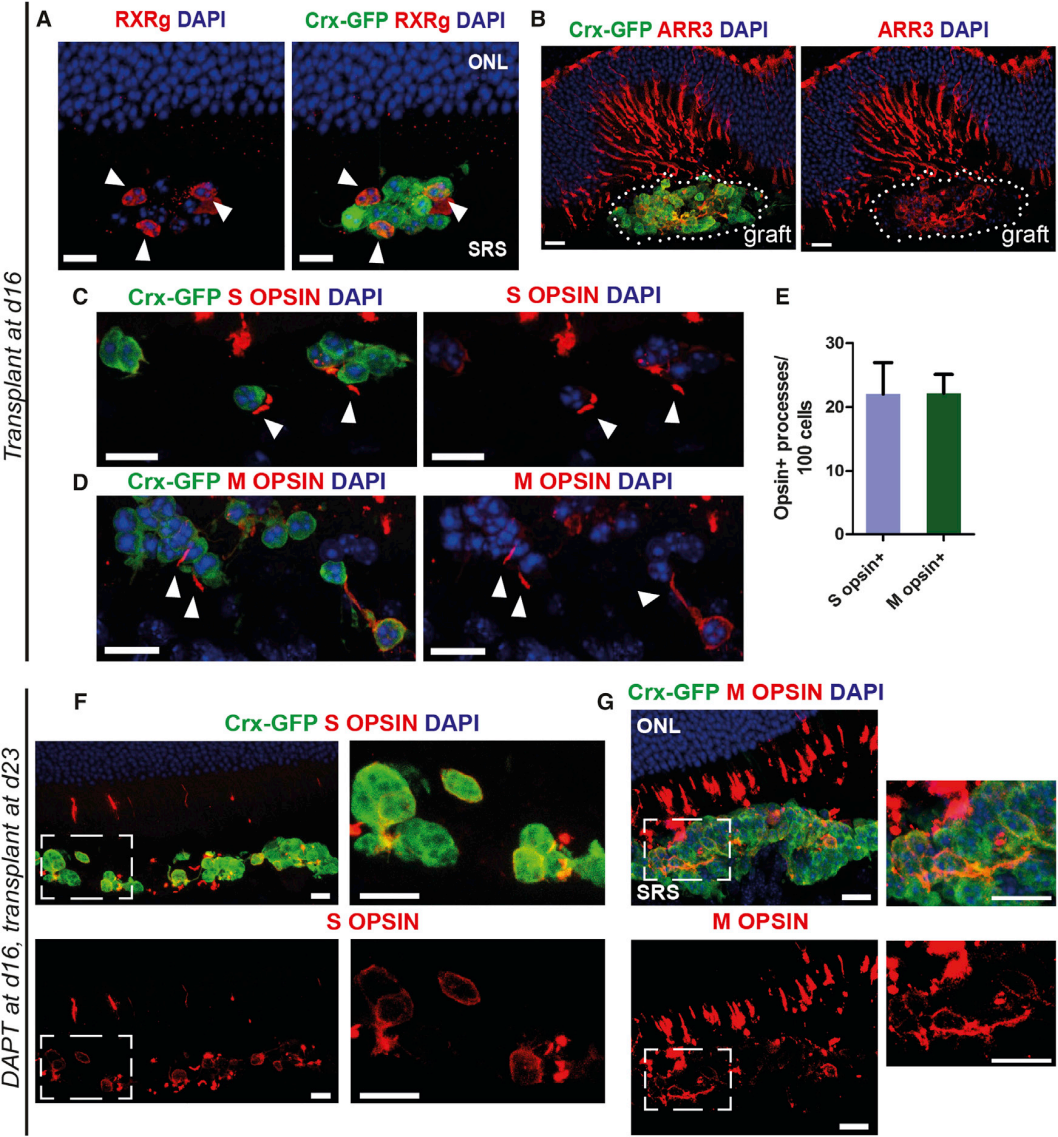
Cone Precursor-Enriched Populations Transplanted into Adult Retina Show Cone Maturation with M OPSIN Induction (A–D) Subretinal cells masses of transplanted mESC-derived Crx-GFP^+^ photoreceptors isolated at day 16 of differentiation. Immunostaining for RXRγ, a nuclear receptor specific for cones and ganglion cells (A, arrowheads), cone marker ARRESTIN3 (B), and cone visual pigments S OPSIN (C, arrowheads) and M OPSIN (D, arrowheads). Note that M OPSIN expression was not observed in vitro. (E) Assessment of S OPSIN- and M OPSIN-positive processes (arrowheads) in transplanted cells; n = 3 retinas. Mean ± SD. (F and G) S OPSIN (F) and M OPSIN (G) expression, respectively, in subretinal cell mass of Crx-GFP^+^ photoreceptors isolated at day 23 from organoids treated with 10 μM DAPT at day 16 in culture. Higher-power views of selected regions (boxed) are shown in right panels. ONL, outer nuclear layer; SRS, subretinal space. All scale bars, 10 μm.

M OPSIN in murine retina is expressed postnatally (Ng et al., 2001). Prolonged culture of our retinal organoids to a corresponding stage did not lead to M OPSIN protein induction. Since this limitation is also observed in mouse retinal explant cultures (Söderpalm et al., 1994), we sought to determine whether lack of M OPSIN expression is due to an inherent incapacity of the mESC-derived cone precursors or is a consequence of the in vitro conditions. In a previous study, we showed that the adult host retinal environment supported the maturation of transplanted Crx-GFP^+^ (mixed rod and cone) photoreceptor precursors derived from embryonic mouse retinas, including the expression of cone markers (Lakowski et al., 2010). We therefore isolated and purified by FACS Crx-GFP^+^ cells from mESC-derived retinal organoids at d16 (∼E16 in vivo) and injected them into the subretinal space of adult wild-type mice. When examined 3 weeks post transplantation, we observed that 35% (±18%) of surviving Crx-GFP^+^ cells co-expressed the all-cone marker RXRγ (n = 8 sections, N = 3 eyes; Figure 5A). In addition, ARRESTIN3 staining was present in the subretinal graft (Figure 5B). Notably, both S OPSIN^+^ (Figure 5C) and M OPSIN^+^ processes (Figure 5D) were formed within the subretinal space (three out of five grafts examined) in similar numbers (22 ± 15 and 22 ± 12 per 100 cells, n = 3; Figure 5E). To obtain a cone precursor-enriched population comparable with the early postnatal donors used previously for donor-derived cone-like cells (Santos-Ferreira et al., 2015, Smiley et al., 2016), we used Crx-GFP^+^ cells from d23 retinal organoids, which had been treated with DAPT at d16. As with the earlier staged cultures, Crx-GFP^+^ cells also survived and expressed S OPSIN and/or M OPSIN (Figures 5F and 5G), following transplantation into adult wild-type mice. We conclude that precursors derived from mESCs can undergo specification into both S OPSIN- and M OPSIN-expressing cones following transplantation into adult retina.

### Retinal Differentiation of mESCs Provides a Robust Source of Purified Cone Precursors for Transplantation

**Figure 6:**
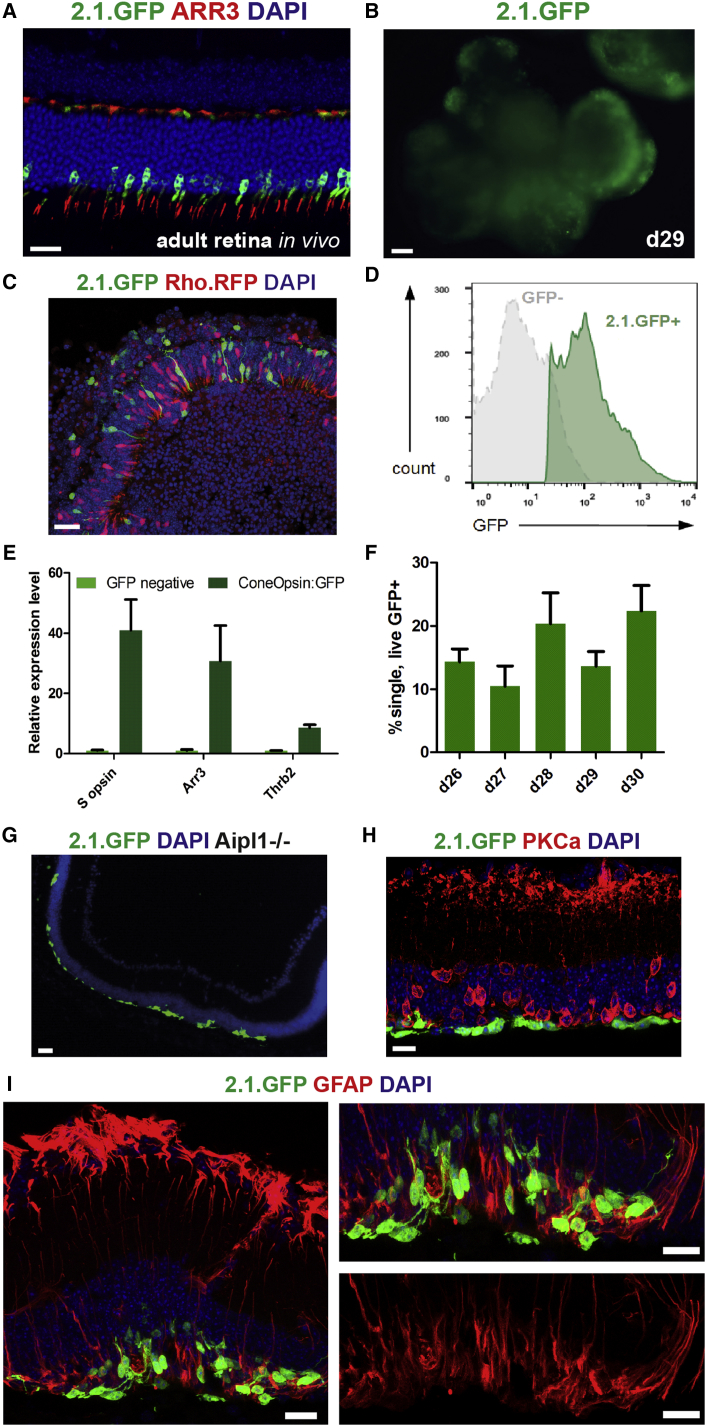
Isolation and Transplantation of Purified mESC-Derived Cone Precursors (A) Reporter fluorescence driven by AAV2/9-2.1.GFP vector following subretinal injection into adult mouse retina. Scale bar, 20 μm. (B) GFP fluorescence in whole retinal organoids at day 29 of differentiation infected with the AAV2/9-2.1.GFP vector at day 22. Scale bar, 100 μm. (C) 2.1.GFP-expressing cells are distinct from rods labeled with vector encoding RHODOPSIN promoter driving RFP. Scale bar, 20 μm. (D) Flow-cytometry histogram showing GFP fluorescence in dissociated cells from 2.1.GFP vector transduced organoids. (E) qPCR showing enrichment of cone genes *S opsin*, *Arr3*, and *Thrb2* in GFP^+^ population compared with GFP^−^ cells at day 26; n = 3. Mean ± SD. (F) Percentage of GFP^+^ cells. At least n = 3 sorts per time point. Mean ± SD. (G) 2.1.GFP^+^ cells in the *Aipl1*^*−*/*−*^ retina 3 weeks post transplantation. Scale bar, 20 μm. (H) MESC-derived donor cells overlie PKCα^+^ host bipolar cells. Scale bar, 10 μm. (I) Immunostaining for GFAP in a grafted retina. Note GFAP^+^ processes extending into the donor cell mass. Scale bar, 10 μm.

To assess the feasibility of transplanting purified cone-only populations from a renewable source, we used viral labeling to tag mESC-derived cones for isolation, purification, and transplantation. We used an AAV2/9 vector (2.1.GFP) encoding the 2.1-kb fragment of the human red-green (M/L) cone OPSIN promoter (Wang et al., 1992), which drives specific GFP expression in mouse cones (Figure 6A). Since the majority of mouse cones co-express both S OPSIN and M OPSIN (Applebury et al., 2000) the 2.1.GFP reporter was expected to label most mESC-derived mouse cones. Transduced retinal organoids showed GFP-labeled cells in the optic vesicle regions (Figure 6B). GFP^+^ cells were not proliferating progenitors (Figures S6A and S6B) or rod precursors (labeled with Rhodopsin.RFP virus; Figure 6C) and purified using FACS at d26 (Figure 6D) were highly enriched for the cone-specific transcripts *Opn1sw*, *Arr3*, and *Thrb2* (Figure 6E), supporting their cone precursor identity. We used our standard culture protocol without DAPT (Figure 1A), but including an RA pulse for transplantation, since no gain in absolute cone yield was observed with DAPT treatment (Figure S6C). Strikingly, the average percentage of total isolated cells expressing GFP^+^ at d26–d30 (∼P6–P10 in vivo) was 15% ± 7% (n = 21 sorts; at least three independent sorts per time point [Figure 6D] with typical viability around 80% [Figure S6D]). This is markedly higher than the ∼3% of photoreceptors that cones typically make up in the adult murine eye (Jeon et al., 1998). This high proportion of cone precursors allowed routine isolation in numbers sufficient for transplantation. We transplanted 2 × 10^5^ cells per eye, similar to the total number of cone cells in an adult mouse retina (Jeon et al., 1998). Initially, transplantation was performed into adult wild-type mice (n = 29 eyes) to determine the feasibility of purified mESC-derived cone transplantation in a non-degenerative environment. Our optimized methodology resulted in a high success rate, with 26 out of 29 eyes (89%) supporting the survival of the transplanted cell mass (Figures S6E–S6H), without immune suppression. Reporter-positive photoreceptors were present in the host outer nuclear layer (Figure S6H), but similar to previous studies demonstrated rod-like morphology and were likely the result of uptake of cellular components by host photoreceptors from the subretinal graft (Decembrini et al., 2017, Ortin-Martinez et al., 2016). In the subretinal space, the cells were negative for rod markers RHODOPSIN and GNAT1 (Figures S6E and S6F) and continued to develop in vivo, forming peanut agglutinin (PNA)-labeled extracellular matrix and expressing ARRESTIN3 (Figures S6G and S6H).

### Cone Replacement in *Aipl1*^*−*/*−*^ Mice by mESC-Derived Precursors

To determine whether in vitro differentiated cones could replace lost host cones in a model of end-stage retinal degeneration, we used *Aipl1*^*−*/*−*^ mice, which exhibit rapid photoreceptor loss (Ramamurthy et al., 2004). 2.1.GFP^+^ cone precursors were injected into 8-week-old *Aipl1*^*−*/*−*^ recipients and examined 3 weeks post transplantation. In all eyes examined, the donor cells survived (nine of nine injected eyes; Figure 6G) and were distributed over the remaining host interneurons (Figure 6H; PKCα^+^ bipolar cells in red). Close to the injection site the cells were clustered together and presented morphologies polarized toward the host retina, while further displaced cells were found with neurites extending more horizontally (Figures S6I and S6J). Müller cells, which support photoreceptor function, can become activated in retinal degenerations. In some types of disease they can form a glial scar that may impede donor/host interactions (reviewed by Pearson et al., 2014). Glial fibrillary acidic protein (GFAP), a marker of activated Müller cells, was upregulated in their basal processes (Figure 6I). Interestingly, at the interface between the graft and the host inner nuclear layer, we observed focal extension of Müller cell processes into the donor cell mass, potentially restoring the normal interaction between these two cell types (Figure 6I).

**Figure 7:**
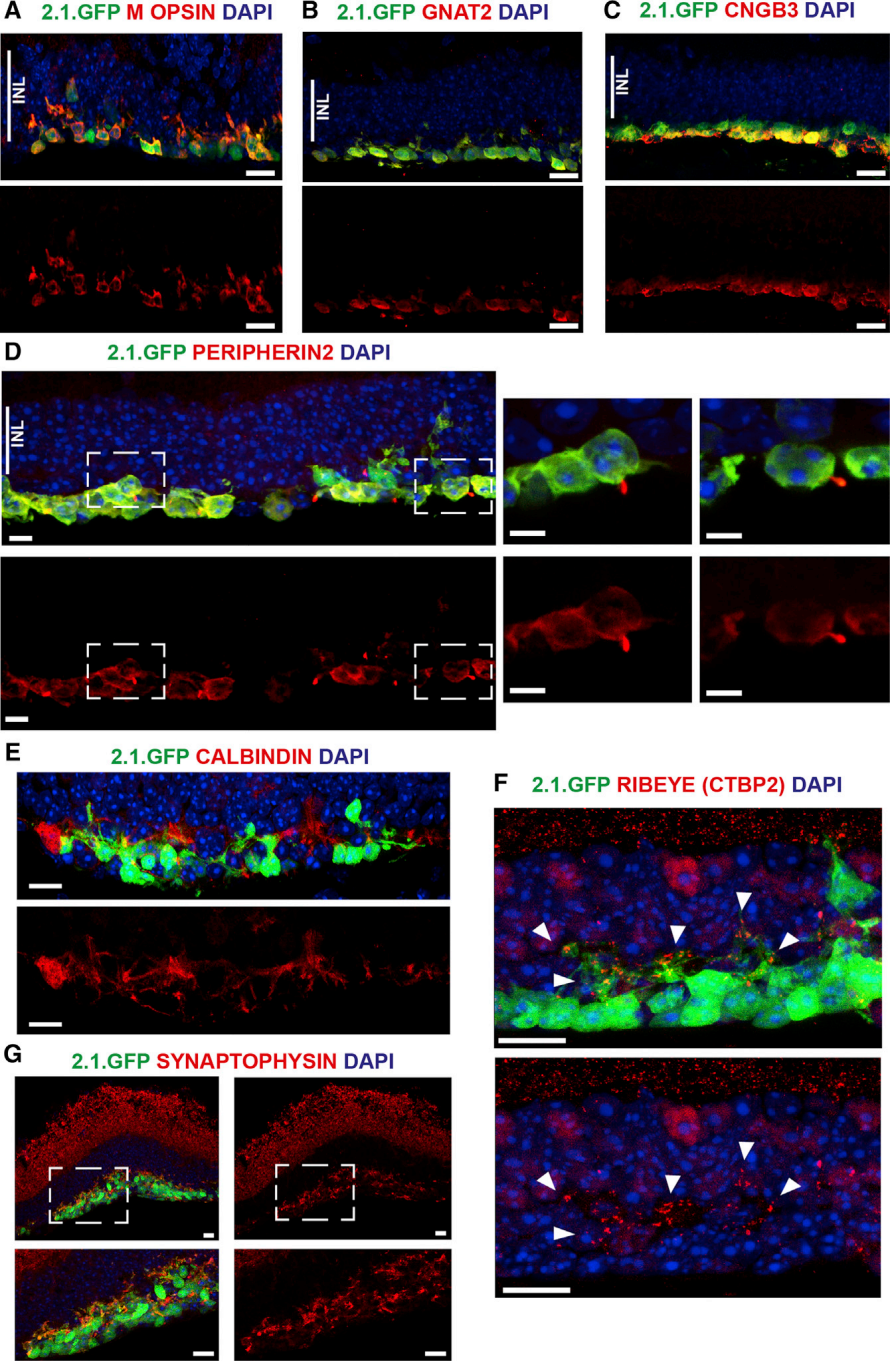
Cone Cell Replacement in Aipl1^−/−^ Retinas (A–C) Immunostaining for cone-specific phototransduction-related proteins M OPSIN (A), GNAT2 (B), and CNGB3 (C) in the transplanted 2.1.GFP^+^ cones. (D) PERIPHERIN2 staining showing accumulation in distal processes formed by transplanted cones. Panels on the right show a higher-power image of selected regions (boxed). (E) 2.1.GFP^+^ cells are in close proximity to CALBINDIN^+^ host horizontal cell dendrites. (F and G) Antibody staining for synaptic proteins RIBEYE (CTBP2; F) and SYNAPTOPHYSIN (G) showing expression in transplanted GFP^+^ cells (arrowheads in F). INL, inner nuclear layer. Scale bars, 5 μm (F) and 10 μm (all other images).

We next examined the in vivo maturation and potential connectivity of the transplanted cone precursors with the host retina. Expression of a panel of cone phototransduction-related proteins, including M OPSIN, GNAT2, and CNGB3 (Figures 7A–7C), was detected using immunostaining. PNA also labeled most of the transplanted cells (Figure S6K). Furthermore, donor cones formed distal processes directed toward the host retinal-pigmented epithelium, which were positive for the outer segment protein PERIPHERIN2 (Figure 7D), while basally directed neurites that extended toward the dendrites of host horizontal cells (Figure 7E) expressed the structural synapse protein, RIBEYE (Figure 7F), and the synaptic vesicle protein, SYNAPTOPHYSIN (Figure 7G). Collectively, these results indicate that mESC-derived cones may have the potential to replace lost host cones in a model of advanced retinal degeneration.

## Discussion

Retinal degenerations causing photoreceptor cell death are a major cause of untreatable blindness, for which photoreceptor replacement by transplantation may represent a promising treatment strategy. Transplanted dissociated photoreceptors can rescue visual function (Barnea-Cramer et al., 2016, Pearson et al., 2012, Santos-Ferreira et al., 2015, Singh et al., 2013). However, while useful human vision is mainly dependent on cone photoreceptors, most advances were made with rod photoreceptors. One of the reasons for this is the scarcity of cones, which, in the mouse, account for only around 3% of photoreceptors (Jeon et al., 1998), making their isolation in sufficient quantities for study problematic.

In this study, we combined mESC differentiation with progress in the understanding of retinal progenitor commitment to the cone photoreceptor lineage (Eiraku et al., 2011, Emerson et al., 2013, Hafler et al., 2012) to show that mESC-derived retinas exhibit competence to produce cone precursors and do so with high efficiency. Moreover, we find that timely inhibition of Notch signaling represents a more suitable approach for increasing cone proportion than silencing of the *Nrl*/*Nr2e3* rod differentiation pathway. Downregulation of *Nrl* only induced S OPSIN, whereas Notch inhibition additionally triggered increased expression of TRβ2 and RXRγ, permitting also M cone specification following transplantation. The high percentage of cone precursors generated in culture facilitated isolation and assessment of their transplantation potential. In vitro-generated cones showed reliable graft survival in the subretinal space of not only healthy recipients, but also in *Aipl1*^*−*/*−*^ host eyes with severely degenerated retina.

While characterizing in vitro differentiation of cone precursors we made several observations regarding this process. The proportion of cone precursors was high using our protocol, which might be a consequence of poor generation/survival of other retinal neurons including rods. Alternatively, a soluble factor acting as a negative feedback signal for cone genesis, analogous to the action of GDF11 in retinal ganglion cell development (Kim et al., 2005), may fail to reach biologically relevant concentrations. Timely inhibition of Notch suggested that the temporal competence confining the peak of cone genesis to early stages of retinogenesis (Carter-Dawson and LaVail, 1979) is preserved in mESC-derived retinas differentiated in vitro. Notch inhibition may increase commitment to cone fate or accelerate their differentiation. While the proportion of cones was increased at the time point used for transplantation (equivalent to early postnatal stage), there was no significant gain in total yield. Furthermore, we found that RA signaling plays an evolutionarily conserved role in regulating photoreceptor maturation. The addition of exogenous all-*trans* RA to late stages of photoreceptor differentiation in ESC-derived retinas led to the suppression of cone maturation. This is similar to observations that exogenous RA impairs cone maturation in the fish retina (Hyatt et al., 1996). Expression of enzymes responsible for RA synthesis declined significantly at late differentiation stages, in accordance with previously reported biochemical analyses showing RA to be abundant at early retinogenesis in vivo (when cones are born) but declining neonatally (when rods are produced [McCaffrery et al., 1993]). High levels of RA in embryonic retina might therefore act to prevent precocious cone maturation during embryonic development, while acting to stimulate rod differentiation (as also observed in mESC-derived retinas) in the neonatal retina. This could provide a mechanism to synchronize maturation of the two photoreceptor types despite the lag between their birth peaks.

When isolated from their differentiation niche and transplanted into an adult environment, mESC-derived cone precursors at equivalents of both embryonic and early postnatal stages are able to undergo further differentiation in vivo, similar to precursor cells derived from donor mice (Lakowski et al., 2010). Strikingly, many of these cells go on to express M OPSIN, even in the severely degenerate *Aipl1*^*−*/*−*^ retina. The signals present in the mature host retina appear to trigger M OPSIN expression, not observed in vitro despite extended culture periods. This suggests that the developmental capacity for subsequent M cone differentiation is retained in the retinal organoid system, but certain inductive signals, such as thyroid hormone provision from the circulation (Lu et al., 2009), are likely missing.

Using a mESC retinal organoid system as a source, we could reproducibly transplant cones in numbers on a par with the total number of cones in an adult mouse retina (Jeon et al., 1998). We achieved extensive cone cell replacement in the *Aipl1*^*−*/*−*^ mouse, a model of Leber congenital amaurosis end-stage retinal disease in which the vast majority of host cones are lost due to degeneration (Ramamurthy et al., 2004). Unsurprisingly, given the advanced degenerative state of the recipient retina, the morphology of transplanted cones was compromised. This contrasts with apparently mature features of GFP-labeled cells observed in cone transplants into non-degenerative retina, which are now understood to arise from cytoplasmic material transfer from the subretinal graft to host rods (Decembrini et al., 2017, Ortin-Martinez et al., 2016). Nonetheless, cones in the *Aipl1*^*−*/*−*^ recipients appeared to make physical contact with inner retinal neurons and expressed components associated with synaptic transmission, alongside phototransduction-related proteins, suggesting advanced differentiation and maturation. Although beyond the scope of the present study, future studies will be required to investigate further the potential functionality of these transplanted cells.

In summary, we report the robust differentiation and subsequent transplantation of mESC-derived cone precursors (summarized in Figure S7). Cones differentiated in vitro following a specification sequence resembling that observed in vivo, indicating that mESC-derived retinal organoids represent a developmentally relevant donor source for investigating cone cell replacement. Following isolation and transplantation, these cells survived and showed a degree of maturation in photoreceptor-depleted retina of *Aipl1*^*−*/*−*^ mice, a model of end-stage retinal degeneration. Our work provides an important proof of concept for future use of purified cone photoreceptor transplantation to treat central vision loss due to cone cell death.

## Experimental Procedures

In all experiments, n indicates the number of individual samples and N the number of independent repeats, as with separate differentiation batches. Full details of experimental methods are provided in Supplemental Experimental Procedures.

### Mouse ESC Culture and Retinal Differentiation

E16 CEE line was maintained on gelatin-coated dishes in the presence of leukemia inhibitory factor (1,000 U/mL; Merck Millipore). Crx-GFP line was cultured in “2i medium” supplemented with 3 μM GSK-3 inhibitor CHIR99021 (Tocris Bioscience) and 10 μM MEK inhibitor PD0325901 (Tebu-bio). The differentiation was performed as previously described (Decembrini et al., 2014, Gonzalez-Cordero et al., 2013). In brief, 3,000 mESCs were added to each well of Nunclon Sphera ultralow-binding 96-well plates (Thermo Fisher Scientific) to form an embryoid body (EB). Growth factor-reduced Matrigel (VWR International) was added next day at a final concentration of 2%. On day 9, EBs were transferred into 24-well plates (12 EBs per well) for further culture. A modification of our previous protocol was supplementing cultures with 500 nM all-*trans* RA (Sigma-Aldrich) between days 14 and 16 only. Normoxic conditions were used throughout the culture period. γ-Secretase inhibitors DAPT (Merck Millipore) and RO4929097 (BioVision) or DMSO as a vehicle control were added at a final concentration of 10 μM.

### Immunohistochemistry

Organoids were fixed for 30 min in 4% formaldehyde, washed with PBS, and incubated overnight in 20% (w/v) sucrose, prior to embedding in OCT matrix. Tissue was cut into 18-μm cryosections mounted on glass slides, air-dried for 20 min, and kept frozen at −20°C for use in immunostaining. Staining protocol and the antibodies used are listed in Supplemental Experimental Procedures.

### Dissociation and Fluorescence-Activated Cell Sorting

Organoids were dissociated using a papain-based Neural Tissue Dissociation Kit (Miltenyi Biotec) prior to sorting on a BD Influx Cell Sorter (Becton Dickinson). Flow-sorted GFP^+^ cells were on average >95% pure and >80% viable. Cells were resuspended at a final concentration of 1 × 10^5^ live cells/μL in sterile Hank’s balanced salt solution (+Ca^2+^, +Mg^2+^) and DNase I (50 U/mL) before injection.

### Transplantation of mESC-Derived Photoreceptors

Full details of the surgical procedure can be found in Supplemental Experimental Procedures. In brief, 1 μL of cell suspension was injected into the superior retina and another 1 μL into the inferior retina. Adult mice were between 8 and 16 weeks of age at the time of transplantation. All animals were housed under a normal 12/12-hr light/dark cycle. Eyes were harvested 3 weeks after transplantation.

## Author Contributions

K.K. contributed to the conception, design, execution, and analysis of all experiments and writing of the manuscript; A.G.C. contributed to the design and analysis of a number of the experiments; D.G. contributed to the analysis of a number of the experiments; A.N., M.J., S.J.I.B., and M.K., contributed to the maintenance and differentiation of stem cell cultures; Y.D. contributed to histological processing and surgery; A.G. contributed to the design of experiments; R.D.S. contributed to FACS and flow cytometer analysis; R.N.M performed viral purification; A.J.S. contributed interpretation of experiments and manuscript writing; S.D. and Y.I. provided reagents and contributed to revision of the manuscript; J.C.S. contributed to revision of the manuscript and funding; R.A.P. contributed to the conception, design and interpretation of experiments, subretinal surgery, manuscript writing, and funding; E.L.W. contributed to the conception, design and interpretation of experiments, and manuscript writing; R.R.A. contributed to the conception, design and interpretation of experiments, manuscript writing, and funding.

## Supplemental Information

Document S1. Supplemental Experimental Procedures, Figures S1–S7, and Tables S1–S4

Document S2. Article plus Supplemental Information
