# Supplementary material for: Differentiation and Transplantation of Embryonic Stem Cell-Derived Cone Photoreceptors into a Mouse Model of End-Stage Retinal Degeneration
Source: Stem Cell Reports. 2017 May 25;8(6):1659–74. doi: 10.1016/j.stemcr.2017.04.030 (PMC5470175; doi:10.1016/j.stemcr.2017.04.030)
Supplement: Document S1. Supplemental Experimental Procedures, Figures S1–S7, and Tables S1–S4 [file mmc1.pdf]

Stem Cell Reports, Volume 8

Supplemental Information

Differentiation and Transplantation of Embryonic Stem Cell-Derived Cone Photoreceptors into a Mouse Model of End-Stage Retinal Degeneration

Kamil Kruczek, Anai Gonzalez-Cordero, Debbie Goh, Arifa Naeem, Mindaugas Jonikas, Samuel J.I. Blackford, Magdalena Kloc, Yanai Duran, Anastasios Georgiadis, Robert D. Sampson, Ryea N. Maswood, Alexander J. Smith, Sarah Decembrini, Yvan Arsenijevic, Jane C. Sowden, Rachael A. Pearson, Emma L. West, and Robin R. Ali

Supplemental Figures

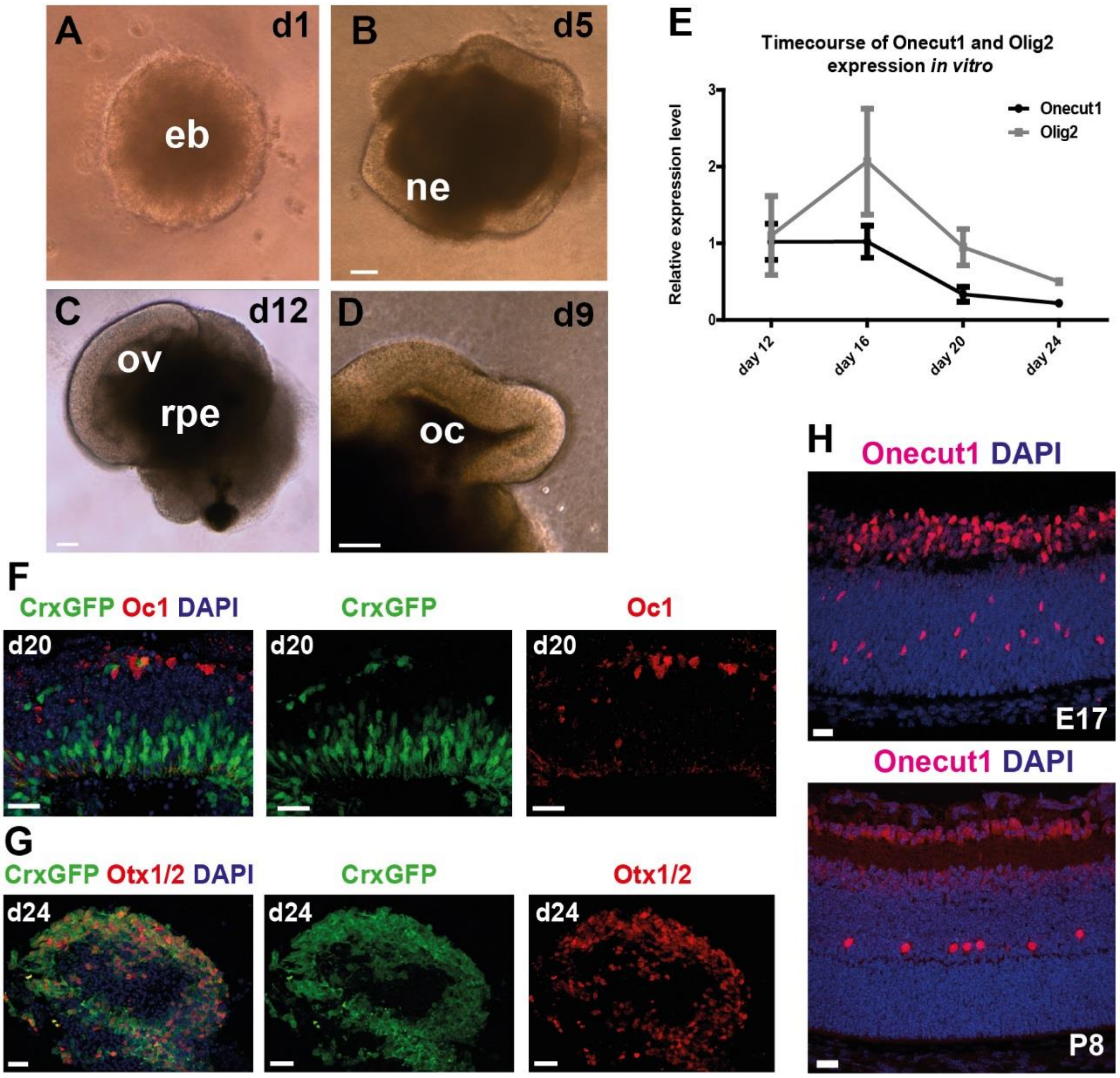

Figure S1. Differentiation of mESC into optic tissue expressing markers of cone-biased retinal progenitors. Related to Figure 1.

(A-D) Retinal differentiation cultures. Organoids at the stage of embryoid body (d1; A), retinal neuroepithelium formation (d5; B), optic cup (d9; C) and evaginated optic vesicle (d12; D). Scale bars 100 µm. **(E)** QPCR mRNA expression timecourse in cultures for Onecut1 and Olig2. Expression levels normalized to d12 of differentiation. **(F)** Immunostaining for Onecut1 (Oc1) in Crx-GFP mESC line retinal neuroepithelium at d20. Note that expression of Onecut1 is primarily localized to the basal side of the tissue rather than apical, where developing Crx-GFP+ photoreceptor precursors reside. Scale bar 20 µm. (**G**) Immunostaining for Otx2 in Crx-GFP line organoids at d24. Extensive overlap in immunoreactivity is observed in the retinal neuroepithelium at this stage. Scale bar 20 µm. **(H)** Onecut1 immunostaining in developing retina. Staining becomes restricted to horizontal cells by P8 (right panel). Scale bar 10 µm. eb – embryoid body; ne – neuroepithelium; oc – optic cup; ov – optic vesicle; rpe – retinal pigment epithelium.

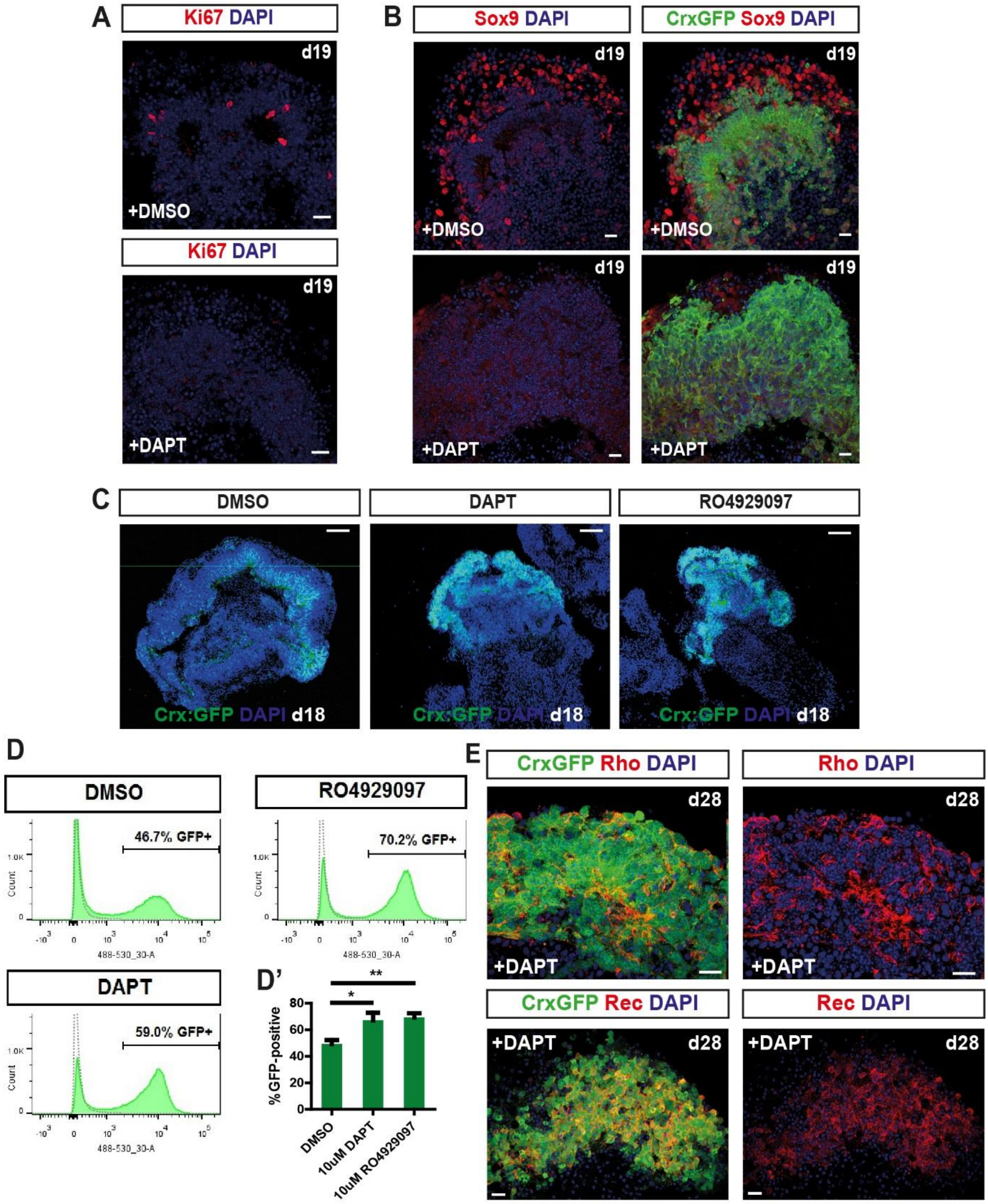

Figure S2. Regulation of photoreceptor neurogenesis by Notch signalling. Related to Figure 2.

(A) Loss of Ki67 staining following treatment with Notch signalling inhibitor DAPT at 10 µM between d14-d16. Scale bar 10 µm. **(B)** Reduction in immunostaining for retinal progenitor marker Sox9 following DAPT treatment. **(C)** Induction of Crx-GFP reporter expression at d18 in organoids treated with 10 µM DAPT or 10 µM RO4929097 at d14-d16. Scale bars 100 µm. **(D)** Representative flow cytometry histograms showing the proportion of Crx-GFP+ photoreceptor precursors following Notch inhibitor treatment in dissociated cultures at d19. **(D')** Quantification of flow cytometry results. N=5 experiments, >30 organoids per sample per experiment. *, $p<0.05$; **, $p<0.01$ one-way ANOVA with Tukey's *post hoc* test. **(E)** Rhodopsin and Recoverin immunostaining at d28 of differentiation in Crx-GFP retinal organoids treated with 10 µM DAPT at d16. Note the presence of these maturation markers despite loss of laminar organisation. Scale bar in all panels 10 µm.

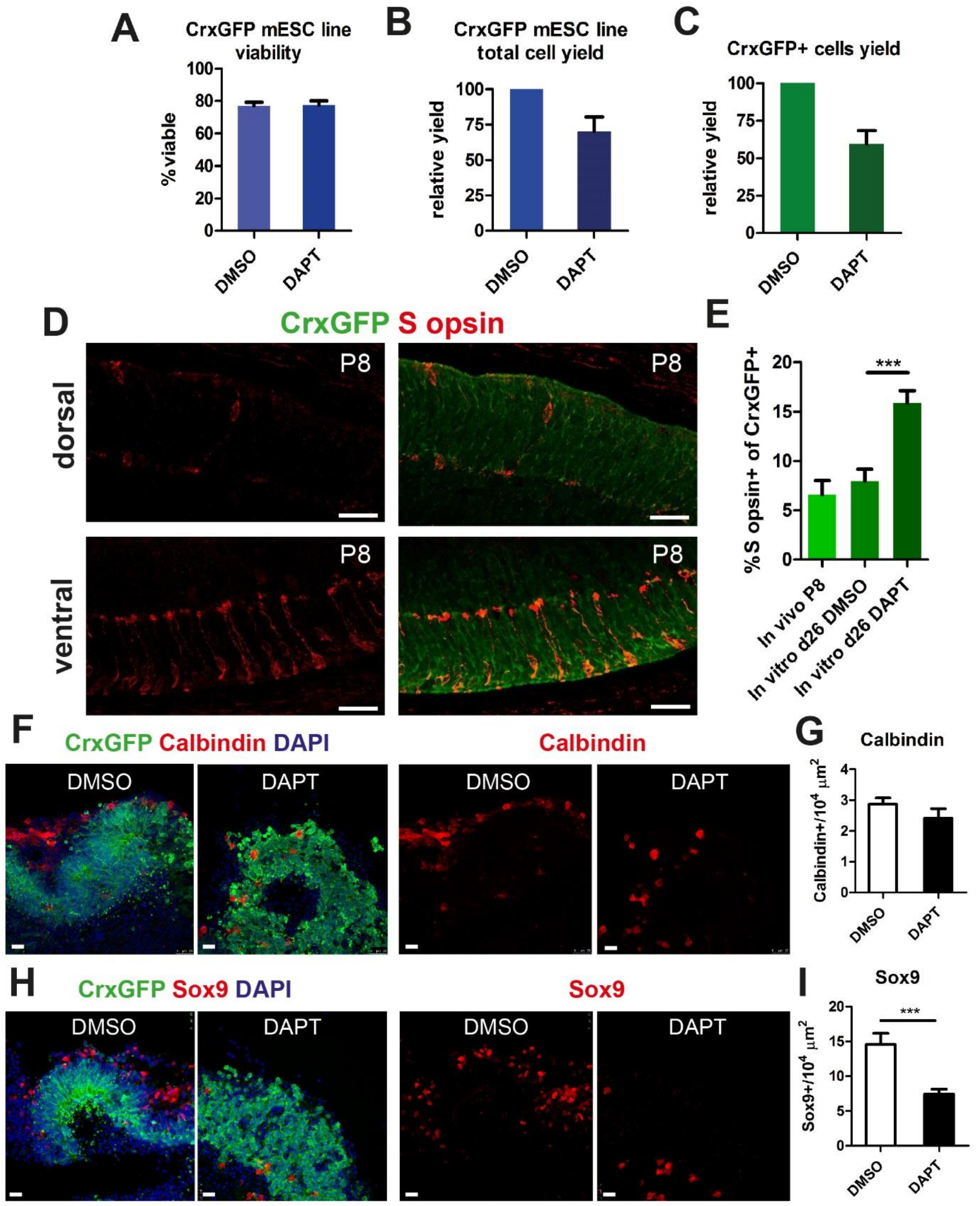

Figure S3. Impact of Notch inhibition on abundance of retinal neuron populations. Related to Figure 2. (A) Viability of dissociated control and DAPT-treated cultures. N=3, 36 organoids per experiment, mean ±SEM. **(B)** Cell yield determined by flow cytometry and viability analysis normalised to the DMSO control samples. N=3, 36 organoids per experiment, mean ±SEM. **(C)** Crx-GFP+ cell yield for the samples in (B). **(D)** Expression of S opsin in P8 retina in vivo. Upper panels dorsal region, lower panels ventral region. **(E)** Quantification of S opsin+ cells in P8 retina in vivo. N=3 eyes from individual animals, n=9 sections per eye from dorsal, mid-central and ventral parts of the retina. Data from retinal organoids from Fig.2 presented for comparison. **(F-I)** Immunostaining (F,H) and quantification (G,I) of calbindin+ horizontal cells (F,G) and Sox9+ late progenitors/ Müller glia (H,I). Scale bar 10 μm. Cell number normalised to organoid area; mean ±SEM, n>25, N=3, p<0.001 unpaired Student's t-test.

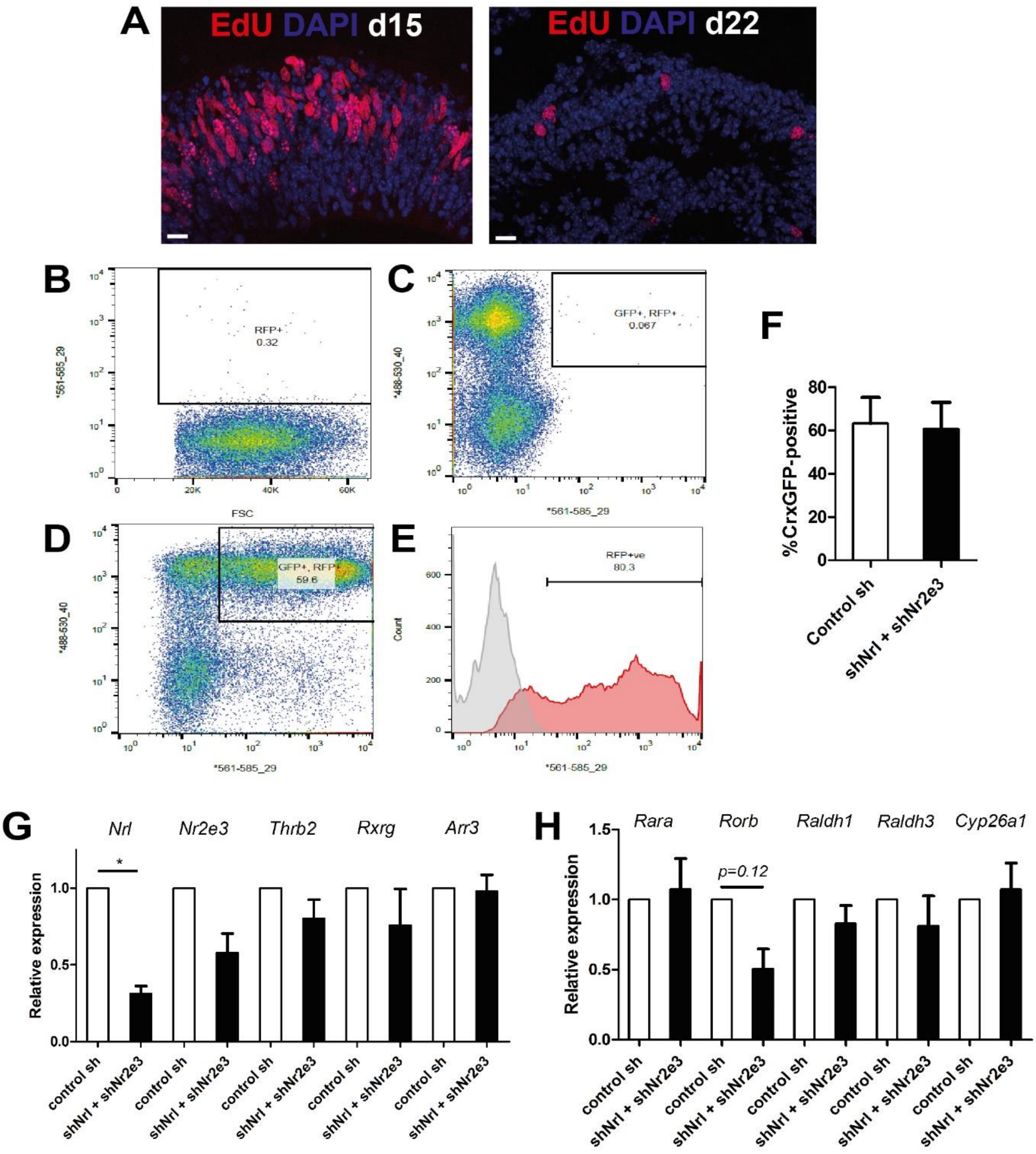

Figure S4. Knockdown of *Nrl* and *Nr2e3* in isolated photoreceptor precursors. Related to Figure 3.

(A) EdU pulse immunodetection in retinal organoids at d15 or d22. 10 µM EdU was added to culture media for 1 hr before harvest. Note the low number of proliferating cells at d22. Scale bar 10 µm. **(B-E)** Isolation and purification of post-mitotic photoreceptor precursors transduced with Nrl and Nr2e3 knockdown constructs by FACS. (B,C) Representative scatter plots showing gating strategy for collection of RFP+ (B) and GFP+ /RFP+ double positive cells (C) using non-transduced Crx:GFP dissociated control cultures. (D) Representative scatter plot showing collection of Crx:GFP photoreceptor precursors expressing shNrl and shNr2e3 vectors. Note the presence of a distinct GFP+RFP+ population. (E) Histogram showing RFP expression in Crx:GFP photoreceptor precursors transduced with shNrl and Nr2e3 vectors. **(F)** Percentage of Crx-GFP cells determined by flow cytometry in the knockdown experiments. N=4 samples analysed. **(G, H)** QPCR gene expression analysis of photoreceptor-specific genes (G) and RA signalling-related transcripts (H) using RNA isolated from flow-sorted either control or knockdown construct transduced CrxGFP+ photoreceptor precursors; n=7 samples, N=6 differentiation cultures; *, $p<0.05$, Wilcoxon matched pairs test.

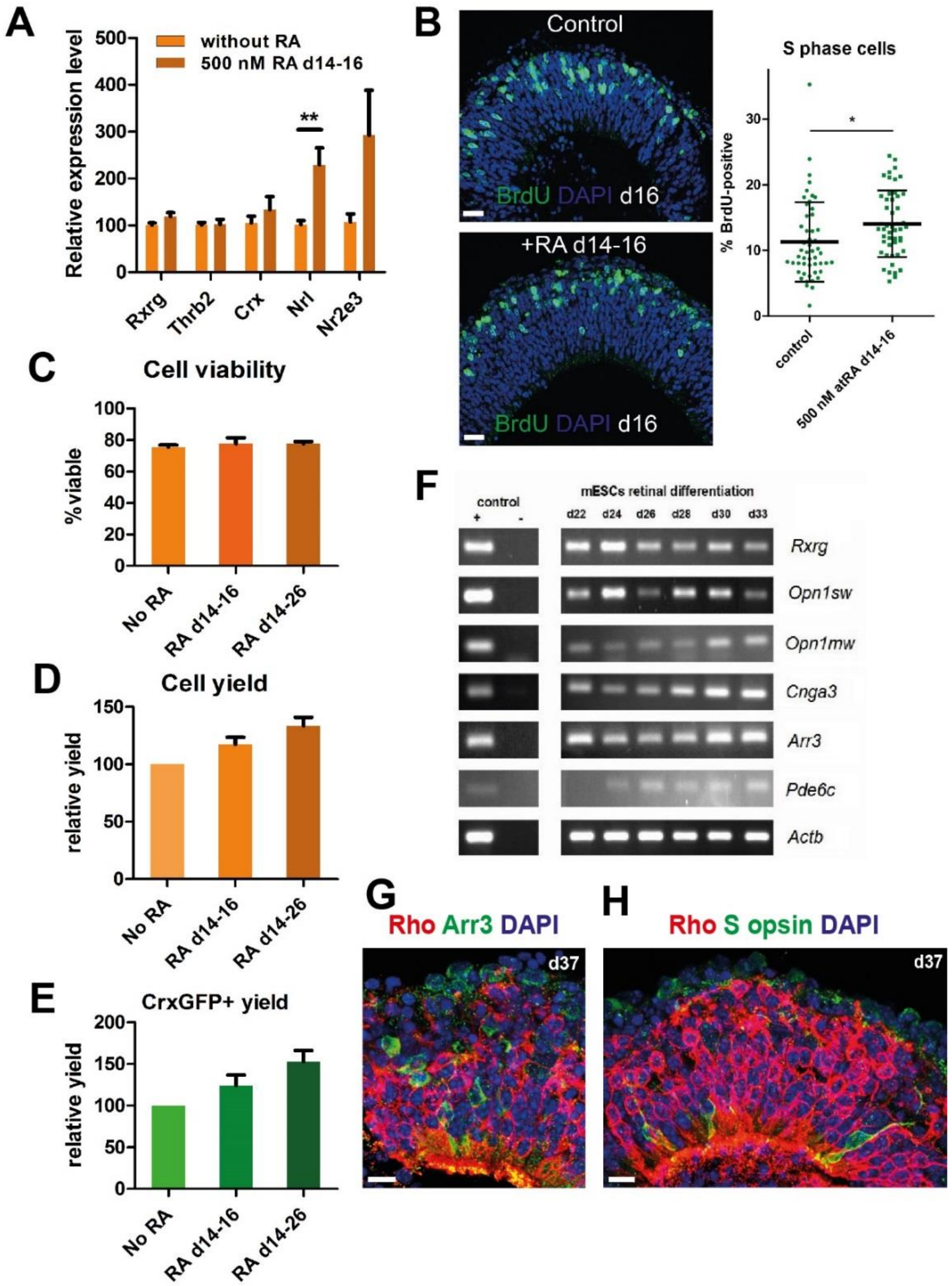

Figure S5. Retinoic acid supplementation and retinal organoid development. Related to Figure 4.

(A) QPCR analysis of cultures treated with a pulse of 500 nM RA at d14-d16 normalized to control cultures without the supplementation. Note the induction of *Nrl* and *Nr2e3* without effect on *Thrb2*, *Rxrg* or *Crx*; n=6 samples from N=3 differentiation cultures. **, $p<0.01$, Student's *t*-test. **(B)** BrdU pulse proliferation analysis of cultures treated with RA at d14-16. 10 µM BrdU was added to culture media for 1 hr before harvest. Sections immunostained for BrdU in left panels. Scale bar 20 µm. Right panel shows quantification of of BrdU+ nuclei, n>40, N=3, bars indicate mean ±SD, $p<0.05$, unpaired Student's *t*-test. **(C)** Cell viability in dissociated RA-treated cultures. N=3, 36 organoids each sample, mean ±SEM. **(D,E)** Live cell yields determined by viability assay and flow cytometry. (D) Total cell yield. (E) Crx-GFP+ cell yield. N=3, 36 organoids each sample, mean ±SEM. **(F)** RT-PCR analysis of differentiation cultures at post-natal development equivalents d22 (~P2) to d33 (~P13) for cone-specific genes. Negative controls are no template reactions, P8 eye RNA used for positive controls. **(G,H)** Immunostaining for rod (Rhodopsin in red) and cone-specific (Arrestin3 in C and S opsin in D, in green) proteins at d37 of differentiation. Scale bar 10 µm.

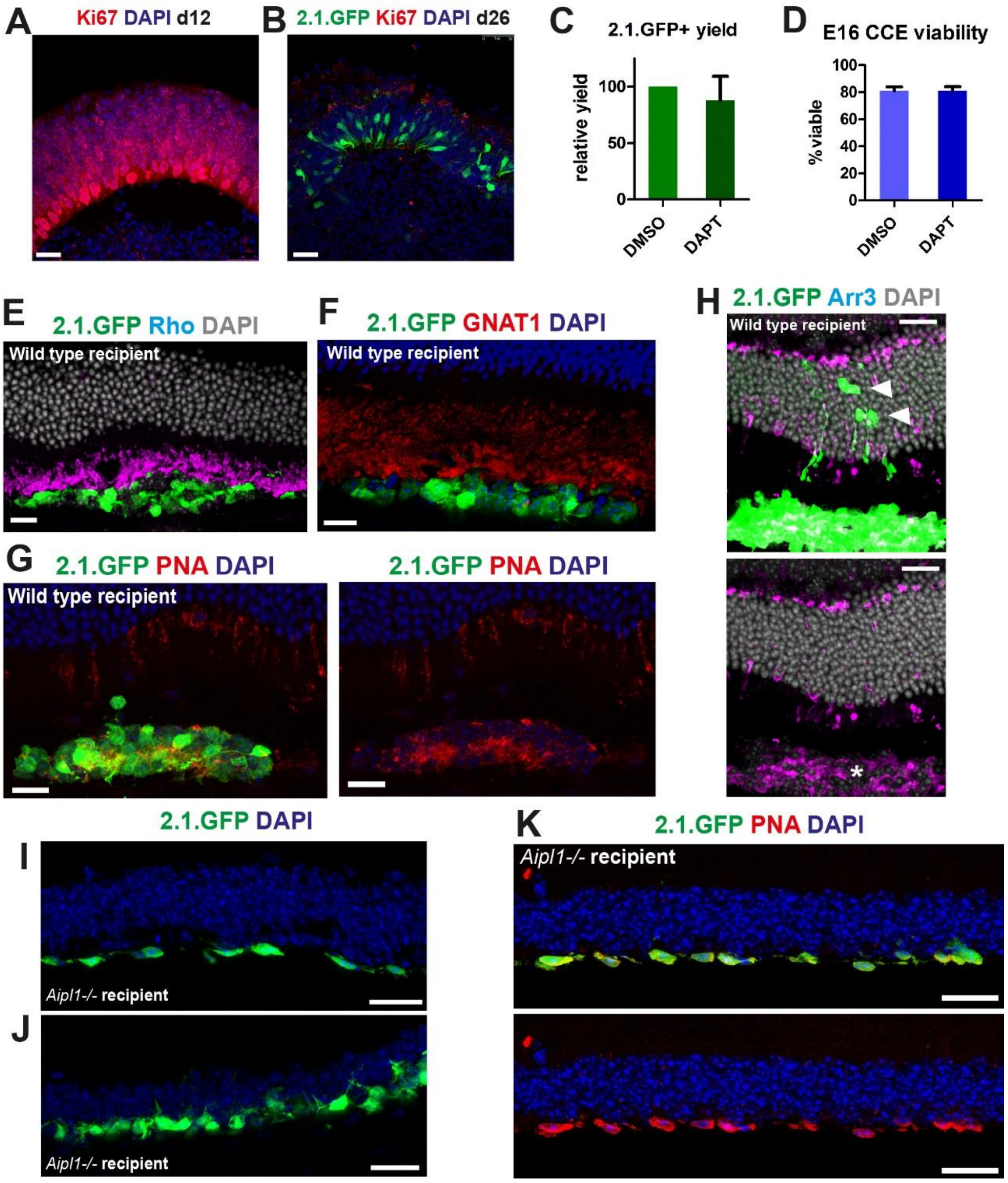

Figure S6. MESC-derived cone precursor transplantation. Related to Figures 6 and 7.

(A,B) Ki67 staining in d12 (A) and 2.1.GFP-transduced d26 organoids. Note loss of Ki67 by d26 in culture. Scale bar 20 µm. **(C)** Cell viability analysis in cultures dissociated according to the protocol used for transplantation. N=3, 36 organoids each sample, 36 organoids each sample, mean ±SEM. **(D)** Live cell yield normalised to DMSO control sample for DAPT-treated cultures in (C) determined by flow cytometry. **(E-H)** Immunostaining for rod, (Rhodopsin and Gnat1, E and F, respectively) and cone (PNA and Arrestin3, G and H, respectively) markers in wildtype host eyes transplanted with 2.1.GFP-expressing cone precursors at 3 weeks post-transplantation. Note the absence of rod-specific proteins Rhodopsin (E, in cyan) and Gnat1 (F, in red) and the presence of cone-specific markers PNA (G, in red) and Arrestin3 (H, in cyan, * indicates the cell mass) in the GFP+ subretinal cell mass. Arrows in H point to GFP+ cells with rod morphology in the host outer nuclear layer that most likely are a result of cytoplasmic material transfer from the cell graft. Scale bars in all images 20 µm. **(I,J)** Morphologies of transplanted cone precursors in the *Aipl1*$^{-/-}$ recipient retina. (I) dispersed cells aligned with neurites extending horizontally along the interneuron layer. (J) In higher density cells showed more extensive neurite outgrowth and polarization. **(K)** Transplanted cells secrete PNA-staining extracellular matrix *in vivo*. Scale bars in all images 25 µm. Samples collected 3 weeks post-transplantation.

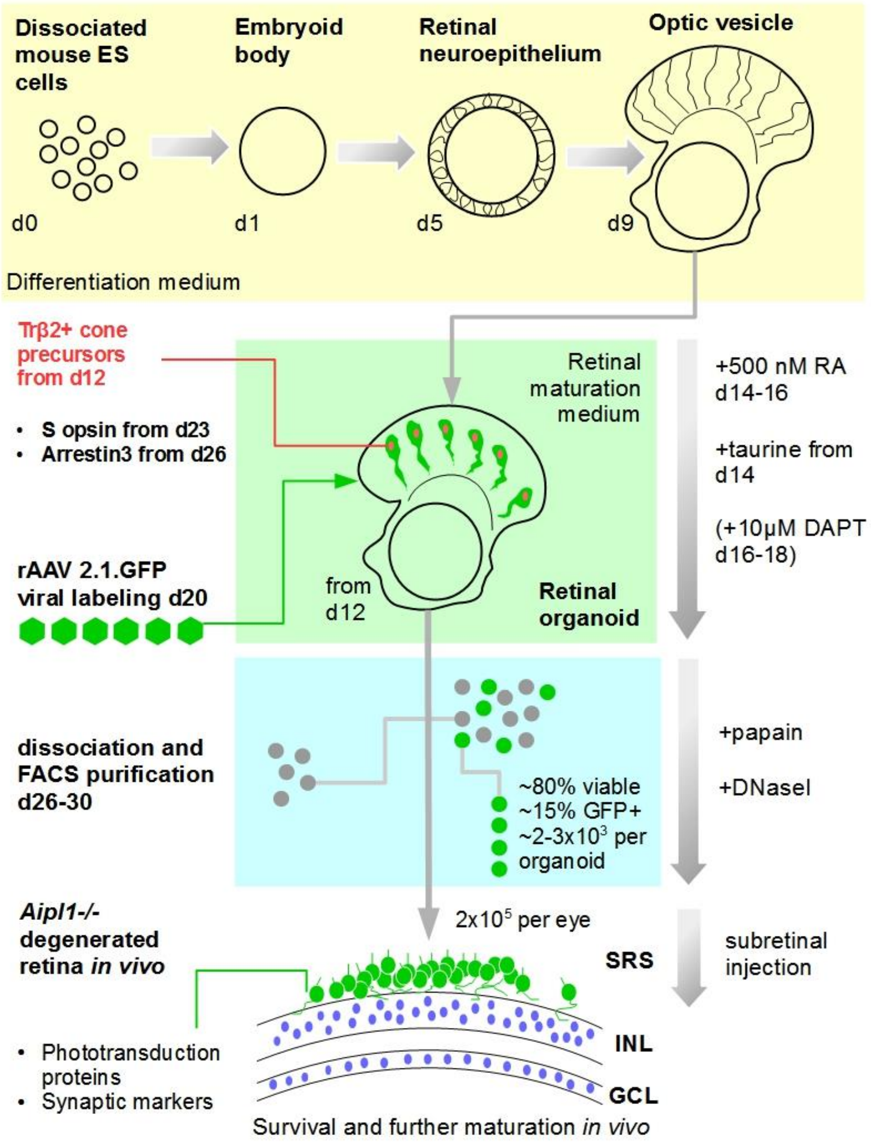

Figure S7. Summary of the protocol for differentiation, isolation and transplantation of mESC-derived cone precursors. Related to all main figures.

Maintenance cultures of mESCs were dissociated and $3x10^3$ cells were plated per well of a 96-well plate to form an embryoid body. Matrigel basement membrane matrix was added next day to stimulate formation of rigid continuous neuroepithelia. Cells were further cultured in retinal differentiation medium (*Differentiation medium*). Retinal neuroepithelia formed around d5 and optic vesicles developed by d9. From d12 the organoids were transferred into retinal maturation medium (*RMM*). At d12 the first cone precursors were detected by immunolabelling for Trβ2. From d14 to 16 a short pulse supplementation of 500 nM RA was performed. Taurine was added from day d14 onwards. Pharmacological inhibition of Notch signalling using 10 µM DAPT at d16 increased the proportion of differentiating cone precursors, without raising the absolute cone precursor cell yield. From d23 S opsin became expressed, whereas Arrestin3 was detected from d26. Labelling of cone precursors for transplantation was performed by transduction with rAAV vector encoding a 2.1 kb human red-green cone opsin promoter fragment driving GFP at d20. For transplantation, performed at d26-d30, organoids were dissociated using a papain-based kit with addition of DNaseI. GFP+ cells were purified from the cell suspension by FACS. Usual cell viability following dissociation was around 80%. Around 15% of cells were GFP+ and $2\text{-}3x10^3$ cone precursors were recovered from a single organoid. $2x10^5$ live cells (approximately equivalent to the number of cones in a wildtype mouse retina) were transplanted via subretinal injection into eyes of severely degenerate *Aipl1*$^{-/-}$ recipients from 8 weeks of age. Histological analysis 3 weeks post-transplantation showed expression of phototransduction-related and synaptic proteins suggesting a degree of maturation *in vivo*.

Supplemental Tables.

Table S1.

List of constructs used in the study.

Plasmid name	***Promoter/s***	***Gene/s of interest***	***Used for***
AAV2/9 cap	ITR	AAV2/9 rep cap	AAV vector production
ShH10 cap	ITR	ShH10 rep cap	AAV vector production
PHGTI	P5	E4, E2a, VA	AAV vector production
pD10.2.1.GFP	2.1*p*	GFP	AAV vector production
pD10.U6.shControl.CBA.RFP	U6, CBA	shControl, RFP	AAV vector production
pD10.U6.shNrl.CBA.RFP	U6, CBA	shNrl, RFP	AAV vector production
pD10.U6.shNr2e3.CBA.RFP	U6, CBA	shNr2e3, RFP	AAV vector production
pD10.Rhop.RFP	Rho*p*	RFP	AAV vector production

Table S2.

Gene-specific PCR primers.

Gene-specific primer details

**Gene name**	Forward Primer (5’-3’)	Reverse Primer (5’-3’)	Amplicon size (bp)	Probe number
Actb	aaggccaaccgtgaaaagat	gtggtacgaccagaggcatac	100	56
Arr3	tgtgtttgttcaggagttcaca	aggccctgcttctgacagt	105	71
Cnga3	agaacagaagccaccagacg	cagtcagccagcggtagtaga	92	67
Cngb3	tggacaaagcatatgtctacagagt	cccagtaataaacacaggcgtta	87	02
Crx	ccccaatgtggacctgat	ggctcctggtgaatgtggt	89	64
Cyp26a1	ccggcttcaggctacaga	ggagctctgttgacgattgtt	125	17
Hes1	gccagctgatataatggagaaaa	ctccatgataggctttgatgact	127	83
Hes5	ccaaggagaaaaaccgactg	cttggagttgggctggtg	128	67
Gnat1	agagctggagaagaagctgaaa	tagtgctcttcccggattca	95	89
Nrl	ttctggttctgacagtgactacg	tgggactgagcagagagagg	77	53
Nr2e3	cagccagcctgtgaggtt	agaagctcaatgcgctcag	81	32
Oc1	ggagttccagcgcatgtc	cgacgttggacgtctgtg	123	64
Olig2	agaccgagccaacaccag	aagctctcgaatgatccttcttt	107	21
Opn1sw	ccccatcatctactgcttcat	gacacgtcagattcgtctgc	93	04
Opn1mw	atcgtgctctgctacctcca	tttctgttgctttgccactg	60	05
Pde6c	tgactgcctgtgacctgtct	ttgcaaccagaagtgctacct	72	09
Raldh1	caccatggatgcttcagaga	actttcccaccattgagtgc	108	40
Raldh3	tttggtggcttcaaaatgtct	acttctgtatattcagccagagca	71	53
Rara	ggagcttggaacctgcac	gaggatgccactcccaga	76	83
Rcvrn	caatgggaccatcagcaaa	cctcaggcttgatcattttga	71	67
Rho	acctggatcatggcgttg	tgccctcagggatgtacc	70	32
Rorb	gctgactgaggaagagattgc	atcagccaggctcggtct	69	82
Rxrg	cagaagtgcctggtcatgg	cctcactctctgctcgctct	82	82
Thrb2	atgcatctatgttggcatgg	gcttggctagcctcttgct	62	42

Table S3.

Details of antibodies used for immunostaining.

Antigen	Host species	Dilution	Supplier
**Cone arrestin**	rabbit	1 in 250-500	Millipore (AB15282)
**Crx**	rabbit	1 in 500	Gift from C.Gregory-Evans
**GFP**	rabbit	1 in 300	Life Technologies (A-21311)
**GFP**	goat	1 in 300	(FITC-conj.) Abcam ab6662
**Gnat2 (Gαt2)**	rabbit	1 in 500	Santa Cruz (I-20; sc-390)
**Ki67**	rabbit	1 in 100	Abcam (ab15580)
**M opsin**	rabbit	1 in 500	Millipore (AB5405)
**LaminB**	goat	1 in 500	Santa Cruz (C-20; sc-6216)
**Olig2**	rabbit	1 in 100	Millipore (AB9610)
**Onecut1**	rabbit	1 in 100	Santa Cruz (H-100; sc-13050)
**Otx1/2**	rabbit	1 in 200	Abcam (ab21990)
**Peripherin2**	rabbit	1 in 500	Gift from G.Evans
**RALDH1 (ALDH1A1)**	rabbit	1 in 200	Abcam (ab23375)
**RARα**	rabbit	1 in 100	Santa Cruz (C-20; sc-551)
**Recoverin**	rabbit	1 in 500	Chemicon (AB5585)
**Rhodopsin**	mouse	1 in 1000	Sigma (O4886)
**Ribeye (CtBP2)**	mouse	1 in 500	BD Biosciences (612044)
**RXRγ**	rabbit	1 in 100	Santa Cruz (Y-20; sc-555)
**RXRγ**	rabbit	1 in 100	Abcam (ab15518)
**S opsin**	goat	1 in 100	Santa Cruz (H-17; sc-14365)
**S opsin**	rabbit	1 in 250-500	Millipore (AB5407)
**Synaptophysin**	mouse	1 in 1000	Sigma-Aldrich (S5768)
**Trβ2**	rabbit	1 in 500	Wako (016-24261)

Table S4.

Retinal differentiation media.

Maintenance medium	Retinal differentiation medium	Retinal maturation medium
510 ml GMEM<br>5.1 ml Non-essential amino acids<br>5.1 ml Pyruvate (1 mM)<br>0.51 ml 2-Mercaptoethanol<br>58 ml KSR (10%)<br>5.8 ml Heat-inactivated FBS (1%)	510 mls GMEM (Gibco, 21710-082)<br>5.1 mls Non-essential amino acids (Gibco, 11140-035) (0.1mM)<br>5.1 mls Pyruvate (Sigma, S8636) (1mM)<br>0.51 mls 2-ME working solution (0.1mM)<br>8.4 mls KSR (1.5%)	500 ml DMEM/F-12 GlutaMAX<br>5 ml N2 supplement<br>2.5 ml Penicillin/Streptomycin

Supplemental Experimental Procedures

DNA constructs

DNA constructs used are listed in Supplemental Table 1.

2.1.GFP promoter construct was courtesy of J. Nathans. Bovine Rhodopsin promoter (*Rhop.RFP*) was cloned and characterized in the laboratory. Knockdown constructs targeting mouse Nrl and Nr2e3 genes with an RFP reporter were cloned and amplified as described in the following section. Plasmid encoding ShH10 capsid was kindly provided by J. Flannery.

Nrl and *Nr2e3* knockdown constructs

Artificial miRNA-like hairpins targeting the coding sequence of mouse *Nrl* and *Nr2e3* genes were designed using siDESIGN software (Dharmacon). Candidates with highest scores and lowest predicted off-target effects were synthesised. Oligonucleotides coding the miRNA cassette were ligated into U6 plasmid designed for miRNA expression. Positive colonies after transformation were checked by restriction digestion (with *Asc*I and *Sna*BI enzymes) and sequencing. Knockdown efficiency was evaluated in 293T cells by qPCR.

Nrl knockdown target sequences and the short hairpin sequences synthesised were as follows:

sh-Nrl target sequence

5'-GATTTGATGAAGTTCGAAA-3'

short hairpin cassette oligonucleotides sequences:

5'-TTTGGCGAGATTTGATGAAGTTCGAAACTGTGAAGCCACAGATGGGTTTCGAACTTCATCAAATC CTGCTTTT-3'

and

5'-CTAGAAAAGCAGGATTTGATGAAGTTCGAAACCCATCTGTGGCTTCACAGTTTCGAACTTCATCA AATCTCGC -3'

Target sequences and miRNA cassette sequences that showed substantial reduction of *Nr2e3* levels are as follows:

sh-Nr2e3 target sequence

5'-GGACAGCAGCAGTGGGAAA-3',

short hairpin cassette oligonucleotides sequences:

5'-TTTGGCGAGGACAGCAGCAGTGGGAAACTGTGAAGCCACAGATGGGTTTCCCACTGCTGCTGT CCCTGCTTTT-3'

and

5'–CTAGAAAAGCAGGGACAGCAGCAGTGGGAAACCCATCTGTGGCTTCACAGTTTCCCACTGCTG CTGTCCTCGC-3'

sequences forming the hairpin regions are underlined.

Recombinant adeno-associated viral (rAAV) vector production

Production of recombinant AAV vectors was carried out in HEK 293T cell line transfected with three plasmids encoding construct of interest, viral capsid and helper genes (on pHGTI plasmid). The plasmids were transfected using polyethyleneimine (PEI). HEK 293T cells were seeded onto 150 mm tissue culture plates (Greiner Bio-One Ltd., UK) at 10^6 cells per plate in DMEM Glutamax® (Life Technologies Ltd., UK) medium containing 10% foetal bovine serum (Life Technologies Ltd., UK) and penicillin, streptomycin and antifungal agent mix (Life

Technologies Ltd., UK). The following day cells reached approximately 70% confluency. 20 plates of cells were used for a batch of virus. Transfection mixture contained 52.5 ml DMEM, 100 µg capsid plasmid, 30 µg pHGTI helper, 100 µg gene transfer plasmid and 1.2 ml PEI. Transfection solution was incubated at room temperature for 10 minutes and then added dropwise onto plates with cells. Medium was replaced following day. 72 hr from transfection, cells were harvested by scraping off plates using a cell scraper (Greiner Bio-One Ltd., UK). Medium with cells was collected in 50 ml Falcon tubes and spun down at 2000 xg for 5 minutes and resuspended in TD buffer pH 7.4. Release of viral particles from harvested cells and adequate lysis was facilitated by 3 cycles of freezing (in -80°C), thawing (at 37°C) and mixing on a vortex shaker for 5 minutes. This was followed by treating the cell lysate with 50 units of benzonase (Sigma-Aldrich Ltd., UK). Benzonase was used to remove remaining free plasmid DNA. Cell lysate was subsequently centrifuged at 18000 xg and supernatant filtered through membrane filters, first 5 µm pores, then 0.45 µm and, finally, 0.22 µm. Viral vector was purified from this supernatant by means of ion exchange fast protein liquid chromatography (FPLC). ÄKTA™ prime or ÄKTA™ pure (GE Healthcare Ltd., UK) FPLC apparatus was used to purify vector particles on anionic sephacryl S300 and a POROS 50HQ columns prior to elution using increasing salt gradient. This yielded approximately 20 mls of eluate which was concentrated on Vivaspin 4 columns (Sartorius AG., Germany) to a final volume of 250 µl. Viral preparation was aliquoted and stored at -80°C until use. Concentration of copies of viral genome in ml of vector preparation was determined by quantitative real time PCR using primers specific for viral inverted terminal repeat (ITR). Number of copies of viral genome was determined by comparison of amplification of samples with amplification of standards of known quantity of vector.

Quantitative PCR

50 ng of cDNA was loaded per well of 96-well plate (Life Technologies Ltd., UK) mixed with 2x Fast Start TaqMan® Probe Master Mix (Roche Ltd., UK), gene-specific forward and reverse primers at a final 900 nM concentration and an appropriate hydrolysis probe binding to the amplified region at a final concentration of 250 nM (Roche Diagnostics Ltd., UK), all dissolved in DNase and RNase free water up to 20 µl final volume. Each cDNA samples was run in triplicate. The reactions were then run on an ABI Prism 7900HT Fast Real-time Sequence Detection System (Applied Biosystems Ltd., UK) equipped with SDS 2.2.2 software for amplication results analysis. From amplification curves Ct values were obtained for each sample. Expression levels were normalized to beta actin (*Actb* gene) mRNA levels for each sample to assess relative expression of particular genes in different experimental conditions. Cycling conditions were as follows 40 cycles of 95°C for 30 sec. and 60°C for 1 minute. Supplementary Table 2 contains the list of gene-specific primer sequences used.

Immunohistochemistry

Tissue from was frozen in OCT embedding matrix (Pyramid Innovation Ltd., UK) and sectioned at 18 µm on a Bright OTF 5000 cryostat (Bright Instruments Ltd., UK). Cryosections were thawed and rehydrated by adding PBS. For certain antibody staining sections were treated with 1 or 4% paraformaldehyde solution for 10 minutes followed by a wash with PBS. Slides were blocked with a solution of 5% goat or donkey serum, 1% BSA, 0.1% Triton X-100 in PBS for 2 hours at room temperature. This was followed by 5 washes with PBS, after which primary antibodies were added dissolved in 1% BSA, 0.1% Triton X-100 in PBS and incubated overnight at 4°C. List of primary antibodies used in this study can be found in Table 3. Following day slides were washed 5 times with PBS, before adding Alexa Fluor®-conjugated secondary antibodies (Life Technologies Ltd., UK) diluted 1:100-1:500 (depending on the primary antibody used) in 1% BSA, 0.1% Triton X-100 in PBS. Slides were incubated with secondary antibodies for 2 hours at room temperature. Subsequently, they were washed 3 times with PBS and nuclei counterstained with 4'-6-diamidino-2-phenylindole (DAPI; Sigma-Aldrich Ltd., UK) and mounted using coverslides and fluorescent microscopy mounting medium (DAKO Ltd., UK).

All antibodies used are listed in Supplemental Table 3.

Cell counts

Quantification of the percentage of cells expressing cone markers in retinal organoids was performed using 10 µm thick confocal stacks (acquired under 40x objective) using Cell Counter plug-in in ImageJ software. Numbers of marker-positive cells were normalized either to DAPI-stained nuclei or CrxGFP+ cells in the same images. Multiple individual organoids from at least 3 differentiation cultures were used for each quantification as specified in respective figure descriptions.

CrxGFP+/S opsin+ cells were counted on 18 µm-thick P8 CrxGFP transgenic mouse retina cryosections. Using a 40x objective, 10 µm-thick confocal Z stacks were acquired from dorsal, mid-central and ventral retina, 9 images per retina, n=3 retinae from 3 individual mice. Results were averaged for each retina analysed.

Rxrγ-positive nuclei and M, S opsin-positive photoreceptor processes were counted in 15 µm-thick confocal stacks (40x objective) and normalized to CrxGFP+ photoreceptors present in the image. A minimum of 3 images from each transplanted retinae were used, with 3 transplanted retinae examined.

Mouse ESC maintenance culture

Maintenance of undifferentiated mouse ES cells state was performed by culture in the presence of leukemia inhibitory factor (LIF, Millipore Ltd., UK) or, alternatively the '2i' medium containing CHIR99021 and PD03259010, inhibitors of GSK3β and MEK respectively. LIF was used at 1000U/ml, whilst CHIR99021 at 3 µM and PD03259010 at 1 µM. Cells were kept as adherent cultures on 0.1% gelatin-coated dishes. To avoid spontaneous differentiation, the cells were kept at low (below 30%) confluence and passaged every other day. Cells were dissociated for 5 minutes at 37°C using 0.25% trypsin-EDTA solution (Life Technologies Ltd., UK). Viability was assessed using trypan blue dye. Concentration of cells was determined with a haemocytometer and 1.5 x 10^5 cells were added per 60 mm plate, for the larger 100 mm plate 4.2 x 10^5 cells were plated in maintenance medium containing 1000U/ml of LIF. Cells were cultured at 37°C in 5% CO_2.

Retinal differentiation

Day 0. Maintenance cultures were dissociated using 0.25% trypsin-EDTA solution. Cells were subsequently plated into Nunclon Sphera ultra low-binding 96-well plates (Thermo-Fisher Scientific Ltd, UK). Total of 3.0 x 10^5 cells was resuspended in 10 ml of differentiation medium, mixed through pipetting and 100 µl of suspension added per well of the 96-well plate, so that an average of 3000 cells was plated into each well subsequently aggregating to form an embryoid body.

Day 1. 24 hours after plating the aggregates were examined under a light microscope to assess embryoid body formation followed by addition of matrigel solution to trigger continuous retinal neuroepithelia formation. Matrigel solution was prepared by rapidly dissolving 1 ml of growth factor reduced (GFR) Matrigel working solution (1:35 dilution) in 4 ml of differentiation medium per 96-well plate. Next, 50 µl of this freshly prepared Matrigel solution was added per well to give a final concentration of 2% Matrigel. Following Matrigel addition aggregates were cultured for another 8 days at 37°C in 5% CO_2.

Day 9. Organoids were transferred into a low-binding 24-well tissue culture plate (Thermo-Fisher Scientific Ltd, UK) using wide bore pipette tips (VWR Ltd, UK), 12 aggregates per single well of a 24-well plate. Differentiation medium carried over with the embryoid bodies was gently aspirated using a 1 ml pipette and replaced with 1 ml of pre-warmed retinal maturation medium (RMM).

Day 14. 5 µl of retinoic acid working solution was added per ml of RMM (500 nM final concentration) and 3 µl per ml of taurine working solution (150 nM final concentration).

Media components are listed in Supplemental Table 4.

Treatment with with Notch inhibitors DAPT and RO4929097

Organoids at either day 16 or 21 of culture had their media replaced with pre-warmed RMM supplemented with 150 nM taurine and containing DMSO as a vehicle control or 10 µM final concentration of DAPT (Sigma-Aldrich Ltd, UK) or RO4929097 (BioVision Inc., USA; 1 µl of 10 mM working solution and 10 µl of 1 mM working solution, respectively). Cells were next incubated with inhibitors for 48 hours before processing for analysis by flow cytometry or collection for quantitative PCR.

Dissociation of retinal organoids

Papain-based Miltenyi Biotec Neural Tissue Dissociation Kit (Miltenyi Biotec GmbH. Germany) was used according to manufacturer's recommendations. Briefly, solution 2 from the kit was pre-warmed for 15 minutes in a water bath set at 37°C, 960 µl was used per 70-100 aggregates. Embryoid bodies were collected with wide-bore pipette tips into 15 ml Falcon tubes (aggregates from 6-8 wells into a single tube). Embryoid bodies were left for several minutes to sink down to the conical bottom of the tube. RMM medium was removed from above the aggregates and they were washed with 10 ml of PBS (without Ca^{2+} and Mg^{2+} ions; Thermo-Fisher Scientific Ltd, UK). PBS was aspirated and organoids suspended in pre-heated solution 2. To each tube 25 µl of solution 1, 10

µl of solution 3 and 5 µl of solution of 4 was added and mixed with the organoids in suspension. The falcon tubes were then incubated at 37°C in a water bath for 15-25 minutes, mixed every 3-5 minutes. Following the incubation, organoids were mechanically dissociated by gentle trituration with a P1000 pipette. Partially dissociated samples were then incubated at 37°C for another 5 minutes and again triturated. Tubes were then centrifuged at 1000 rpm for 7 minutes. Supernatant was aspirated and cells resuspended in 1 ml of resuspension solution composed of 66% Eagle's Minimum Essential Medium (MEM-E HEPES), 33% Hank's Balanced Salt Solution (HBSS), 1% heat-inactivated FBS (all Thermo-Fisher Scientific Ltd, UK) and supplemented with 20 units/ml of DNase I (Sigma-Aldrich Ltd, UK). Once homogenous, the solution was passed through a 40µm cell strainer (BD Biosciences Ltd, UK).

Fluorescence-activated cell sorting

Cell sorting was performed either using BD Influx Cell Sorter (Beckman Dickinson Inc., USA) or MoFlo XDP (Beckman Coulter Inc., USA). Both sorters were fitted with a 200 mW 488 nm blue laser (adjusted to 150 mW for sorting) that was used to excite GFP or RFP with the GFP signal detected in 530/40 nm channel, whilst RFP signal in 613/20 nm channel. Sorting of photoreceptor cells was performed at 50 psi and a 70 nozzle was used. Flow-sorted mESC-derived GFP+ cells were on average >95% pure GFP-positive, and >80% viable.

Flow cytometry analysis

DRAQ7 (Biostatus, DR71000) dead cell stain was added to the samples at a final concentration of 0.3µM for 5 minutes at room temperature, followed by sample acquisition. All of the samples were analysed using a BD LSRFortessa X-20 flow cytometer (Beckman Dickinson Inc., USA), fitted with 5 lasers (i.e. 355nm, 405nm, 488nnm, 561nm & 640nm lasers). Results were subsequently analysed using FlowJo software.

Viability and cell yields assessment

Cell viability and number in dissociated samples was determined using Vi-Cell^{TM} XR cell viability analyser. Live/dead discrimination was performed using trypan blue dye. Cell yields were calculated by combining cell number and viability with flow cytometry results. In each experiment the results were normalised to either untreated or DMSO control conditions (given value of 100) to assess treatment effect. Three independent experiments with at least 36 organoids each were performed.

Animal models

Care of all animals used in this study was according to standards described in the Animal (Scientific Procedures) Act 1986. All the procedures were performed in accordance with the Association for Research in Vision and Ophthalmology (ARVO) Statement on the Use of Animals in Ophthalmic and Vision Research. Transplantation of photoreceptor precursors was performed at 8-16 weeks of age in both wild type and *Aipl1*$^{-/-}$ animals. Animals used were housed in conditions of standard 12 hour light-darkness cycle with food and water *ad libitum*.

Anaesthesia

Animals were anaesthetised by intraperitoneal injections of an anaesthetic solution composed of Dormitor (1 mg/ml, Pfizer Pharmaceuticals Ltd, UK) and ketamine (100 mg/ml, Fort Dodge Animals Health Ltd, UK) mixed with sterile water for injections (Thermo-Fisher Scientific Ltd, UK) in a ratio of 5:3:42. Young adult animals that were used for subretinal injections weighing approximately 200g received 0.2 ml of anaesthetic solution. Once anaesthetised, a small drop of Viscotears (Alcon Ltd, UK) was used topically on the cornea to prevent it from drying during the procedure. After the injections, in order to reverse the effects of the anaesthetic, 0.2 ml of Antisedan (0.1 mg/ml, Pfizer Pharmaceuticals Ltd, UK) was administered through intraperitoneal injection and the mice were placed on a heat mat until they regained normal mobility.

Subretinal transplantation

Cells sorted into 15 ml Falcon tubes were centrifuged for 10 minutes at 1000 rpm and resuspended in HBSS (+Ca^{2+}, +Mg^{2+}) supplemented with DNase I (50 units/ml). Viability was determined using trypan blue staining, cells were counted using Neubauer haemocytometer and resuspended to a final of 10^5 cells per µl. Before surgery, pupils of the animals were dilated by topical administration of 1% Tropicamide solution (Chauvin Pharmaceuticals Ltd, UK). Surgery was performed under direct retinoscopy utilising an operating microscope (Leica AG, Germany). In preparation for injections, the eye was protruded forward by application of small

pressure on both sides of the eye and kept in place by holding a section of conjunctiva and extraocular muscle using a pair of forceps. By placing a coverslip on the cornea covered in Viscotears solution (Alcon Ltd, UK) acting as coupling medium, a contact lens system was created enabling visualisation of the fundus. Injections were performed with a 1.5 cm, 34-gauge hypodermic needle mounted on a 5 µl Hamilton syringe (Thermo-Fisher Scientific Ltd, UK). Tip of the syringe was placed underneath the coverslip and then guided to the sclera and then inserted tangentially through it creating a wound tunnel that self-seals. Once the needle was inserted its tip was brought into focus between the retina and the RPE and 1 µl of cell suspension containing 10^5 cells selected for GFP expression was injected. This procedure was performed for the superior and inferior hemispheres of the eye.
