# Supplementary material for: Differentiation and Transplantation of Embryonic Stem Cell-Derived Cone Photoreceptors into a Mouse Model of End-Stage Retinal Degeneration
Source: Stem Cell Reports. 2017 May 25;8(6):1659–74. doi: 10.1016/j.stemcr.2017.04.030 (PMC5470175; doi:10.1016/j.stemcr.2017.04.030)
Supplement: Document S2. Article plus Supplemental Information [file mmc2.pdf]

Stem Cell Reports
Article
OPEN ACCESS

Differentiation and Transplantation of Embryonic Stem Cell-Derived Cone Photoreceptors into a Mouse Model of End-Stage Retinal Degeneration

Kamil Kruczek,[1] Anai Gonzalez-Cordero,[1] Debbie Goh,[1] Arifa Naeem,[1] Mindaugas Jonikas,[1] Samuel J.I. Blackford,[1] Magdalena Kloc,[1] Yanai Duran,[1] Anastasios Georgiadis,[1] Robert D. Sampson,[1] Ryea N. Maswood,[1] Alexander J. Smith,[1] Sarah Decembrini,[2] Yvan Arsenijevic,[2] Jane C. Sowden,[3] Rachael A. Pearson,[1] Emma L. West,[1] and Robin R. Ali[1,4,*]
[1]Department of Genetics, UCL Institute of Ophthalmology, London EC1V 9EL, UK
[2]Department of Ophthalmology, Jules-Gonin Eye Hospital, University of Lausanne, 1004 Lausanne, Switzerland
[3]Stem Cells and Regenerative Medicine Section, UCL Great Ormond Street Institute of Child Health, University College London, London WC1N 1EH, UK
[4]NIHR Biomedical Research Centre, Moorfields Eye Hospital NHS Foundation Trust, City Road, London EC1V 2PD, UK
*Correspondence: r.ali@ucl.ac.uk
http://dx.doi.org/10.1016/j.stemcr.2017.04.030

SUMMARY

The loss of cone photoreceptors that mediate daylight vision represents a leading cause of blindness, for which cell replacement by transplantation offers a promising treatment strategy. Here, we characterize cone differentiation in retinas derived from mouse embryonic stem cells (mESCs). Similar to in vivo development, a temporal pattern of progenitor marker expression is followed by the differentiation of early thyroid hormone receptor β2-positive precursors and, subsequently, photoreceptors exhibiting cone-specific phototransduction-related proteins. We establish that stage-specific inhibition of the Notch pathway increases cone cell differentiation, while retinoic acid signaling regulates cone maturation, comparable with their actions in vivo. MESC-derived cones can be isolated in large numbers and transplanted into adult mouse eyes, showing capacity to survive and mature in the subretinal space of *Aipl1*$^{-/-}$ mice, a model of end-stage retinal degeneration. Together, this work identifies a robust, renewable cell source for cone replacement by purified cell suspension transplantation.

INTRODUCTION

Cone photoreceptors mediate high acuity and color vision in daylight. Cones are a rare subtype in most mammals, constituting approximately 3% of photoreceptors in mice and 5% in humans (Jeon et al., 1998). Due to scarcity, our understanding of their development and physiology is less advanced than that of rods, the major photoreceptor type responsible for night vision (Swaroop et al., 2010). A common feature of both inherited and age-related retinal degeneration is the death of photoreceptors. Loss of cones has a far greater effect on vision in patients, and replacement of small numbers of cones may result in substantial benefits (Jayakody et al., 2015). Interest in developing methods of photoreceptor differentiation from stem cells is driven by the potential to establish cell-based therapies to treat retinal degenerations (Jayakody et al., 2015), as well as providing in vitro models both to study disease mechanisms and for drug discovery. Two main approaches for photoreceptor replacement have been developed in preclinical research. In the first, a whole neural retina sheet is placed into the diseased environment (Seiler and Aramant, 2012). The second is transplantation of cells in suspension (Jayakody et al., 2015). Retinal sheet transplantation has the advantage of supplying a structured tissue, although injection of a flat, thick sheet of cells with the correct polarity into the subretinal space is surgically challenging (Reh, 2016; Seiler and Aramant, 2012). Moreover, inclusion of interneurons limits the connectivity of graft photoreceptors to host inner retinal circuitry (Assawachananont et al., 2014; Reh, 2016; Shirai et al., 2016). By contrast, dissociated photoreceptors can be purified to remove other cell types that may prevent them from forming synaptic contacts with the host interneurons (Singh et al., 2013). Postmitotic photoreceptor precursors isolated from donor mice injected subretinally as a suspension are capable of driving visual function in models of retinal degeneration (Barnea-Cramer et al., 2016; MacLaren et al., 2006; Pearson et al., 2012; Santos-Ferreira et al., 2015; Singh et al., 2013).

Despite the importance of cones for human vision, few studies have looked at the possibility of transplanting isolated donor-derived cones (Lakowski et al., 2010; Santos-Ferreira et al., 2015; Smiley et al., 2016). We have reported transplantation of a mixed population of embryonic cone rod homeobox-positive (CRX^{+}) photoreceptors, enriched for cones compared with postnatal stages (Lakowski et al., 2010), while Ader and colleagues used cone-like cells from mice lacking NRL (neural retina leucine), a transcription factor essential for rod genesis, reporting an improvement in light-evoked responses in mice lacking cone function (Santos-Ferreira et al., 2015). In a recent proof-of-concept study, cones purified using a novel reporter mouse line were transplanted into wild-type retina (Smiley et al., 2016). However, the cones observed in the host photoreceptor layer resembled rods morphologically and have subsequently been shown to result from uptake of

This is an open access article under the CC BY license (http://creativecommons.org/licenses/by/4.0/).

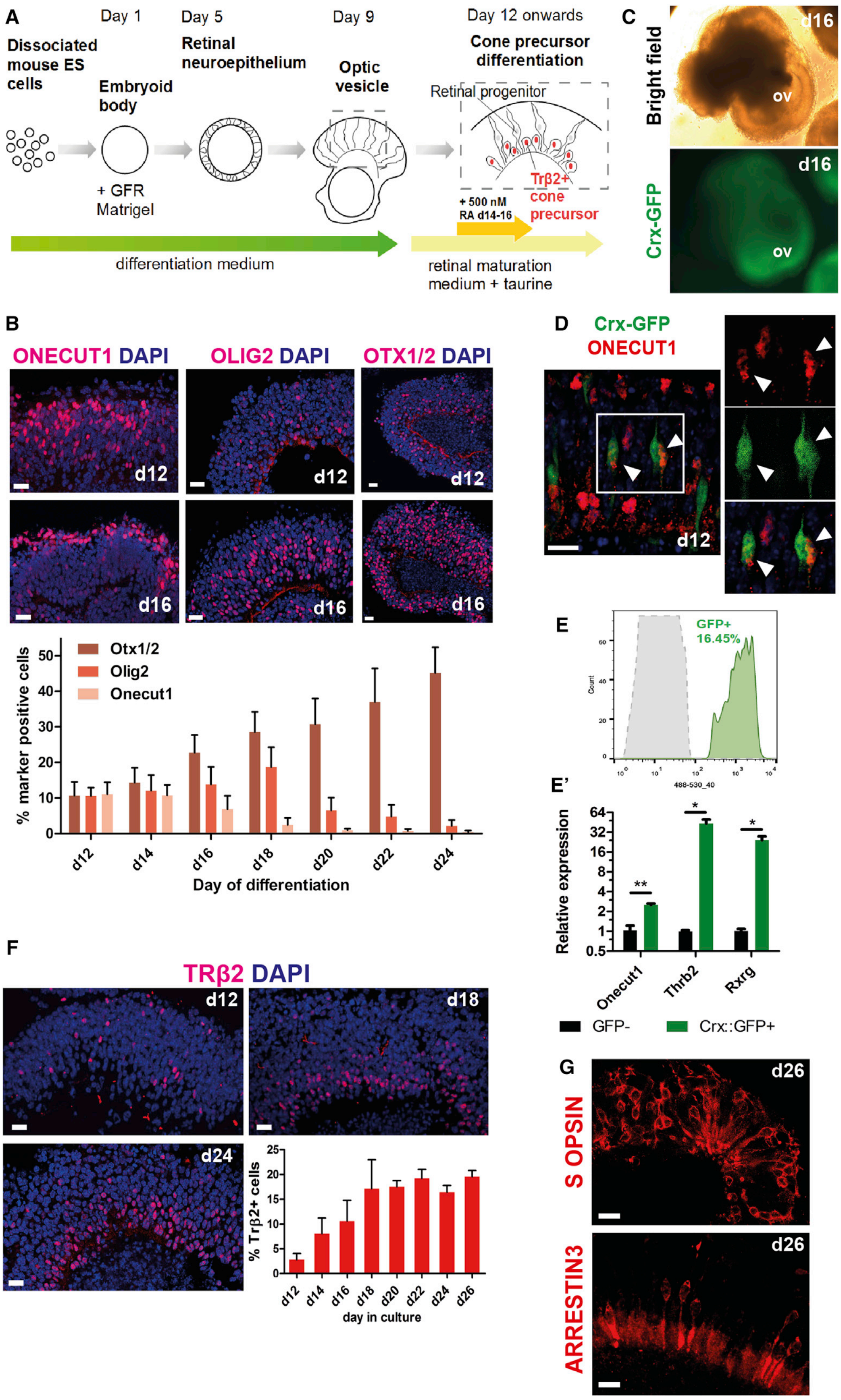

(legend on next page)

cytoplasmic components by host photoreceptors from the graft (Decembrini et al., 2017; Ortin-Martinez et al., 2016; Pearson et al., 2016). Therefore, the true morphology and maturation characteristics of transplanted cones remain largely unknown. Notably, none of the previous studies examined transplantation into a model exhibiting extensive photoreceptor loss, a feature observed in patients with advanced retinal disease that would benefit most from photoreceptor transplantation (Reh, 2016).

Another limitation of these earlier studies is the use of primary donor-derived cells, while successful clinical translation of these findings will require a renewable, efficient, and scalable cell source. Advances in retinal differentiation using mouse and human pluripotent stem cells (PSCs) (Eiraku et al., 2011; Gonzalez-Cordero et al., 2013; Meyer et al., 2009; Nakano et al., 2012; Zhong et al., 2014), including the generation of optic cups in 3D floating culture of mESCs, enabled the transplantation of ESC- and induced PSC-derived retinal sheets (Assawachananont et al., 2014; Shirai et al., 2016) as well as suspensions of purified rod precursors (Decembrini et al., 2014; Gonzalez-Cordero et al., 2013). However, cone differentiation in these cultures has not been characterized, nor has their transplantation potential been assessed.

In early stages of commitment to the cone lineage, co-expression of transcription factors ONECUT1 and OTX2 in retinal progenitors positive for OLIG2 leads to transcriptional activation of thyroid hormone receptor β2 (TRβ2) (Emerson et al., 2013), an early cone marker (Ng et al., 2009). An important negative regulator of cone and rod genesis is the Notch pathway, acting to promote non-photoreceptor fates (Mizeracka et al., 2013). Little is known about the soluble signals cooperating with intrinsic determinants to regulate cone differentiation. In zebrafish, application of exogenous retinoic acid (RA) results in precocious rod differentiation, while the accompanying cones retain immature morphology (Hyatt et al., 1996). Similarly, RA facilitates rod gene expression in mESC-derived retinal organoids (Gonzalez-Cordero et al., 2013). Nevertheless, its effects on mammalian cone differentiation remain undetermined.

In this work, we characterize formation of mESC-derived cones and establish that in vitro the Notch pathway and RA signaling regulate their differentiation and maturation, respectively. Furthermore, we demonstrate that mESC-derived retinas generate a significant proportion of cone precursors. This permitted the isolation of large numbers of purified cones for transplantation into the *Aipl1*$^{-/-}$ mouse model of end-stage degeneration, in which nearly all host photoreceptors are lost by postnatal day 30 (P30) (Ramamurthy et al., 2004). In this environment, transplanted mESC-derived cone photoreceptors show survival and maturation features that cannot result from cytoplasmic material transfer. Together, we provide a proof of concept for cone cell replacement via purified cell suspension transplantation.

RESULTS

Recapitulation of Stepwise Commitment to the Cone Lineage in mESC-Derived Retinas

To examine cone differentiation from mESCs, we adapted an established protocol for the generation of retinal organoids recapitulating early retinal histogenesis (Figures 1A and S1A–S1D; Decembrini et al., 2014; Eiraku et al., 2011; Gonzalez-Cordero et al., 2013). In vivo, a subpopulation of retinal progenitors biased toward cone genesis is marked by co-expression of the transcription factors ONECUT1, OTX2, and OLIG2 (Emerson et al., 2013; Hafler et al., 2012). Cone genesis is completed before birth in murine retina (Carter-Dawson and LaVail, 1979). On day 12 (d12) to d18 in culture, which corresponds to between embryonic day 12 (E12) and E18 in vivo (see Figure 4E for comparison with in vivo development [Decembrini et al., 2014; Eiraku et al., 2011; Gonzalez-Cordero et al., 2013; Swaroop et al., 2010]), gene expression analysis (Figure S1E) and immunohistochemistry (Figure 1B) showed expression of

Figure 1. Sequential Commitment to the Cone Photoreceptor Lineage Is Recapitulated In Vitro in mESC-Derived Retinas

(A) Schematic depiction of the differentiation protocol used in the study.

(B) Expression of ONECUT1, OLIG2, and OTX2 determined by immunostaining. d, day. Scale bar, 20 μm. Quantification: for each time point n > 10 images of neural retinal regions from different organoids, N = 3 differentiation cultures. Mean ± SD.

(C) Crx-GFP retinal organoids showing expression of the fluorescent reporter. ov, optic vesicle.

(D) Co-staining of Crx-GFP$^+$ photoreceptor precursors with ONECUT1 (arrowheads). Scale bar, 10 μm.

(E) Flow-cytometry histogram showing GFP reporter expression in dissociated Crx-GFP line aggregates at day 16 of differentiation.

(E′) qPCR analysis of *Onecut1*, *Thrb2*, and *Rxrg* expression in flow-sorted Crx-GFP$^+$ versus GFP$^-$ populations. N = 3, Mean ± SD. *p < 0.05, **p < 0.01, Student's t test.

(F) Immunostaining for TRβ2 in mESC retinal organoids at days 12, 18, and 24 of differentiation. Quantification showing percentage of positive nuclei at indicated time points. n > 10 neural retina regions in individual organoids from N = 3 differentiation cultures quantified for each time point. Mean ± SD. Scale bar, 20 μm.

(G) Antibody staining for S OPSIN and ARRESTIN3 at day 26 in culture. Scale bar, 10 μm.

ONECUT1, OTX2, and OLIG2 in retinal organoids. Quantification of the number of cells expressing these proteins in the neural retina-like regions of the organoids revealed a dynamic temporal pattern. The percentage of ONECUT1$^+$ cone and horizontal cell progenitors decreased markedly between equivalent of embryonic (d12, 11% ± 3%) and neonatal (d20, 1% ± 0.5%) stages (n > 10 images of individual organoids for each time point; N = 3 differentiation cultures). Conversely, the percentage of OTX2$^+$ cells, which marks all photoreceptor precursors together with bipolar cells (Nishida et al., 2003), continued to rise (11% ± 4% on d12 versus 31% ± 7% on d20). OLIG2 was most widely expressed at d18 (19% ± 6%), correlating with the peak of rod birth in these cultures (Eiraku et al., 2011) and consistent with its expression in progenitors giving rise to both rods and cones (Hafler et al., 2012). Since ONECUT1 is initially expressed both in cones and horizontal cells, we sought to examine its expression in the early photoreceptor precursor population. We utilized organoids derived from the previously characterized Crx-GFP reporter mESC line (Decembrini et al., 2014; Figure 1C), in which GFP localizes to developing photoreceptor precursors. Immunostaining for ONECUT1 only showed co-localization in a small subpopulation of Crx-GFP$^+$ cells at d12 of differentiation (Figure 1D) and was no longer detectable at d20 (Figure S1F), consistent with its transient expression in developing cones in vivo (Emerson et al., 2013). As predicted, in the neural retina which constitutes most of the organoid tissue, OTX2 staining overlapped significantly with the GFP signal (shown at d24 in Figure S1G). Together, these observations suggest that the temporal appearance of markers of progenitor competence for cone genesis is largely recapitulated in vitro.

In early postmitotic cone precursors, OTX2 and ONECUT1 bind to regulatory sequences in the *Thrb2* gene locus to stimulate its transcription (Emerson et al., 2013). These cells are also characterized by expression of retinoid receptor gene *Rxrg* (Roberts et al., 2005). To compare gene expression in photoreceptors from retinal organoids with previously reported data for the parental Crx-GFP transgenic mouse line at E16 (Muranishi et al., 2010), we purified GFP$^+$ cells at d16 (Figure 1E) by fluorescence-activated cell sorting (FACS). Consistent with the expression pattern in E16 Crx-GFP$^+$ mouse cells, Crx-GFP$^+$ photoreceptors from d16 retinal organoids were significantly enriched for *Oc1* transcripts (2.5-fold over GFP$^-$ cells), *Thrb2* and *Rxrg* (43- and 23-fold, respectively; N = 3, *p < 0.05, **p < 0.01; unpaired t test Figure 1E′). We next examined the appearance of proteins specific to developing cones. Immunostaining identified TRβ2 protein in the neural retina from d12, with the number of positive nuclei increasing until around d20 (3% ± 1.3% on d12 versus 18% ± 4.0% on d20, n > 10, N = 3; Figure 1F) in a pattern resembling in vivo retina (Ng et al., 2009). These cone precursors were present at least until d26 (~P6 in vivo) in large numbers (20% ± 6%, n = 23, N = 3). Detection of the visual pigment protein S OPSIN and the phototransduction protein ARRESTIN3 suggested onset of maturation around d26 (Figure 1G). These data indicate sequential commitment to the cone lineage in our differentiation protocol equivalent to embryonic and early postnatal development.

Stage-Specific Cone Genesis In Vitro Is Limited by Notch Signaling

In the developing mouse retina, cone precursor birth is confined to the early phase of retinogenesis, between E11 and E19 (Carter-Dawson and LaVail, 1979). The number of progenitors exiting the cell cycle to give rise to cones is limited by the activity of NOTCH1 receptor, which maintains a sufficient pool of progenitors for the generation of other retinal neurons (Jadhav, 2006; Nelson et al., 2007; Yaron, 2006). Notch inhibition during the period of cone genesis should therefore raise the proportion of cone precursors. To determine whether this Notch-regulated program of neurogenesis is recapitulated in vitro, we treated the cultures with the Notch signaling inhibitor DAPT ((S)-tert-butyl 2-((S)-2-(2-(3,5-difluorophenyl)acetamido) propanamido)-2-phenylacetate) on days 16–18 of differentiation (~E16–E18 in vivo, when cone genesis is still incomplete [Carter-Dawson and LaVail, 1979]). Treatment with 10 μM DAPT resulted in the loss of dividing progenitors (determined by a reduction in Ki67 and SOX9 staining, Figures S2A and S2B), including a reduction in OLIG2$^+$ progenitor cells, which give rise predominantly to cones and rods (Figure 2A). These changes correlated with an increase in the proportion of postmitotic Crx-GFP$^+$ photoreceptor precursors, detected by both GFP immunohistochemistry (Figures 2A and S2C) and flow cytometry on dissociated cultures (Figure S2D, N = 5), without affecting cell viability, but leading to some reduction in cell yields at d26 (Figures S3A–S3C, N = 3). qPCR analysis of gene expression in DAPT-treated cultures showed a significant reduction in the expression of the Notch target gene *Hes5* (6.7-fold, n = 5 RNA samples, 12 organoids each, N = 3, p < 0.0001, unpaired Student's t test) and an upregulation of cone and rod photoreceptor-specific genes, including *Crx*, *Opn1sw*, *Nrl*, and *Rho* (p < 0.05), compared with control cultures (Figure 2B). Immunohistochemistry showed increased immunoreactivity for TRβ2 (Figure 2C). Furthermore, the proportion of Crx-GFP$^+$ cells co-expressing the cone visual pigment S OPSIN, which in control cultures was very similar to the early postnatal mouse retina (8% ± 7% at d26 versus 7% ± 2% at P8 in vivo; Figures S3D and S3E), was substantially increased (16% ± 7%, n > 30, N = 2; Figures 2D and S3E). The abundance of early born horizontal cells (CALBINBIN$^+$) remained unchanged

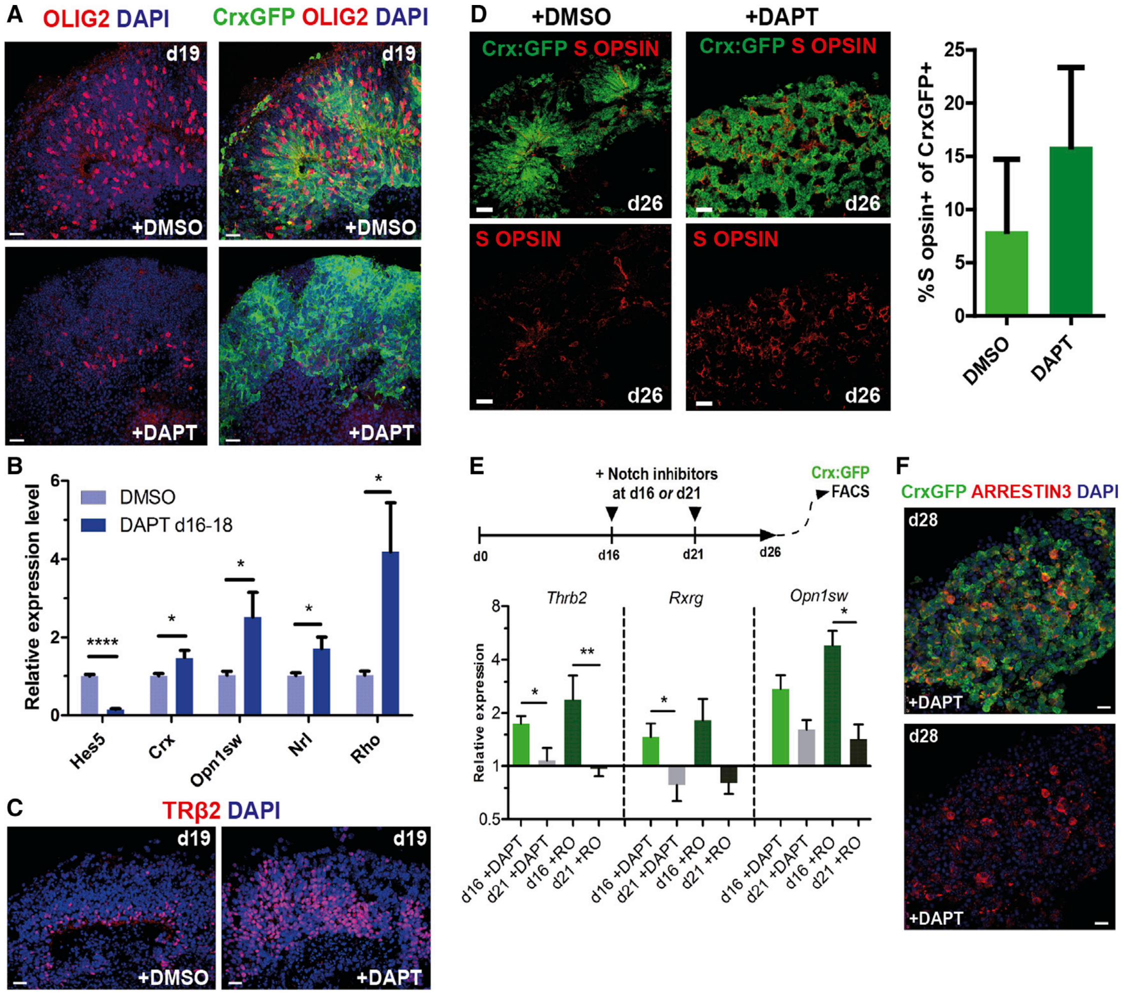

Figure 2. Notch Signaling Limits Developmental Stage-Restricted Cone Genesis

(A) Fluorescent microscopy showing staining for OLIG2 and GFP reporter in control Crx-GFP retinal organoids (upper panel) and treated with 10 μM DAPT at days 16–18. Note the loss of OLIG2 staining following DAPT treatment.

(B) qPCR gene expression analysis of control and DAPT-treated organoids at day 22, n = 5. Mean ± SD. *$p < 0.05$, ****$p < 0.0001$, Student's t test.

(C) Antibody staining for TRβ2 in control and DAPT-treated organoids. Note the increase in TRβ2$^+$ nuclei.

(D) S OPSIN immunostaining in control versus DAPT-treated Crx-GFP organoids. Quantification of the proportion of Crx-GFP$^+$ cells co-expressing S OPSIN. N = 2, n > 30. Mean ± SD.

(E) qPCR gene expression analysis of RNA samples from organoids treated with 10 μM DAPT or 10 μM RO4929097 at day 16 or day 21 of differentiation. Expression normalized to DMSO control cultures. N = 6. Mean ± SD. *$p < 0.05$, **$p < 0.01$, Student's t test.

(F) Fluorescent microscopy showing expression of the Crx-GFP reporter and ARRESTIN3 at day 28 in an organoid treated with DAPT at day 16 of culture.

All scale bars, 10 μm.

(Figures S3F and S3G); however, late progenitors/Müller glia (SOX9$^+$) were significantly depleted (14 ± 2 versus 7 ± 1 per 10^4 μm^2 of organoid area; n = 26, N = 3, p = 0.0002, unpaired t test; Figures S3H and S3I). Together, this suggests that Notch inhibition had enhanced the level of cone differentiation at this stage.

To further examine the effect of Notch signaling on the timing of cone genesis, we treated cultures for 48 hr with 10 μM DAPT or 10 μM RO4929097 (another Notch signaling inhibitor) at either d16 or d21 (~E16–E18 or ~P1–P3 in vivo, respectively) and analyzed gene expression in Crx-GFP$^+$ photoreceptors isolated and purified by FACS at d26 (Figure 2E). Transcripts of the cone-specific gene *Thrb2* were significantly more abundant (1.7- and 2.4-fold in cultures treated with DAPT and RO4929097, respectively; $p < 0.05$; $p < 0.01$, unpaired Student's t test)

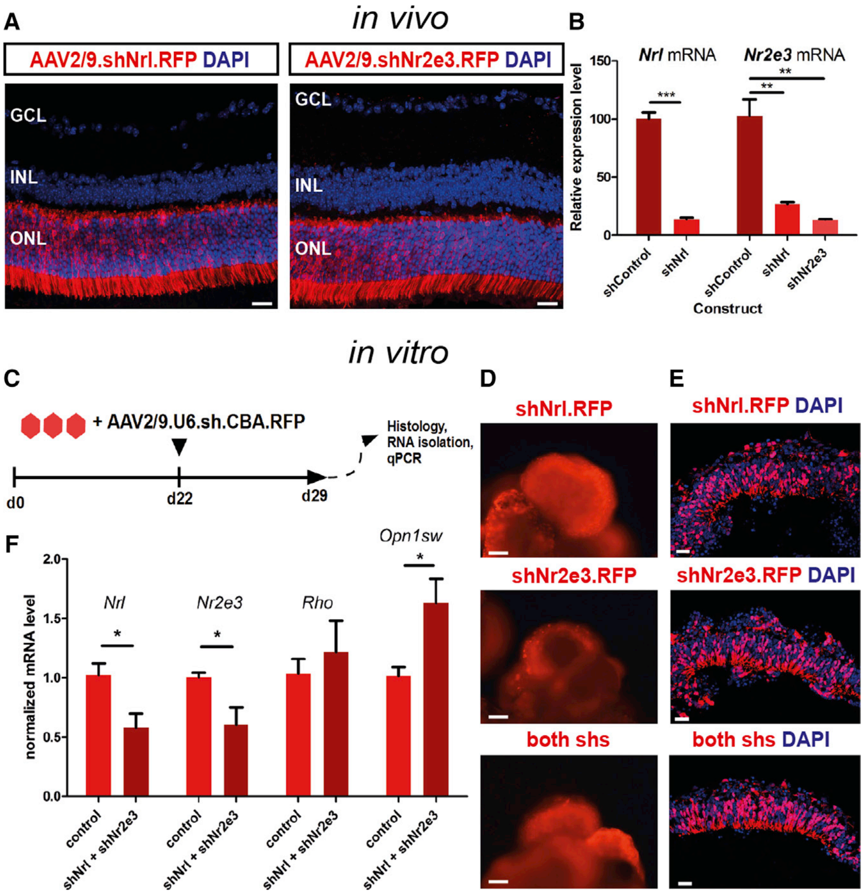

Figure 3. Knockdown of *Nrl* and *Nr2e3* De-represses S OPSIN Transcription In Vitro

(A) RFP fluorescent reporter expression following subretinal injection of AAV2/9 vectors encoding *shNrl* or *shNr2e3* driven by *U6* promoter and *CBA* promoter driving RFP transcription. GCL, ganglion cell layer; INL, inner nuclear layer; ONL, outer nuclear layer. Note confinement of transduction to the photoreceptor layer (ONL). Scale bar, 25 μm.

(B) qPCR gene expression analysis of *Nrl* and *Nr2e3* mRNAs in RFP^+ photoreceptors isolated by FACS normalized to non-targeting shControl-transduced photoreceptors; n = 3 retinas for each construct. **p < 0.01, ***p < 0.001, Student's t test

(C) Graphical depiction of the vector transduction experiment in vitro.

(D and E) transduced organoids at day 29 showing expression of RFP. (D) Whole organoids; (E) sections through optic vesicle regions. Scale bars, 100 μm (D) and 20 μm (E). Mean ± SD.

(F) qPCR expression analysis of *Nrl*, *Nr2e3*, *Rho*, and *Opn1sw* in infected versus control organoids. Note significant decreases in *Nrl* and *Nr2e3* associated with induction of S OPSIN (*Opn1sw*) expression. N = 4. Mean ± SD. *p < 0.05, Student's t test.

when Notch inhibition was performed at d16 of differentiation, compared with treatment at d21 (~E16 versus ~P2 in vivo). A similar pattern was detected for *Rxrg* and *Opn1sw* (Figure 2E), indicating a relatively higher level of cone differentiation following Notch signaling inhibition at an early, compared with a late, culture stage. Together, these data suggest a peak competence for cone genesis at an early phase of retinal histogenesis, as in vivo. Despite loss of laminar organization, further culture of organoids treated with DAPT at d16 led to expression of ARRESTIN3 (Figure 2F), RECOVERIN, and RHODOPSIN by d28 (Figure S2E), indicating that both cones and rods continue to mature after early Notch inhibition. Collectively, these observations demonstrate that mESC-derived retinal organoids respond to Notch signaling inhibition in a manner similar to that observed in vivo, and that timed application of Notch pathway inhibitors can be utilized to promote commitment to the cone fate.

Knockdown of *Nrl* and *Nr2e3* Reveals Limited Plasticity of Postmitotic Photoreceptor Precursors

In early postmitotic photoreceptor precursors, NRL directs commitment to the rod lineage (Mears et al., 2001). Conversely, NRL loss of function leads to acquisition of an S cone-like photoreceptor phenotype (Mears et al., 2001) and has been utilized to obtain enriched populations of S cone-like cells for transplantation studies (Santos-Ferreira et al., 2015; Smiley et al., 2016). To determine whether mESC-derived photoreceptor precursors show an equivalent fate plasticity, with a view to generating cone-enriched populations for transplantation, we developed knockdown constructs targeting the coding sequences of *Nrl* and *Nr2e3* with an RFP reporter. Since these genes are only expressed in rod photoreceptor precursors, the effect of the knockdown is limited to postmitotic rods. These constructs mediated very efficient *Nrl* and *Nr2e3* transcriptional silencing, as assessed by qPCR, when packaged into AAV2/9 serotype viral vectors and delivered subretinally into adult mouse retina (Figures 3A and 3B; reduction by 86.2% and 86.5%, n = 3; p < 0.001 and p < 0.01, respectively, unpaired Student's t test). Retinal organoids were cultured as depicted in Figure 1A and transduced at d22 (~P2 in vivo; Figures 3C–3E), at the end of peak of rod genesis and at which point relatively small numbers of actively dividing progenitors are present (Decembrini et al., 2014; Eiraku et al., 2011; Figures S4A and S4B). A significant increase

in S OPSIN transcripts was detected in transduced versus control cultures (N = 4, $p < 0.05$, unpaired t test; Figure 3F), consistent with the S cone enrichment observed in vivo. However, consistent with an analysis of early postnatal *Nrl*$^{-/-}$ mouse retina (Emerson et al., 2013), we could not detect upregulation of early cone differentiation genes *Thrb2* or *Rxrg* nor of a more mature marker *Arr3* in purified transduced photoreceptors, despite significant downregulation of *Nrl* and strong trends for *Nr2e3* and *Rorb* mRNA depletion (Figure S4G). There was no effect on RA signaling-related transcripts other than *Rorb* or the percentage of photoreceptors in culture (Figures S4F and S4H). Such an expression profile fits with the recent report describing evidence for S cone features during early rod development such as expression of S OPSIN transcripts in immature rods (Kim et al., 2016). Our results suggest that in cultures that received the *Nrl* and *Nr2e3* knockdown, developing rods remain at this immature stage, due to depletion of *Nrl* and *Nr2e3* transcripts. Thus, *Nrl*-depleted photoreceptors may represent an immature form of rods rather than true cone precursors, which may limit their utility in cone cell replacement.

Retinoic Acid Signaling Regulates Cone Precursor Maturation in mESC-Derived Retinas

To optimize cone differentiation in vitro, we sought to determine further the extrinsic signals regulating this process, focusing on RA signaling as a candidate pathway. Synthesis of RA is high in embryonic murine retina, where it appears to induce S OPSIN expression but declines postnatally (Alfano et al., 2011; McCaffrery et al., 1993). Conversely, application of exogenous RA at a late stage of zebrafish photoreceptor development blocks cone maturation (Hyatt et al., 1996). We first examined expression of RA signaling components during in vitro retinogenesis by qPCR. RA metabolism enzymes *Raldh1*, *Raldh3*, and *Cyp26a1* showed strong downregulation from d12 onward (Figure 4A), whereas levels of the receptors *Rara*, *Rorb*, and *Rxrg* remained relatively stable (Figure 4C). Immunostaining confirmed RALDH1 and retinoid X receptor γ (RXRγ) expression, localized to the neural retina (Figures 4B and 4D, respectively). Given that RA at embryonic stages is critical for S OPSIN induction (Alfano et al., 2011), we tested the effects of supplementing cultures with a pulse of 500 nM all-*trans* RA at d14–d16 (~E14–E16 in vivo; Figure 4E). This treatment significantly increased expression of the rod-specific transcription factor *Nrl*, but not the cone-specific genes *Thrb2* and *Rxrg*, by d21 in culture (Figure S5A). S OPSIN expression was upregulated by d26, but without change in the expression of *Arr3*, a cone phototransduction gene (Figure 4F). However, continued supplementation of RA (from d14 onward) strongly suppressed both S OPSIN and ARRESTIN3 expression by d26 (4.4- and 23.8-fold reduction, respectively, N = 4; $^*p < 0.05$; $^{***}p < 0.001$, Student's t test; Figure 4F). This transcriptional repression also correlated with decreased immunoreactivity for both S OPSIN and ARRESTIN3 in organoids continuously exposed to RA (Figures 4G and 4H). RA treatments did not affect cell viability, but early exposure to RA (d14–d16) modestly increased cell proliferation, determined by a bromodeoxyuridine pulse, which led to increased cell yields at later stages (Figures S5B–S5E). Despite the presence of S OPSIN$^+$ and ARRESTIN3$^+$ cells and expression of genes associated with cone maturation, including *Opn1mw* (Figures S5F–S5H), we did not observe M OPSIN protein by immunostaining in the organoids, even in prolonged culture (until d37). Collectively these data show that, as in development in vivo, temporal regulation of RA levels is important for onset of mESC-derived S cone precursor maturation and pulse administration of RA at a critical stage facilitates S OPSIN expression.

M OPSIN Is Induced in mESC-Derived Cone Precursors Transplanted into the Adult Mouse Retina

M OPSIN in murine retina is expressed postnatally (Ng et al., 2001). Prolonged culture of our retinal organoids to a corresponding stage did not lead to M OPSIN protein induction. Since this limitation is also observed in mouse retinal explant cultures (Söderpalm et al., 1994), we sought to determine whether lack of M OPSIN expression is due to an inherent incapacity of the mESC-derived cone precursors or is a consequence of the in vitro conditions. In a previous study, we showed that the adult host retinal environment supported the maturation of transplanted Crx-GFP$^+$ (mixed rod and cone) photoreceptor precursors derived from embryonic mouse retinas, including the expression of cone markers (Lakowski et al., 2010). We therefore isolated and purified by FACS Crx-GFP$^+$ cells from mESC-derived retinal organoids at d16 (~E16 in vivo) and injected them into the subretinal space of adult wild-type mice. When examined 3 weeks post transplantation, we observed that 35% (±18%) of surviving Crx-GFP$^+$ cells co-expressed the all-cone marker RXRγ (n = 8 sections, N = 3 eyes; Figure 5A). In addition, ARRESTIN3 staining was present in the subretinal graft (Figure 5B). Notably, both S OPSIN$^+$ (Figure 5C) and M OPSIN$^+$ processes (Figure 5D) were formed within the subretinal space (three out of five grafts examined) in similar numbers (22 ± 15 and 22 ± 12 per 100 cells, n = 3; Figure 5E). To obtain a cone precursor-enriched population comparable with the early postnatal donors used previously for donor-derived cone-like cells (Santos-Ferreira et al., 2015; Smiley et al., 2016), we used Crx-GFP$^+$ cells from d23 retinal organoids, which had been treated with DAPT at d16. As with the earlier staged cultures, Crx-GFP$^+$ cells also survived and expressed S OPSIN and/or M OPSIN (Figures 5F and

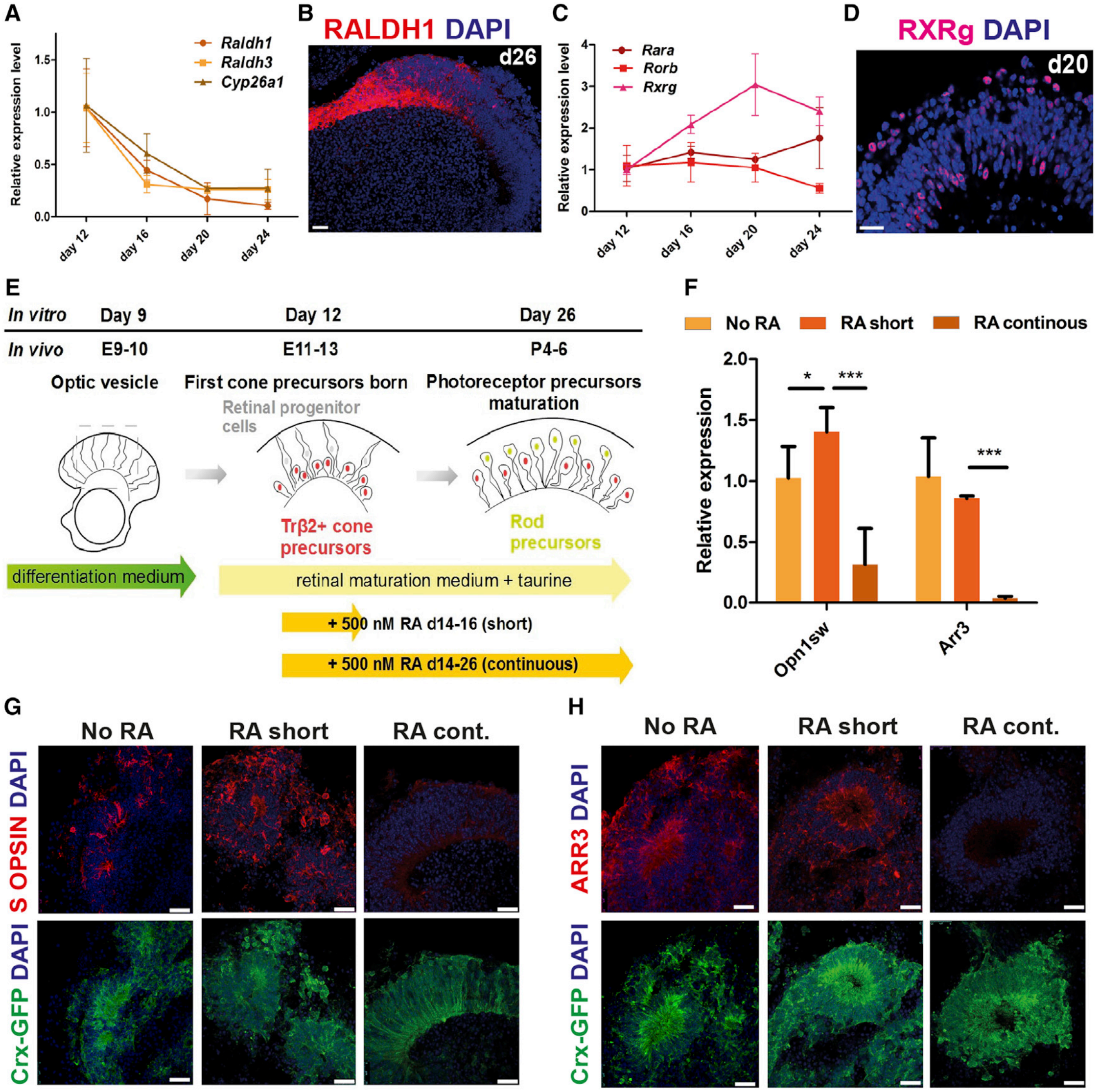

Figure 4. Cone Precursor Maturation in mESC-Derived Retinas Is Regulated by Retinoic Acid Signaling

(A) qPCR expression time course of RA metabolism enzymes *Raldh1*, *Raldh3*, and *Cyp26a1* in mESC retinal organoids; n = 3. Mean ± SD.

(B) Immunostaining for RALDH1 at day 26 in culture.

(C) qPCR expression time course of RA receptors *Rara*, *Rorb*, and *Rxrg*; n = 3. Mean ± SD.

(D) RXRγ antibody staining at day 20 of differentiation.

(E) Graphical illustration of the RA stimulation experiments.

(F) qPCR analysis of *Opn1sw* and *Arr3* expression at day 26 in cultures treated with RA as indicated. N = 4. Mean ± SD. *$p < 0.05$; ***$p < 0.001$, Student's t test.

(G and H) Fluorescent microscopy showing expression of cone-specific proteins S OPSIN (G) and ARRESTIN3 (H) and the Crx-GFP reporter in cultures treated as depicted in (E). Note the loss of cone marker immunoreactivity following continuous exposure to RA.

All scale bars, 20 μm.

5G), following transplantation into adult wild-type mice. We conclude that precursors derived from mESCs can undergo specification into both S OPSIN- and M OPSIN-expressing cones following transplantation into adult retina.

Retinal Differentiation of mESCs Provides a Robust Source of Purified Cone Precursors for Transplantation

To assess the feasibility of transplanting purified cone-only populations from a renewable source, we used viral labeling to tag mESC-derived cones for isolation, purification, and

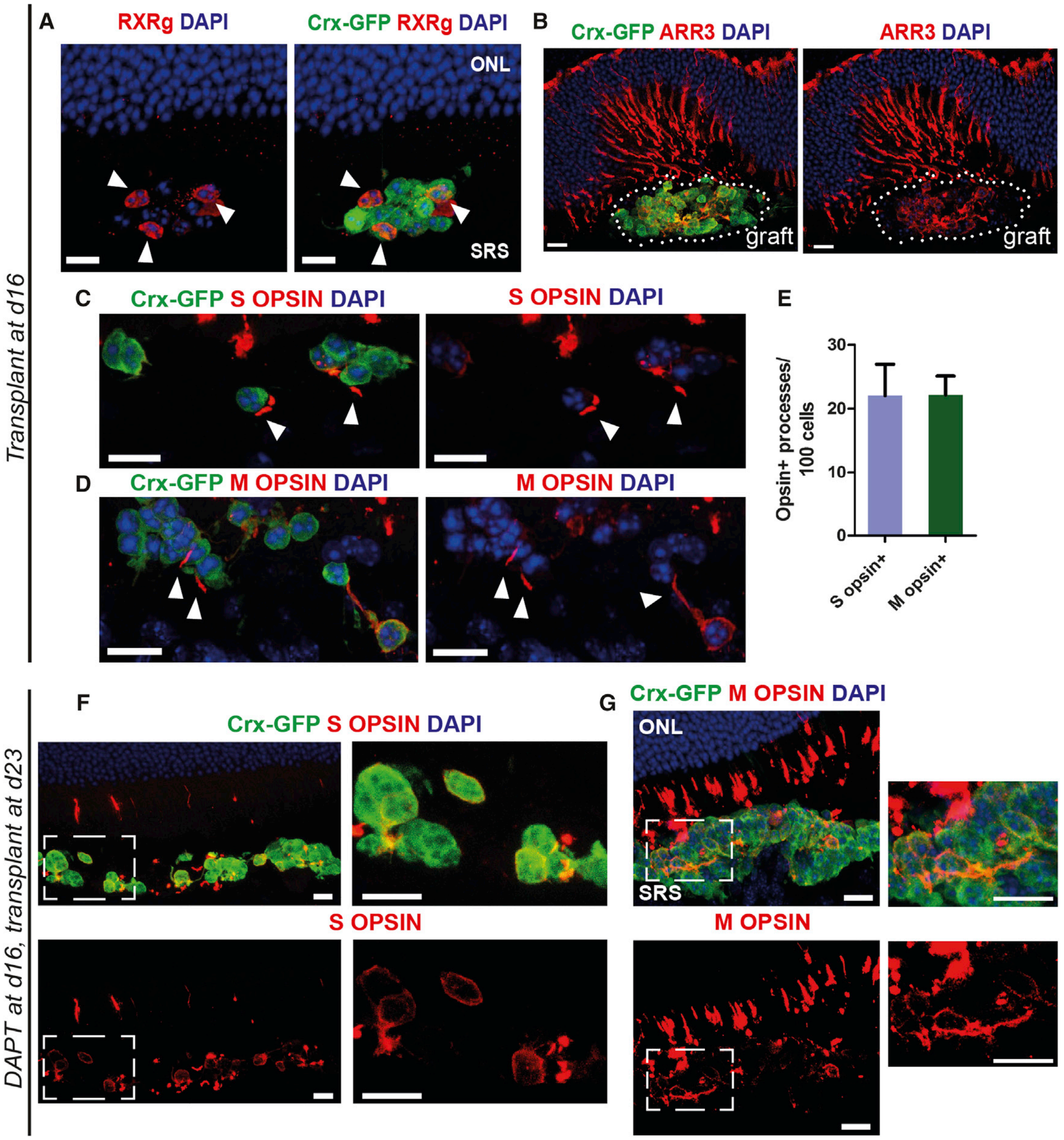

Figure 5. Cone Precursor-Enriched Populations Transplanted into Adult Retina Show Cone Maturation with M OPSIN Induction

(A–D) Subretinal cells masses of transplanted mESC-derived Crx-GFP^{+} photoreceptors isolated at day 16 of differentiation. Immunostaining for RXRγ, a nuclear receptor specific for cones and ganglion cells (A, arrowheads), cone marker ARRESTIN3 (B), and cone visual pigments S OPSIN (C, arrowheads) and M OPSIN (D, arrowheads). Note that M OPSIN expression was not observed in vitro.

(E) Assessment of S OPSIN- and M OPSIN-positive processes (arrowheads) in transplanted cells; n = 3 retinas. Mean ± SD.

(F and G) S OPSIN (F) and M OPSIN (G) expression, respectively, in subretinal cell mass of Crx-GFP^{+} photoreceptors isolated at day 23 from organoids treated with 10 μM DAPT at day 16 in culture. Higher-power views of selected regions (boxed) are shown in right panels. ONL, outer nuclear layer; SRS, subretinal space. All scale bars, 10 μm.

transplantation. We used an AAV2/9 vector (2.1.GFP) encoding the 2.1-kb fragment of the human red-green (M/L) cone OPSIN promoter (Wang et al., 1992), which drives specific GFP expression in mouse cones (Figure 6A). Since the majority of mouse cones co-express both S OPSIN and M OPSIN (Applebury et al., 2000) the 2.1.GFP reporter was expected to label most mESC-derived mouse cones. Transduced retinal organoids showed GFP-labeled cells in the optic vesicle regions (Figure 6B). GFP^{+} cells were not proliferating progenitors (Figures S6A and S6B) or rod

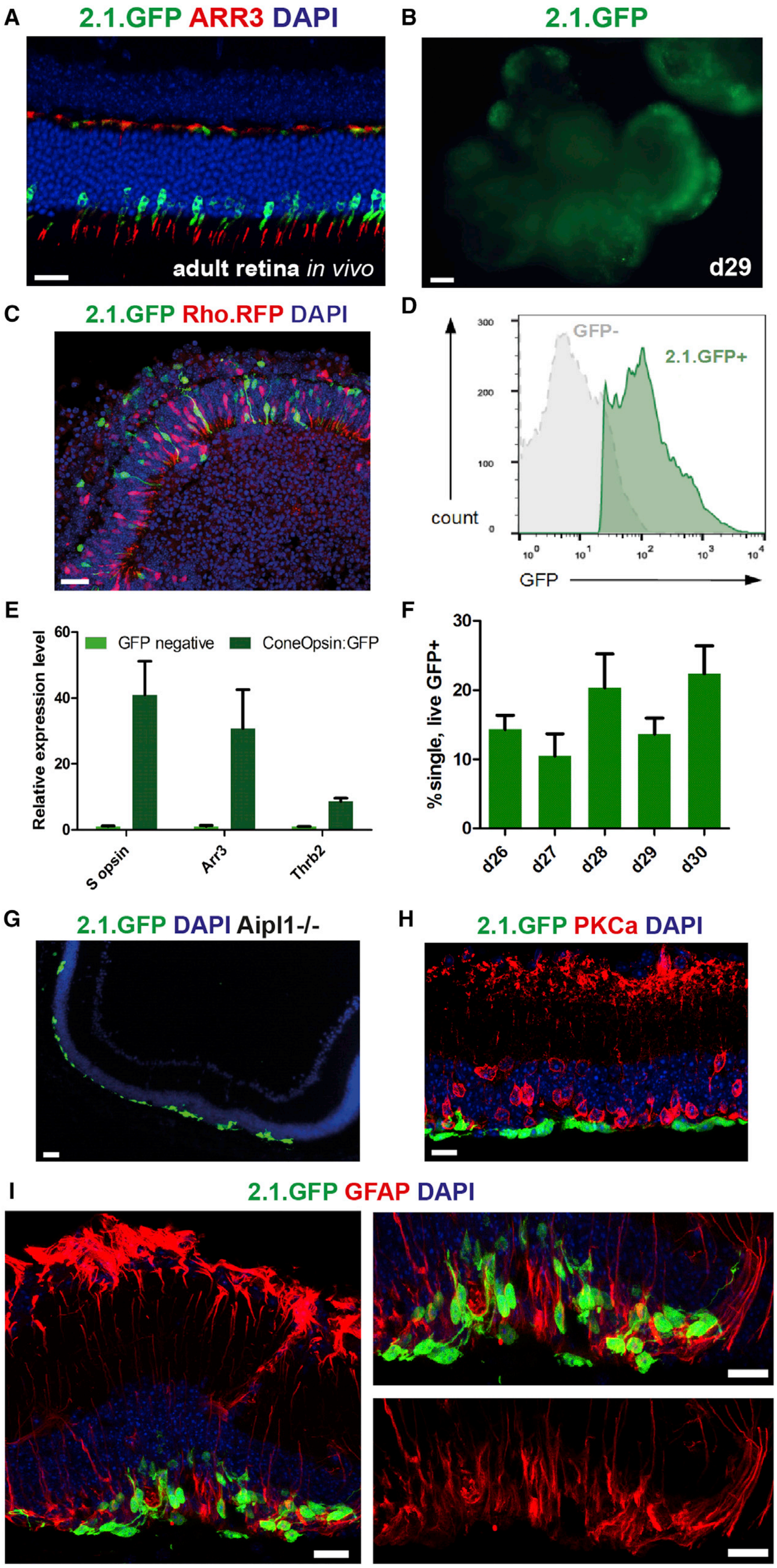

Figure 6. Isolation and Transplantation of Purified mESC-Derived Cone Precursors

(A) Reporter fluorescence driven by AAV2/9-2.1.GFP vector following subretinal injection into adult mouse retina. Scale bar, 20 μm.

(B) GFP fluorescence in whole retinal organoids at day 29 of differentiation infected with the AAV2/9-2.1.GFP vector at day 22. Scale bar, 100 μm.

(C) 2.1.GFP-expressing cells are distinct from rods labeled with vector encoding RHODOPSIN promoter driving RFP. Scale bar, 20 μm.

(D) Flow-cytometry histogram showing GFP fluorescence in dissociated cells from 2.1.GFP vector transduced organoids.

(E) qPCR showing enrichment of cone genes *S opsin*, *Arr3*, and *Thrb2* in GFP^{+} population compared with GFP^{-} cells at day 26; n = 3. Mean ± SD.

(F) Percentage of GFP^{+} cells. At least n = 3 sorts per time point. Mean ± SD.

(G) $2.1.GFP^{+}$ cells in the $Aipl1^{-/-}$ retina 3 weeks post transplantation. Scale bar, 20 μm.

(H) MESC-derived donor cells overlie $PKC\alpha^{+}$ host bipolar cells. Scale bar, 10 μm.

(I) Immunostaining for GFAP in a grafted retina. Note $GFAP^{+}$ processes extending into the donor cell mass. Scale bar, 10 μm.

precursors (labeled with Rhodopsin.RFP virus; Figure 6C) and purified using FACS at d26 (Figure 6D) were highly enriched for the cone-specific transcripts *Opn1sw*, *Arr3*, and *Thrb2* (Figure 6E), supporting their cone precursor identity. We used our standard culture protocol without DAPT (Figure 1A), but including an RA pulse for transplantation, since no gain in absolute cone yield was observed with DAPT treatment (Figure S6C). Strikingly, the average percentage of total isolated cells expressing GFP^+ at d26–d30 (~P6–P10 in vivo) was 15% ± 7% (n = 21 sorts; at least three independent sorts per time point [Figure 6D] with typical viability around 80% [Figure S6D]). This is markedly higher than the ~3% of photoreceptors that cones typically make up in the adult murine eye (Jeon et al., 1998). This high proportion of cone precursors allowed routine isolation in numbers sufficient for transplantation. We transplanted 2×10^5 cells per eye, similar to the total number of cone cells in an adult mouse retina (Jeon et al., 1998). Initially, transplantation was performed into adult wild-type mice (n = 29 eyes) to determine the feasibility of purified mESC-derived cone transplantation in a non-degenerative environment. Our optimized methodology resulted in a high success rate, with 26 out of 29 eyes (89%) supporting the survival of the transplanted cell mass (Figures S6E–S6H), without immune suppression. Reporter-positive photoreceptors were present in the host outer nuclear layer (Figure S6H), but similar to previous studies demonstrated rod-like morphology and were likely the result of uptake of cellular components by host photoreceptors from the subretinal graft (Decembrini et al., 2017; Ortin-Martinez et al., 2016). In the subretinal space, the cells were negative for rod markers RHODOPSIN and GNAT1 (Figures S6E and S6F) and continued to develop in vivo, forming peanut agglutinin (PNA)-labeled extracellular matrix and expressing ARRESTIN3 (Figures S6G and S6H).

Cone Replacement in *Aipl1*$^{-/-}$ Mice by mESC-Derived Precursors

To determine whether in vitro differentiated cones could replace lost host cones in a model of end-stage retinal degeneration, we used *Aipl1*$^{-/-}$ mice, which exhibit rapid photoreceptor loss (Ramamurthy et al., 2004). 2.1.GFP^+ cone precursors were injected into 8-week-old *Aipl1*$^{-/-}$ recipients and examined 3 weeks post transplantation. In all eyes examined, the donor cells survived (nine of nine injected eyes; Figure 6G) and were distributed over the remaining host interneurons (Figure 6H; $PKC\alpha^+$ bipolar cells in red). Close to the injection site the cells were clustered together and presented morphologies polarized toward the host retina, while further displaced cells were found with neurites extending more horizontally (Figures S6I and S6J). Müller cells, which support photoreceptor function, can become activated in retinal degenerations. In some types of disease they can form a glial scar that may impede donor/host interactions (reviewed by Pearson et al., 2014). Glial fibrillary acidic protein (GFAP), a marker of activated Müller cells, was upregulated in their basal processes (Figure 6I). Interestingly, at the interface between the graft and the host inner nuclear layer, we observed focal extension of Müller cell processes into the donor cell mass, potentially restoring the normal interaction between these two cell types (Figure 6I).

We next examined the in vivo maturation and potential connectivity of the transplanted cone precursors with the host retina. Expression of a panel of cone phototransduction-related proteins, including M OPSIN, GNAT2, and CNGB3 (Figures 7A–7C), was detected using immunostaining. PNA also labeled most of the transplanted cells (Figure S6K). Furthermore, donor cones formed distal processes directed toward the host retinal-pigmented epithelium, which were positive for the outer segment protein PERIPHERIN2 (Figure 7D), while basally directed neurites that extended toward the dendrites of host horizontal cells (Figure 7E) expressed the structural synapse protein, RIBEYE (Figure 7F), and the synaptic vesicle protein, SYNAPTOPHYSIN (Figure 7G). Collectively, these results indicate that mESC-derived cones may have the potential to replace lost host cones in a model of advanced retinal degeneration.

DISCUSSION

Retinal degenerations causing photoreceptor cell death are a major cause of untreatable blindness, for which photoreceptor replacement by transplantation may represent a promising treatment strategy. Transplanted dissociated photoreceptors can rescue visual function (Barnea-Cramer et al., 2016; Pearson et al., 2012; Santos-Ferreira et al., 2015; Singh et al., 2013). However, while useful human vision is mainly dependent on cone photoreceptors, most advances were made with rod photoreceptors. One of the reasons for this is the scarcity of cones, which, in the mouse, account for only around 3% of photoreceptors (Jeon et al., 1998), making their isolation in sufficient quantities for study problematic.

In this study, we combined mESC differentiation with progress in the understanding of retinal progenitor commitment to the cone photoreceptor lineage (Eiraku et al., 2011; Emerson et al., 2013; Hafler et al., 2012) to show that mESC-derived retinas exhibit competence to produce cone precursors and do so with high efficiency. Moreover, we find that timely inhibition of Notch signaling represents a more suitable approach for increasing cone proportion than silencing of the *Nrl*/*Nr2e3* rod differentiation pathway. Downregulation of *Nrl* only

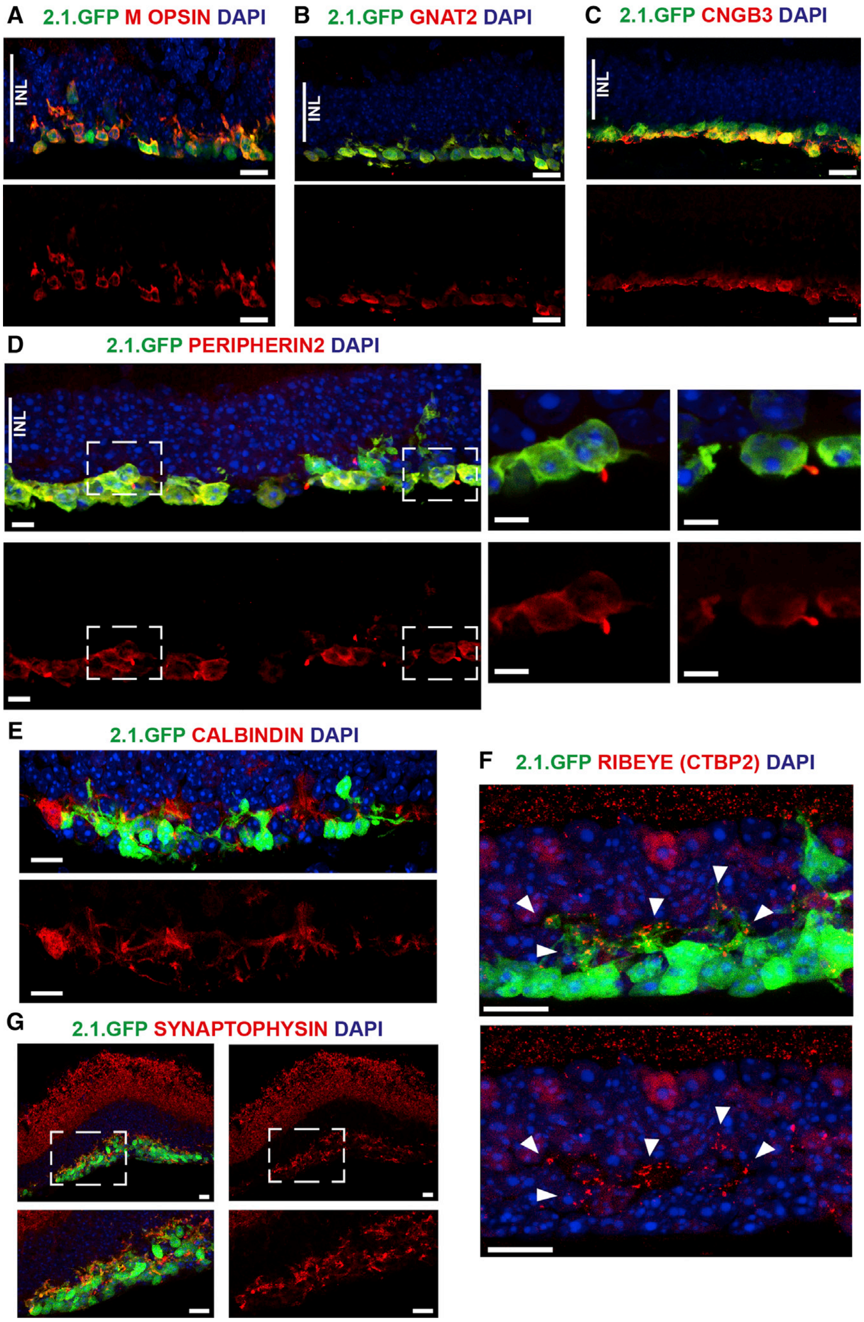

Figure 7. Cone Cell Replacement in Aipl1$^{-/-}$ Retinas

(A–C) Immunostaining for cone-specific phototransduction-related proteins M OPSIN (A), GNAT2 (B), and CNGB3 (C) in the transplanted 2.1.GFP$^+$ cones.

(D) PERIPHERIN2 staining showing accumulation in distal processes formed by transplanted cones. Panels on the right show a higher-power image of selected regions (boxed).

(E) 2.1.GFP$^+$ cells are in close proximity to CALBINDIN$^+$ host horizontal cell dendrites.

(F and G) Antibody staining for synaptic proteins RIBEYE (CTBP2; F) and SYNAPTOPHYSIN (G) showing expression in transplanted GFP$^+$ cells (arrowheads in F).

INL, inner nuclear layer. Scale bars, 5 μm (F) and 10 μm (all other images).

induced S OPSIN, whereas Notch inhibition additionally triggered increased expression of TRβ2 and RXRγ, permitting also M cone specification following transplantation. The high percentage of cone precursors generated in culture facilitated isolation and assessment of their transplantation potential. In vitro-generated cones showed reliable graft survival in the subretinal space of not only healthy recipients, but also in *Aipl1*$^{-/-}$ host eyes with severely degenerated retina.

While characterizing in vitro differentiation of cone precursors we made several observations regarding this process. The proportion of cone precursors was high using our protocol, which might be a consequence of poor generation/survival of other retinal neurons including rods. Alternatively, a soluble factor acting as a negative feedback signal for cone genesis, analogous to the action of GDF11 in retinal ganglion cell development (Kim et al., 2005), may fail to reach biologically relevant concentrations.

Timely inhibition of Notch suggested that the temporal competence confining the peak of cone genesis to early stages of retinogenesis (Carter-Dawson and LaVail, 1979) is preserved in mESC-derived retinas differentiated in vitro. Notch inhibition may increase commitment to cone fate or accelerate their differentiation. While the proportion of cones was increased at the time point used for transplantation (equivalent to early postnatal stage), there was no significant gain in total yield. Furthermore, we found that RA signaling plays an evolutionarily conserved role in regulating photoreceptor maturation. The addition of exogenous all-*trans* RA to late stages of photoreceptor differentiation in ESC-derived retinas led to the suppression of cone maturation. This is similar to observations that exogenous RA impairs cone maturation in the fish retina (Hyatt et al., 1996). Expression of enzymes responsible for RA synthesis declined significantly at late differentiation stages, in accordance with previously reported biochemical analyses showing RA to be abundant at early retinogenesis in vivo (when cones are born) but declining neonatally (when rods are produced [McCaffrery et al., 1993]). High levels of RA in embryonic retina might therefore act to prevent precocious cone maturation during embryonic development, while acting to stimulate rod differentiation (as also observed in mESC-derived retinas) in the neonatal retina. This could provide a mechanism to synchronize maturation of the two photoreceptor types despite the lag between their birth peaks.

When isolated from their differentiation niche and transplanted into an adult environment, mESC-derived cone precursors at equivalents of both embryonic and early postnatal stages are able to undergo further differentiation in vivo, similar to precursor cells derived from donor mice (Lakowski et al., 2010). Strikingly, many of these cells go on to express M OPSIN, even in the severely degenerate *Aipl1*$^{-/-}$ retina. The signals present in the mature host retina appear to trigger M OPSIN expression, not observed in vitro despite extended culture periods. This suggests that the developmental capacity for subsequent M cone differentiation is retained in the retinal organoid system, but certain inductive signals, such as thyroid hormone provision from the circulation (Lu et al., 2009), are likely missing.

Using a mESC retinal organoid system as a source, we could reproducibly transplant cones in numbers on a par with the total number of cones in an adult mouse retina (Jeon et al., 1998). We achieved extensive cone cell replacement in the *Aipl1*$^{-/-}$ mouse, a model of Leber congenital amaurosis end-stage retinal disease in which the vast majority of host cones are lost due to degeneration (Ramamurthy et al., 2004). Unsurprisingly, given the advanced degenerative state of the recipient retina, the morphology of transplanted cones was compromised. This contrasts with apparently mature features of GFP-labeled cells observed in cone transplants into non-degenerative retina, which are now understood to arise from cytoplasmic material transfer from the subretinal graft to host rods (Decembrini et al., 2017; Ortin-Martinez et al., 2016). Nonetheless, cones in the *Aipl1*$^{-/-}$ recipients appeared to make physical contact with inner retinal neurons and expressed components associated with synaptic transmission, alongside phototransduction-related proteins, suggesting advanced differentiation and maturation. Although beyond the scope of the present study, future studies will be required to investigate further the potential functionality of these transplanted cells.

In summary, we report the robust differentiation and subsequent transplantation of mESC-derived cone precursors (summarized in Figure S7). Cones differentiated in vitro following a specification sequence resembling that observed in vivo, indicating that mESC-derived retinal organoids represent a developmentally relevant donor source for investigating cone cell replacement. Following isolation and transplantation, these cells survived and showed a degree of maturation in photoreceptor-depleted retina of *Aipl1*$^{-/-}$ mice, a model of end-stage retinal degeneration. Our work provides an important proof of concept for future use of purified cone photoreceptor transplantation to treat central vision loss due to cone cell death.

EXPERIMENTAL PROCEDURES

In all experiments, n indicates the number of individual samples and N the number of independent repeats, as with separate differentiation batches. Full details of experimental methods are provided in Supplemental Experimental Procedures.

Mouse ESC Culture and Retinal Differentiation

E16 CEE line was maintained on gelatin-coated dishes in the presence of leukemia inhibitory factor (1,000 U/mL; Merck Millipore). Crx-GFP line was cultured in "2i medium" supplemented with 3 μM GSK-3 inhibitor CHIR99021 (Tocris Bioscience) and 10 μM MEK inhibitor PD0325901 (Tebu-bio). The differentiation was performed as previously described (Decembrini et al., 2014; Gonzalez-Cordero et al., 2013). In brief, 3,000 mESCs were added to each well of Nunclon Sphera ultralow-binding 96-well plates (Thermo Fisher Scientific) to form an embryoid body (EB). Growth factor-reduced Matrigel (VWR International) was added next day at a final concentration of 2%. On day 9, EBs were transferred into 24-well plates (12 EBs per well) for further culture. A modification of our previous protocol was supplementing cultures with 500 nM all-*trans* RA (Sigma-Aldrich) between days 14 and 16 only. Normoxic conditions were used throughout the culture period. γ-Secretase inhibitors DAPT (Merck Millipore) and RO4929097 (BioVision) or DMSO as a vehicle control were added at a final concentration of 10 μM.

Immunohistochemistry

Organoids were fixed for 30 min in 4% formaldehyde, washed with PBS, and incubated overnight in 20% (w/v) sucrose, prior to embedding in OCT matrix. Tissue was cut into 18-μm cryosections mounted on glass slides, air-dried for 20 min, and kept frozen at −20°C for use in immunostaining. Staining protocol and the antibodies used are listed in Supplemental Experimental Procedures.

Dissociation and Fluorescence-Activated Cell Sorting

Organoids were dissociated using a papain-based Neural Tissue Dissociation Kit (Miltenyi Biotec) prior to sorting on a BD Influx Cell Sorter (Becton Dickinson). Flow-sorted GFP^{+} cells were on average >95% pure and >80% viable. Cells were resuspended at a final concentration of 1×10^5 live cells/μL in sterile Hank's balanced salt solution ($+Ca^{2+}$, $+Mg^{2+}$) and DNase I (50 U/mL) before injection.

Transplantation of mESC-Derived Photoreceptors

Full details of the surgical procedure can be found in Supplemental Experimental Procedures. In brief, 1 μL of cell suspension was injected into the superior retina and another 1 μL into the inferior retina. Adult mice were between 8 and 16 weeks of age at the time of transplantation. All animals were housed under a normal 12/12-hr light/dark cycle. Eyes were harvested 3 weeks after transplantation.

SUPPLEMENTAL INFORMATION

Supplemental Information includes Supplemental Experimental Procedures, seven figures, and four tables and can be found with this article online at http://dx.doi.org/10.1016/j.stemcr.2017.04.030.

AUTHOR CONTRIBUTIONS

K.K. contributed to the conception, design, execution, and analysis of all experiments and writing of the manuscript; A.G.C. contributed to the design and analysis of a number of the experiments; D.G. contributed to the analysis of a number of the experiments; A.N., M.J., S.J.I.B., and M.K., contributed to the maintenance and differentiation of stem cell cultures; Y.D. contributed to histological processing and surgery; A.G. contributed to the design of experiments; R.D.S. contributed to FACS and flow cytometer analysis; R.N.M performed viral purification; A.J.S. contributed interpretation of experiments and manuscript writing; S.D. and Y.I. provided reagents and contributed to revision of the manuscript; J.C.S. contributed to revision of the manuscript and funding; R.A.P. contributed to the conception, design and interpretation of experiments, subretinal surgery, manuscript writing, and funding; E.L.W. contributed to the conception, design and interpretation of experiments, and manuscript writing; R.R.A. contributed to the conception, design and interpretation of experiments, manuscript writing, and funding.

ACKNOWLEDGMENTS

This work was supported by the Medical Research Council UK (MR/J004553/1, MR/M007871/1, MR/L012758/1), European Research Council (ERC-2012-ADG_20120314), the Macular Vision Research Foundation, Fight for Sight (1448/1449), RP Fighting Blindness (GR576) The Miller's Trust, the Moorfields Eye Charity, and a generous donation by Mr. Otto van der Wyck. K.K. received a Wellcome Trust (WT083345MA) PhD studentship. D.G. is supported by A Star Singapore. R.A.P. received an Alcon Research Institute grant. J.C.S. is supported by Great Ormond Street Hospital Children's Charity and the NIHR Biomedical Research Center (NIHR BRC). R.R.A is partly funded by the NIHR BRC. We thank S. Azam for vector purification and L. Abelleira Hervas for assistance with animal work.

Received: October 24, 2016
Revised: April 25, 2017
Accepted: April 26, 2017
Published: May 25, 2017

REFERENCES

Alfano, G., Conte, I., Caramico, T., Avellino, R., Arno, B., Pizzo, M.T., Tanimoto, N., Beck, S.C., Huber, G., Dolle, P., et al. (2011). Vax2 regulates retinoic acid distribution and cone opsin expression in the vertebrate eye. Development *138*, 261–271.

Applebury, M.L., Antoch, M.P., Baxter, L.C., Chun, L.L.Y., Falk, J.D., Farhangfar, F., Kage, K., Krzystolik, M.G., Lyass, L.A., and Robbins, J.T. (2000). The murine cone photoreceptor: a single cone type expresses both S and M opsins with retinal spatial patterning. Neuron *27*, 513–523.

Assawachananont, J., Mandai, M., Okamoto, S., Yamada, C., Eiraku, M., Yonemura, S., Sasai, Y., and Takahashi, M. (2014). Transplantation of embryonic and induced pluripotent stem cell-derived 3D retinal sheets into retinal degenerative mice. Stem Cell Rep. *2*, 662–674.

Barnea-Cramer, A.O., Wang, W., Lu, S.-J., Singh, M.S., Luo, C., Huo, H., McClements, M.E., Barnard, A.R., MacLaren, R.E., and Lanza, R. (2016). Function of human pluripotent stem cell-derived photoreceptor progenitors in blind mice. Sci. Rep. *6*, 29784.

Carter-Dawson, L.D., and LaVail, M.M. (1979). Rods and cones in the mouse retina. II. Autoradiographic analysis of cell generation using tritiated thymidine. J. Comp. Neurol. *188*, 263–272.

Decembrini, S., Koch, U., Radtke, F., Moulin, A., and Arsenijevic, Y. (2014). Derivation of traceable and transplantable photoreceptors from mouse embryonic stem cells. Stem Cell Rep. *2*, 853–865.

Decembrini, S., Martin, C., Sennlaub, F., Chemtob, S., Biel, M., Samardzija, M., Moulin, A., Behar-Cohen, F., and Arsenijevic, Y. (2017). Cone genesis tracing by the Chrnb4-EGFP mouse line: evidences of cellular material fusion after cone precursor transplantation. Mol. Ther. J. *25*, 634–653.

Eiraku, M., Takata, N., Ishibashi, H., Kawada, M., Sakakura, E., Okuda, S., Sekiguchi, K., Adachi, T., and Sasai, Y. (2011). Self-organizing optic-cup morphogenesis in three-dimensional culture. Nature *472*, 51–56.

Emerson, M.M., Surzenko, N., Goetz, J.J., Trimarchi, J., and Cepko, C.L. (2013). Otx2 and Onecut1 promote the fates of cone photoreceptors and horizontal cells and repress rod photoreceptors. Dev. Cell *26*, 59–72.

Gonzalez-Cordero, A., West, E.L., Pearson, R.A., Duran, Y., Carvalho, L.S., Chu, C.J., Naeem, A., Blackford, S.J.I., Georgiadis, A., Lakowski, J., et al. (2013). Photoreceptor precursors derived from three-dimensional embryonic stem cell cultures integrate and mature within adult degenerate retina. Nat. Biotechnol. *31*, 741–747.

Hafler, B.P., Surzenko, N., Beier, K.T., Punzo, C., Trimarchi, J.M., Kong, J.H., and Cepko, C.L. (2012). Transcription factor Olig2 defines subpopulations of retinal progenitor cells biased toward specific cell fates. Proc. Natl. Acad. Sci. USA *109*, 7882–7887.

Hyatt, G.A., Schmitt, E.A., Fadool, J.M., and Dowling, J.E. (1996). Retinoic acid alters photoreceptor development in vivo. Proc. Natl. Acad. Sci. USA *93*, 13298–13303.

Jadhav, A.P. (2006). Notch 1 inhibits photoreceptor production in the developing mammalian retina. Development *133*, 913–923.

Jayakody, S.A., Gonzalez-Cordero, A., Ali, R.R., and Pearson, R.A. (2015). Cellular strategies for retinal repair by photoreceptor replacement. Prog. Retin. Eye Res. *46*, 31–66.

Jeon, C.J., Strettoi, E., and Masland, R.H. (1998). The major cell populations of the mouse retina. J. Neurosci. *18*, 8936–8946.

Kim, J., Wu, H.-H., Lander, A.D., Lyons, K.M., Matzuk, M.M., and Calof, A.L. (2005). GDF11 controls the timing of progenitor cell competence in developing retina. Science *308*, 1927–1930.

Kim, J.-W., Yang, H.-J., Oel, A.P., Brooks, M.J., Jia, L., Plachetzki, D.C., Li, W., Allison, W.T., and Swaroop, A. (2016). Recruitment of rod photoreceptors from short-wavelength-sensitive cones during the evolution of nocturnal vision in mammals. Dev. Cell *37*, 520–532.

Lakowski, J., Baron, M., Bainbridge, J., Barber, A.C., Pearson, R.A., Ali, R.R., and Sowden, J.C. (2010). Cone and rod photoreceptor transplantation in models of the childhood retinopathy Leber congenital amaurosis using flow-sorted Crx-positive donor cells. Hum. Mol. Genet. *19*, 4545–4559.

Lu, A., Ng, L., Ma, M., Kefas, B., Davies, T.F., Hernandez, A., Chan, C.-C., and Forrest, D. (2009). Retarded developmental expression and patterning of retinal cone opsins in hypothyroid mice. Endocrinology *150*, 1536–1544.

MacLaren, R.E., Pearson, R.A., MacNeil, A., Douglas, R.H., Salt, T.E., Akimoto, M., Swaroop, A., Sowden, J.C., and Ali, R.R. (2006). Retinal repair by transplantation of photoreceptor precursors. Nature *444*, 203–207.

McCaffrery, P., Posch, K.C., Napoli, J.L., Gudas, L., and Dräger, U.C. (1993). Changing patterns of the retinoic acid system in the developing retina. Dev. Biol. *158*, 390–399.

Mears, A.J., Kondo, M., Swain, P.K., Takada, Y., Bush, R.A., Saunders, T.L., Sieving, P.A., and Swaroop, A. (2001). Nrl is required for rod photoreceptor development. Nat. Genet. *29*, 447–452.

Meyer, J.S., Shearer, R.L., Capowski, E.E., Wright, L.S., Wallace, K.A., McMillan, E.L., Zhang, S.-C., and Gamm, D.M. (2009). Modeling early retinal development with human embryonic and induced pluripotent stem cells. Proc. Natl. Acad. Sci. USA *106*, 16698–16703.

Mizeracka, K., DeMaso, C.R., and Cepko, C.L. (2013). Notch1 is required in newly postmitotic cells to inhibit the rod photoreceptor fate. Development *140*, 3188–3197.

Muranishi, Y., Sato, S., Inoue, T., Ueno, S., Koyasu, T., Kondo, M., and Furukawa, T. (2010). Gene expression analysis of embryonic photoreceptor precursor cells using BAC-Crx-EGFP transgenic mouse. Biochem. Biophys. Res. Commun. *392*, 317–322.

Nakano, T., Ando, S., Takata, N., Kawada, M., Muguruma, K., Sekiguchi, K., Saito, K., Yonemura, S., Eiraku, M., and Sasai, Y. (2012). Self-formation of optic cups and storable stratified neural retina from human ESCs. Cell Stem Cell *10*, 771–785.

Nelson, B.R., Hartman, B.H., Georgi, S.A., Lan, M.S., and Reh, T.A. (2007). Transient inactivation of Notch signaling synchronizes differentiation of neural progenitor cells. Dev. Biol. *304*, 479–498.

Ng, L., Hurley, J.B., Dierks, B., Srinivas, M., Saltó, C., Vennström, B., Reh, T.A., and Forrest, D. (2001). A thyroid hormone receptor that is required for the development of green cone photoreceptors. Nat. Genet. *27*, 94–98.

Ng, L., Ma, M., Curran, T., and Forrest, D. (2009). Developmental expression of thyroid hormone receptor β2 protein in cone photoreceptors in the mouse. Neuroreport *20*, 627–631.

Nishida, A., Furukawa, A., Koike, C., Tano, Y., Aizawa, S., Matsuo, I., and Furukawa, T. (2003). Otx2 homeobox gene controls retinal photoreceptor cell fate and pineal gland development. Nat. Neurosci. *6*, 1255–1263.

Ortin-Martinez, A., Tsai, E.L.S., Nickerson, P.E., Bergeret, M., Lu, Y., Smiley, S., Comanita, L., and Wallace, V.A. (2016). A reinterpretation of cell transplantation: GFP transfer from donor to host photoreceptors. Stem Cells *35*, 932–939.

Pearson, R.A., Barber, A.C., Rizzi, M., Hippert, C., Xue, T., West, E.L., Duran, Y., Smith, A.J., Chuang, J.Z., Azam, S.A., et al. (2012). Restoration of vision after transplantation of photoreceptors. Nature *485*, 99–103.

Pearson, R.A., Hippert, C., Graca, A.B., and Barber, A.C. (2014). Photoreceptor replacement therapy: challenges presented by the diseased recipient retinal environment. Vis. Neurosci. *31*, 333–344.

Pearson, R.A., Gonzalez-Cordero, A., West, E.L., Ribeiro, J.R., Aghaizu, N., Goh, D., Sampson, R.D., Georgiadis, A., Waldron, P.V., Duran, Y., et al. (2016). Donor and host photoreceptors engage in material transfer following transplantation of post-mitotic photoreceptor precursors. Nat. Commun. *7*, 13029.

Ramamurthy, V., Niemi, G.A., Reh, T.A., and Hurley, J.B. (2004). Leber congenital amaurosis linked to AIPL1: a mouse model reveals destabilization of cGMP phosphodiesterase. Proc. Natl. Acad. Sci. USA *101*, 13897–13902.

Reh, T.A. (2016). Photoreceptor transplantation in late stage retinal degeneration. Invest. Ophthalmol. Vis. Sci. *57*, ORSFg1–7.

Roberts, M.R., Hendrickson, A., McGuire, C.R., and Reh, T.A. (2005). Retinoid X receptor (gamma) is necessary to establish the S-opsin gradient in cone photoreceptors of the developing mouse retina. Invest. Ophthalmol. Vis. Sci. *46*, 2897–2904.

Santos-Ferreira, T., Postel, K., Stutzki, H., Kurth, T., Zeck, G., and Ader, M. (2015). Daylight vision repair by cell transplantation: daylight vision repair by cell transplantation. Stem Cells *33*, 79–90.

Seiler, M.J., and Aramant, R.B. (2012). Cell replacement and visual restoration by retinal sheet transplants. Prog. Retin. Eye Res. *31*, 661–687.

Shirai, H., Mandai, M., Matsushita, K., Kuwahara, A., Yonemura, S., Nakano, T., Assawachananont, J., Kimura, T., Saito, K., Terasaki, H., et al. (2016). Transplantation of human embryonic stem

cell-derived retinal tissue in two primate models of retinal degeneration. Proc. Natl. Acad. Sci. USA *113*, E81–E90.

Singh, M.S., Charbel Issa, P., Butler, R., Martin, C., Lipinski, D.M., Sekaran, S., Barnard, A.R., and MacLaren, R.E. (2013). Reversal of end-stage retinal degeneration and restoration of visual function by photoreceptor transplantation. Proc. Natl. Acad. Sci. USA *110*, 1101–1106.

Smiley, S., Nickerson, P.E., Comanita, L., Daftarian, N., El-Sehemy, A., Tsai, E.L.S., Matan-Lithwick, S., Yan, K., Thurig, S., Touahri, Y., et al. (2016). Establishment of a cone photoreceptor transplantation platform based on a novel cone-GFP reporter mouse line. Sci. Rep. *6*, 22867.

Söderpalm, A., Szél, A., Caffé, A.R., and van Veen, T. (1994). Selective development of one cone photoreceptor type in retinal organ culture. Invest. Ophthalmol. Vis. Sci. *35*, 3910–3921.

Swaroop, A., Kim, D., and Forrest, D. (2010). Transcriptional regulation of photoreceptor development and homeostasis in the mammalian retina. Nat. Rev. Neurosci. *11*, 563–576.

Wang, Y., Macke, J.P., Merbs, S.L., Zack, D.J., Klaunberg, B., Bennett, J., Gearhart, J., and Nathans, J. (1992). A locus control region adjacent to the human red and green visual pigment genes. Neuron *9*, 429–440.

Yaron, O. (2006). Notch1 functions to suppress cone-photoreceptor fate specification in the developing mouse retina. Development *133*, 1367–1378.

Zhong, X., Gutierrez, C., Xue, T., Hampton, C., Vergara, M.N., Cao, L.-H., Peters, A., Park, T.S., Zambidis, E.T., Meyer, J.S., et al. (2014). Generation of three-dimensional retinal tissue with functional photoreceptors from human iPSCs. Nat. Commun. *5*, 4047.

Stem Cell Reports, Volume *8*

Supplemental Information

Differentiation and Transplantation of Embryonic Stem Cell-Derived Cone Photoreceptors into a Mouse Model of End-Stage Retinal Degeneration

Kamil Kruczek, Anai Gonzalez-Cordero, Debbie Goh, Arifa Naeem, Mindaugas Jonikas, Samuel J.I. Blackford, Magdalena Kloc, Yanai Duran, Anastasios Georgiadis, Robert D. Sampson, Ryea N. Maswood, Alexander J. Smith, Sarah Decembrini, Yvan Arsenijevic, Jane C. Sowden, Rachael A. Pearson, Emma L. West, and Robin R. Ali

Supplemental Figures

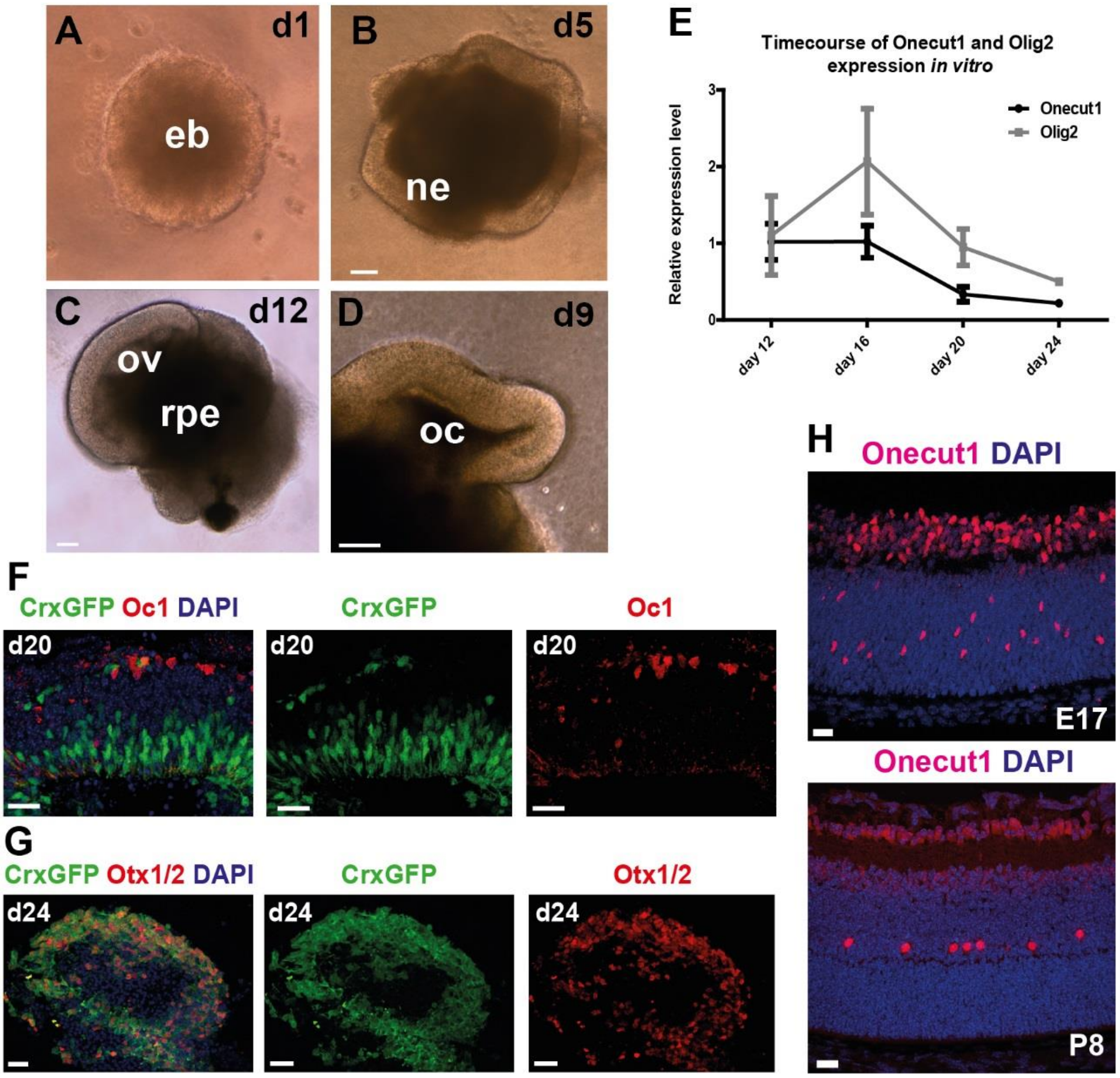

Figure S1. Differentiation of mESC into optic tissue expressing markers of cone-biased retinal progenitors. Related to Figure 1.

(A-D) Retinal differentiation cultures. Organoids at the stage of embryoid body (d1; A), retinal neuroepithelium formation (d5; B), optic cup (d9; C) and evaginated optic vesicle (d12; D). Scale bars 100 µm. **(E)** QPCR mRNA expression timecourse in cultures for Onecut1 and Olig2. Expression levels normalized to d12 of differentiation. **(F)** Immunostaining for Onecut1 (Oc1) in Crx-GFP mESC line retinal neuroepithelium at d20. Note that expression of Onecut1 is primarily localized to the basal side of the tissue rather than apical, where developing Crx-GFP+ photoreceptor precursors reside. Scale bar 20 µm. (**G**) Immunostaining for Otx2 in Crx-GFP line organoids at d24. Extensive overlap in immunoreactivity is observed in the retinal neuroepithelium at this stage. Scale bar 20 µm. **(H)** Onecut1 immunostaining in developing retina. Staining becomes restricted to horizontal cells by P8 (right panel). Scale bar 10 µm. eb – embryoid body; ne – neuroepithelium; oc – optic cup; ov – optic vesicle; rpe – retinal pigment epithelium.

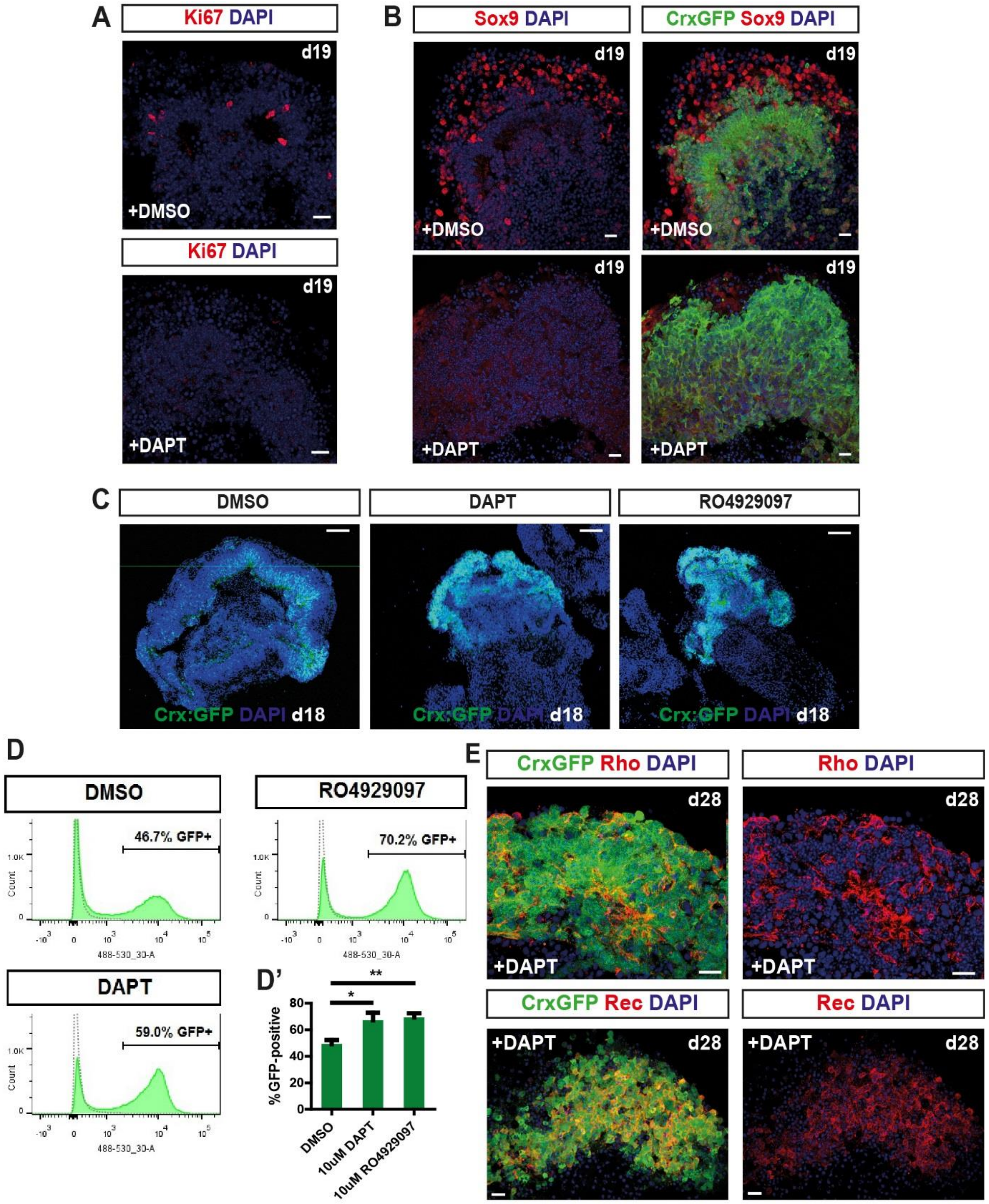

Figure S2. Regulation of photoreceptor neurogenesis by Notch signalling. Related to Figure 2.

(A) Loss of Ki67 staining following treatment with Notch signalling inhibitor DAPT at 10 µM between d14-d16. Scale bar 10 µm. **(B)** Reduction in immunostaining for retinal progenitor marker Sox9 following DAPT treatment. **(C)** Induction of Crx-GFP reporter expression at d18 in organoids treated with 10 µM DAPT or 10 µM RO4929097 at d14-d16. Scale bars 100 µm. **(D)** Representative flow cytometry histograms showing the proportion of Crx-GFP+ photoreceptor precursors following Notch inhibitor treatment in dissociated cultures at d19. **(D')** Quantification of flow cytometry results. N=5 experiments, >30 organoids per sample per experiment. *, $p<0.05$; **, $p<0.01$ one-way ANOVA with Tukey's *post hoc* test. **(E)** Rhodopsin and Recoverin immunostaining at d28 of differentiation in Crx-GFP retinal organoids treated with 10 µM DAPT at d16. Note the presence of these maturation markers despite loss of laminar organisation. Scale bar in all panels 10 µm.

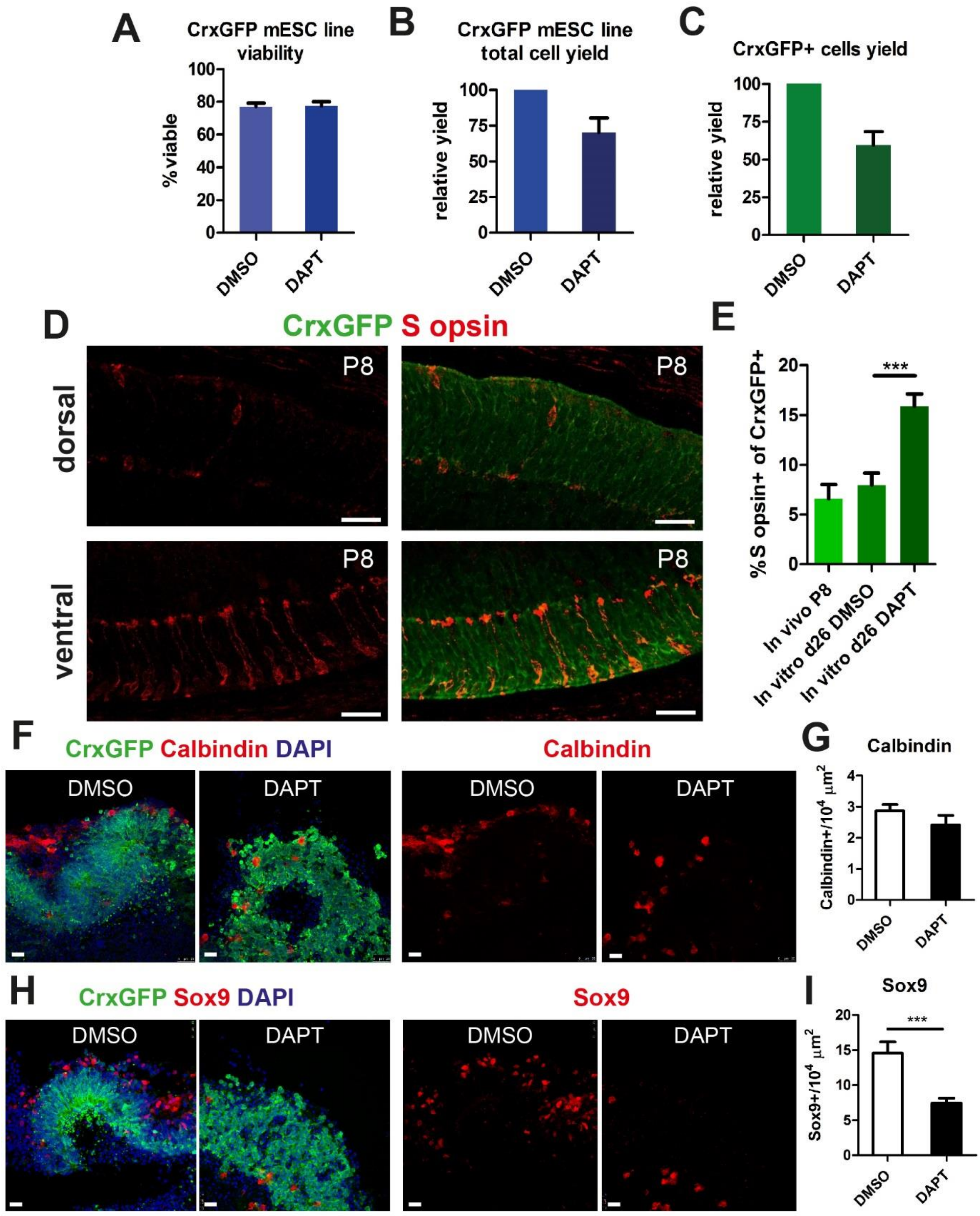

Figure S3. Impact of Notch inhibition on abundance of retinal neuron populations. Related to Figure 2. (A) Viability of dissociated control and DAPT-treated cultures. N=3, 36 organoids per experiment, mean ±SEM. **(B)** Cell yield determined by flow cytometry and viability analysis normalised to the DMSO control samples. N=3, 36 organoids per experiment, mean ±SEM. **(C)** Crx-GFP+ cell yield for the samples in (B). **(D)** Expression of S opsin in P8 retina in vivo. Upper panels dorsal region, lower panels ventral region. **(E)** Quantification of S opsin+ cells in P8 retina in vivo. N=3 eyes from individual animals, n=9 sections per eye from dorsal, mid-central and ventral parts of the retina. Data from retinal organoids from Fig.2 presented for comparison. **(F-I)** Immunostaining (F,H) and quantification (G,I) of calbindin+ horizontal cells (F,G) and Sox9+ late progenitors/ Müller glia (H,I). Scale bar 10 μm. Cell number normalised to organoid area; mean ±SEM, n>25, N=3, $p<0.001$ unpaired Student's t-test.

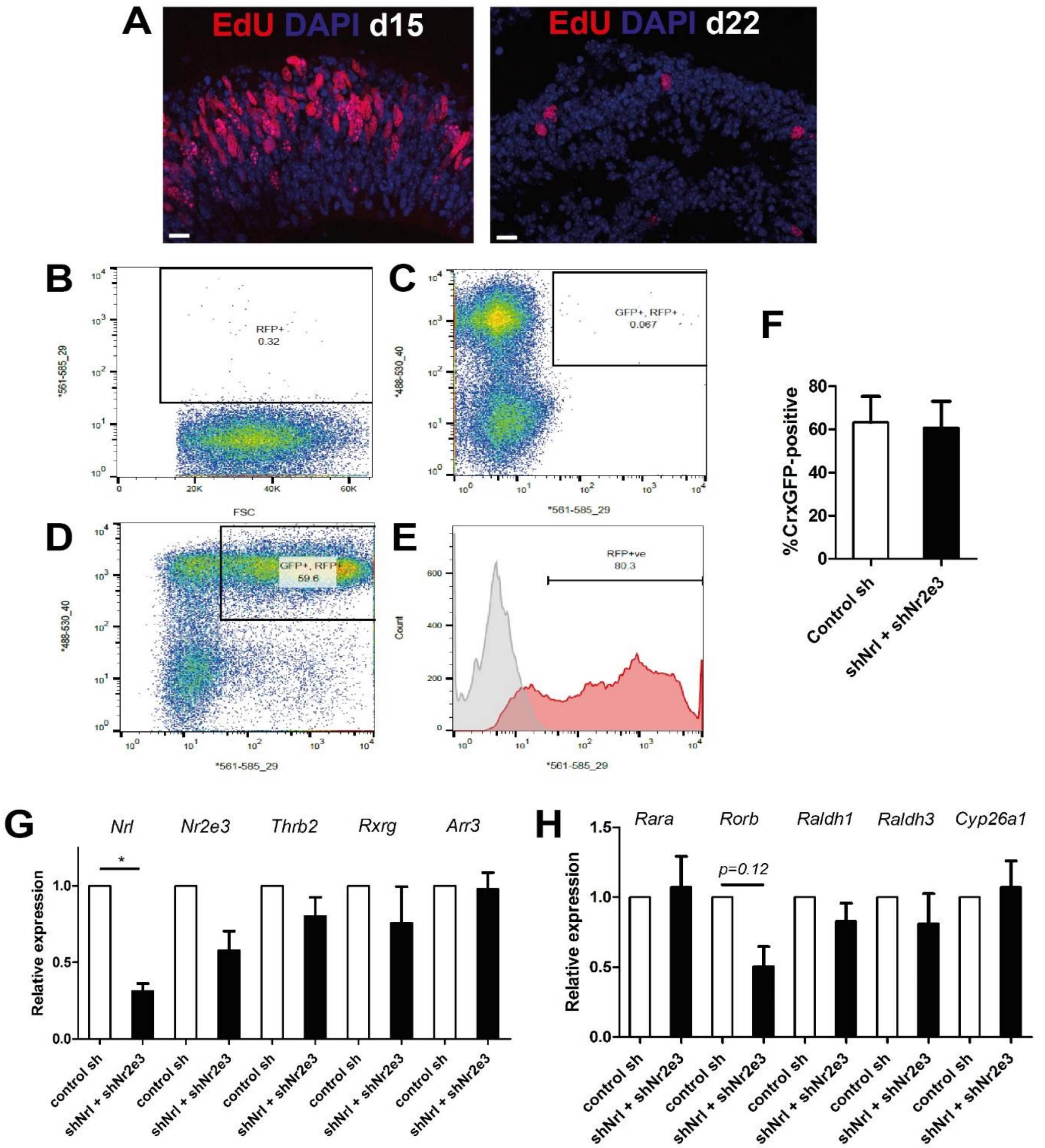

Figure S4. Knockdown of *Nrl* and *Nr2e3* in isolated photoreceptor precursors. Related to Figure 3.

(A) EdU pulse immunodetection in retinal organoids at d15 or d22. 10 µM EdU was added to culture media for 1 hr before harvest. Note the low number of proliferating cells at d22. Scale bar 10 µm. **(B-E)** Isolation and purification of post-mitotic photoreceptor precursors transduced with Nrl and Nr2e3 knockdown constructs by FACS. (B,C) Representative scatter plots showing gating strategy for collection of RFP+ (B) and GFP+ /RFP+ double positive cells (C) using non-transduced Crx:GFP dissociated control cultures. (D) Representative scatter plot showing collection of Crx:GFP photoreceptor precursors expressing shNrl and shNr2e3 vectors. Note the presence of a distinct GFP+RFP+ population. (E) Histogram showing RFP expression in Crx:GFP photoreceptor precursors transduced with shNrl and Nr2e3 vectors. **(F)** Percentage of Crx-GFP cells determined by flow cytometry in the knockdown experiments. N=4 samples analysed. **(G, H)** QPCR gene expression analysis of photoreceptor-specific genes (G) and RA signalling-related transcripts (H) using RNA isolated from flow-sorted either control or knockdown construct transduced CrxGFP+ photoreceptor precursors; n=7 samples, N=6 differentiation cultures; *, $p<0.05$, Wilcoxon matched pairs test.

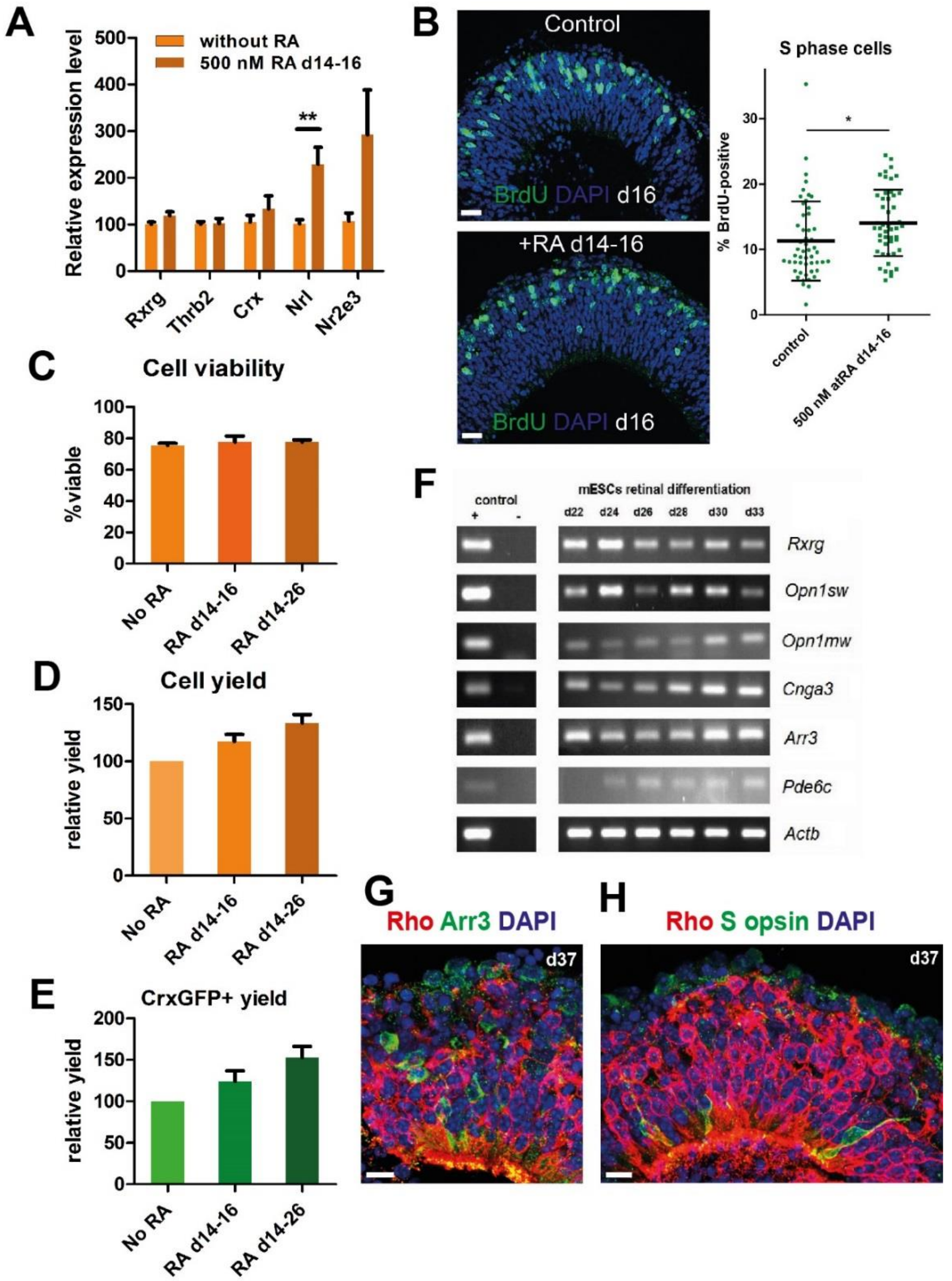

Figure S5. Retinoic acid supplementation and retinal organoid development. Related to Figure 4.

(A) QPCR analysis of cultures treated with a pulse of 500 nM RA at d14-d16 normalized to control cultures without the supplementation. Note the induction of *Nrl* and *Nr2e3* without effect on *Thrb2*, *Rxrg* or *Crx*; n=6 samples from N=3 differentiation cultures. **, $p<0.01$, Student's *t*-test. **(B)** BrdU pulse proliferation analysis of cultures treated with RA at d14-16. 10 µM BrdU was added to culture media for 1 hr before harvest. Sections immunostained for BrdU in left panels. Scale bar 20 µm. Right panel shows quantification of of BrdU+ nuclei, n>40, N=3, bars indicate mean ±SD, $p<0.05$, unpaired Student's *t*-test. **(C)** Cell viability in dissociated RA-treated cultures. N=3, 36 organoids each sample, mean ±SEM. **(D,E)** Live cell yields determined by viability assay and flow cytometry. (D) Total cell yield. (E) Crx-GFP+ cell yield. N=3, 36 organoids each sample, mean ±SEM. **(F)** RT-PCR analysis of differentiation cultures at post-natal development equivalents d22 (~P2) to d33 (~P13) for cone-specific genes. Negative controls are no template reactions, P8 eye RNA used for positive controls. **(G,H)** Immunostaining for rod (Rhodopsin in red) and cone-specific (Arrestin3 in C and S opsin in D, in green) proteins at d37 of differentiation. Scale bar 10 µm.

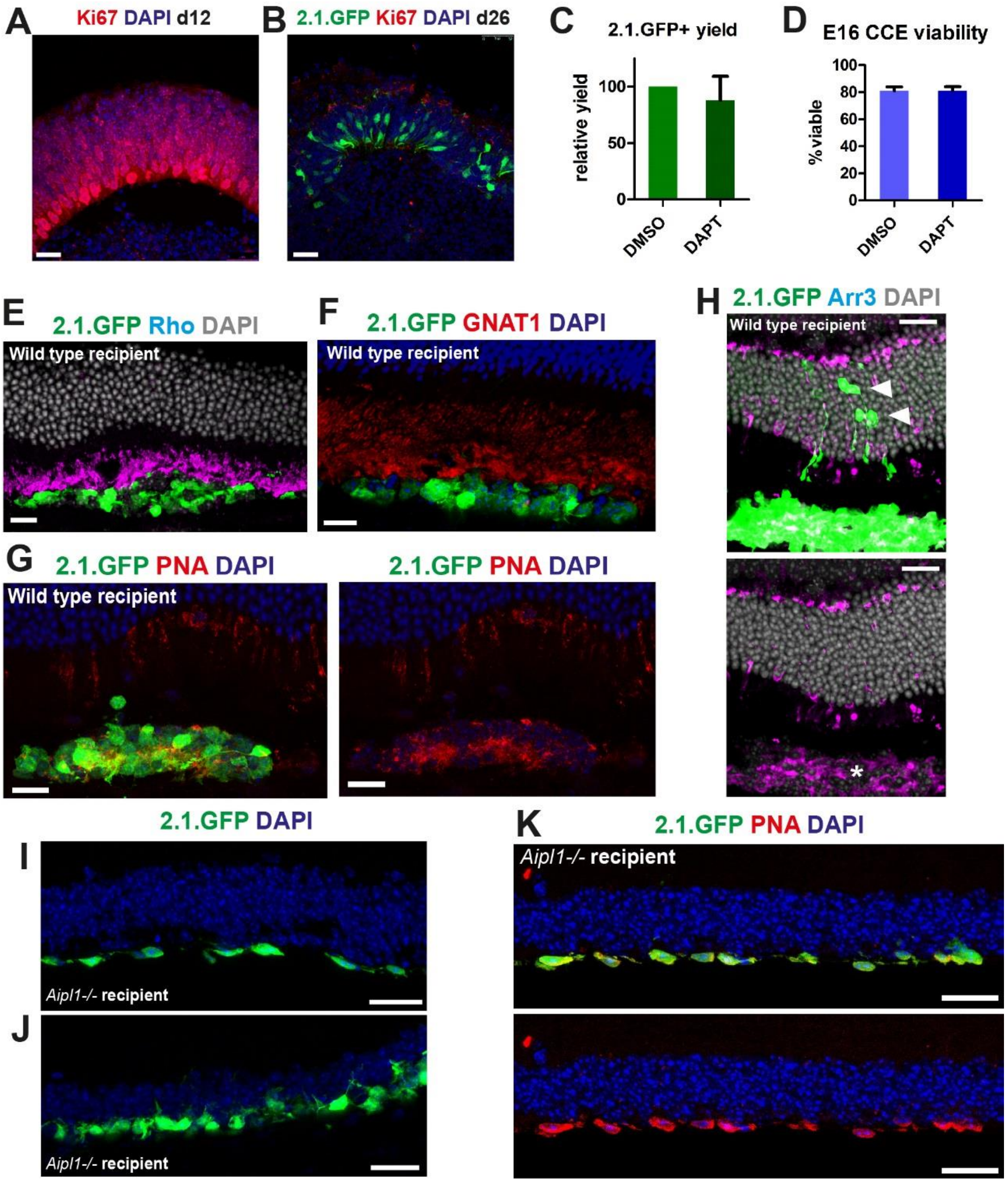

Figure S6. MESC-derived cone precursor transplantation. Related to Figures 6 and 7.

(A,B) Ki67 staining in d12 (A) and 2.1.GFP-transduced d26 organoids. Note loss of Ki67 by d26 in culture. Scale bar 20 µm. **(C)** Cell viability analysis in cultures dissociated according to the protocol used for transplantation. N=3, 36 organoids each sample, 36 organoids each sample, mean ±SEM. **(D)** Live cell yield normalised to DMSO control sample for DAPT-treated cultures in (C) determined by flow cytometry. **(E-H)** Immunostaining for rod, (Rhodopsin and Gnat1, E and F, respectively) and cone (PNA and Arrestin3, G and H, respectively) markers in wildtype host eyes transplanted with 2.1.GFP-expressing cone precursors at 3 weeks post-transplantation. Note the absence of rod-specific proteins Rhodopsin (E, in cyan) and Gnat1 (F, in red) and the presence of cone-specific markers PNA (G, in red) and Arrestin3 (H, in cyan, * indicates the cell mass) in the GFP+ subretinal cell mass. Arrows in H point to GFP+ cells with rod morphology in the host outer nuclear layer that most likely are a result of cytoplasmic material transfer from the cell graft. Scale bars in all images 20 µm. **(I,J)** Morphologies of transplanted cone precursors in the *Aipl1*$^{-/-}$ recipient retina. (I) dispersed cells aligned with neurites extending horizontally along the interneuron layer. (J) In higher density cells showed more extensive neurite outgrowth and polarization. **(K)** Transplanted cells secrete PNA-staining extracellular matrix *in vivo*. Scale bars in all images 25 µm. Samples collected 3 weeks post-transplantation.

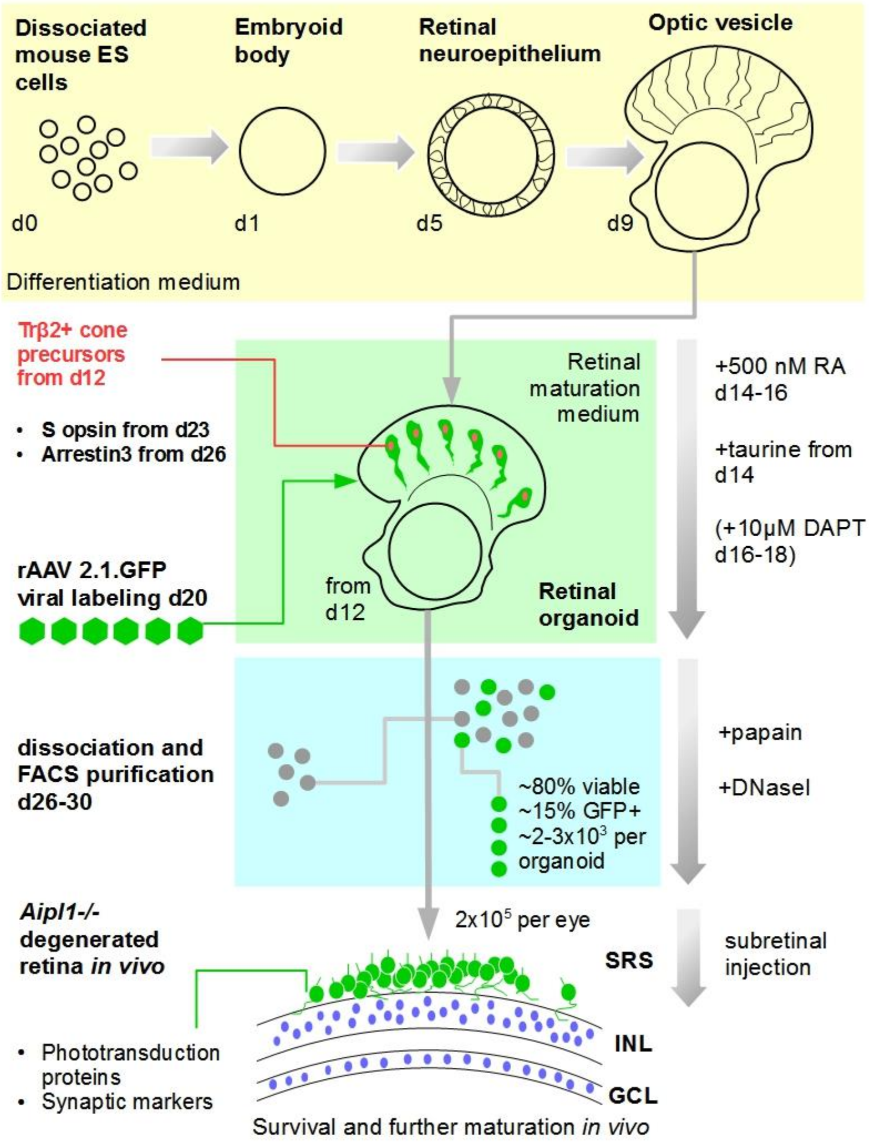

Figure S7. Summary of the protocol for differentiation, isolation and transplantation of mESC-derived cone precursors. Related to all main figures.

Maintenance cultures of mESCs were dissociated and $3x10^3$ cells were plated per well of a 96-well plate to form an embryoid body. Matrigel basement membrane matrix was added next day to stimulate formation of rigid continuous neuroepithelia. Cells were further cultured in retinal differentiation medium (*Differentiation medium*). Retinal neuroepithelia formed around d5 and optic vesicles developed by d9. From d12 the organoids were transferred into retinal maturation medium (*RMM*). At d12 the first cone precursors were detected by immunolabelling for Trβ2. From d14 to 16 a short pulse supplementation of 500 nM RA was performed. Taurine was added from day d14 onwards. Pharmacological inhibition of Notch signalling using 10 µM DAPT at d16 increased the proportion of differentiating cone precursors, without raising the absolute cone precursor cell yield. From d23 S opsin became expressed, whereas Arrestin3 was detected from d26. Labelling of cone precursors for transplantation was performed by transduction with rAAV vector encoding a 2.1 kb human red-green cone opsin promoter fragment driving GFP at d20. For transplantation, performed at d26-d30, organoids were dissociated using a papain-based kit with addition of DNaseI. GFP+ cells were purified from the cell suspension by FACS. Usual cell viability following dissociation was around 80%. Around 15% of cells were GFP+ and $2\text{-}3x10^3$ cone precursors were recovered from a single organoid. $2x10^5$ live cells (approximately equivalent to the number of cones in a wildtype mouse retina) were transplanted via subretinal injection into eyes of severely degenerate *Aipl1*$^{-/-}$ recipients from 8 weeks of age. Histological analysis 3 weeks post-transplantation showed expression of phototransduction-related and synaptic proteins suggesting a degree of maturation *in vivo*.

Supplemental Tables.

Table S1.

List of constructs used in the study.

Plasmid name	***Promoter/s***	***Gene/s of interest***	***Used for***
AAV2/9 cap	ITR	AAV2/9 rep cap	AAV vector production
ShH10 cap	ITR	ShH10 rep cap	AAV vector production
PHGTI	P5	E4, E2a, VA	AAV vector production
pD10.2.1.GFP	2.1*p*	GFP	AAV vector production
pD10.U6.shControl.CBA.RFP	U6, CBA	shControl, RFP	AAV vector production
pD10.U6.shNrl.CBA.RFP	U6, CBA	shNrl, RFP	AAV vector production
pD10.U6.shNr2e3.CBA.RFP	U6, CBA	shNr2e3, RFP	AAV vector production
pD10.Rhop.RFP	Rho*p*	RFP	AAV vector production

Table S2.

Gene-specific PCR primers.

Gene-specific primer details

**Gene name**	Forward Primer (5'-3')	Reverse Primer (5'-3')	Amplicon size (bp)	Probe number
Actb	aaggccaaccgtgaaaagat	gtggtacgaccagaggcatac	100	56
Arr3	tgtgtttgttcaggagttcaca	aggccctgcttctgacagt	105	71
Cnga3	agaacagaagccaccagacg	cagtcagccagcggtagtaga	92	67
Cngb3	tggacaaagcatatgtctacagagt	cccagtaataaacacaggcgtta	87	02
Crx	ccccaatgtggacctgat	ggctcctggtgaatgtggt	89	64
Cyp26a1	ccggcttcaggctacaga	ggagctctgttgacgattgtt	125	17
Hes1	gccagctgatataatggagaaaa	ctccatgataggctttgatgact	127	83
Hes5	ccaaggagaaaaaccgactg	cttggagttgggctggtg	128	67
Gnat1	agagctggagaagaagctgaaa	tagtgctcttcccggattca	95	89
Nrl	ttctggttctgacagtgactacg	tgggactgagcagagagagg	77	53
Nr2e3	cagccagcctgtgaggtt	agaagctcaatgcgctcag	81	32
Oc1	ggagttccagcgcatgtc	cgacgttggacgtctgtg	123	64
Olig2	agaccgagccaacaccag	aagctctcgaatgatccttcttt	107	21
Opn1sw	ccccatcatctactgcttcat	gacacgtcagattcgtctgc	93	04
Opn1mw	atcgtgctctgctacctcca	tttctgttgctttgccactg	60	05
Pde6c	tgactgcctgtgacctgtct	ttgcaaccagaagtgctacct	72	09
Raldh1	caccatggatgcttcagaga	actttcccaccattgagtgc	108	40
Raldh3	tttggtggcttcaaaatgtct	acttctgtatattcagccagagca	71	53
Rara	ggagcttggaacctgcac	gaggatgccactcccaga	76	83
Rcvrn	caatgggaccatcagcaaa	cctcaggcttgatcattttga	71	67
Rho	acctggatcatggcgttg	tgccctcagggatgtacc	70	32
Rorb	gctgactgaggaagagattgc	atcagccaggctcggtct	69	82
Rxrg	cagaagtgcctggtcatgg	cctcactctctgctcgctct	82	82
Thrb2	atgcatctatgttggcatgg	gcttggctagcctcttgct	62	42

Table S3.

Details of antibodies used for immunostaining.

Antigen	Host species	Dilution	Supplier
**Cone arrestin**	rabbit	1 in 250-500	Millipore (AB15282)
**Crx**	rabbit	1 in 500	Gift from C.Gregory-Evans
**GFP**	rabbit	1 in 300	Life Technologies (A-21311)
**GFP**	goat	1 in 300	(FITC-conj.) Abcam ab6662
**Gnat2 (Gαt2)**	rabbit	1 in 500	Santa Cruz (I-20; sc-390)
**Ki67**	rabbit	1 in 100	Abcam (ab15580)
**M opsin**	rabbit	1 in 500	Millipore (AB5405)
**LaminB**	goat	1 in 500	Santa Cruz (C-20; sc-6216)
**Olig2**	rabbit	1 in 100	Millipore (AB9610)
**Onecut1**	rabbit	1 in 100	Santa Cruz (H-100; sc-13050)
**Otx1/2**	rabbit	1 in 200	Abcam (ab21990)
**Peripherin2**	rabbit	1 in 500	Gift from G.Evans
**RALDH1 (ALDH1A1)**	rabbit	1 in 200	Abcam (ab23375)
**RARα**	rabbit	1 in 100	Santa Cruz (C-20; sc-551)
**Recoverin**	rabbit	1 in 500	Chemicon (AB5585)
**Rhodopsin**	mouse	1 in 1000	Sigma (O4886)
**Ribeye (CtBP2)**	mouse	1 in 500	BD Biosciences (612044)
**RXRγ**	rabbit	1 in 100	Santa Cruz (Y-20; sc-555)
**RXRγ**	rabbit	1 in 100	Abcam (ab15518)
**S opsin**	goat	1 in 100	Santa Cruz (H-17; sc-14365)
**S opsin**	rabbit	1 in 250-500	Millipore (AB5407)
**Synaptophysin**	mouse	1 in 1000	Sigma-Aldrich (S5768)
**Trβ2**	rabbit	1 in 500	Wako (016-24261)

Table S4.

Retinal differentiation media.

Maintenance medium	Retinal differentiation medium	Retinal maturation medium
510 ml GMEM<br>5.1 ml Non-essential amino acids<br>5.1 ml Pyruvate (1 mM)<br>0.51 ml 2-Mercaptoethanol<br>58 ml KSR (10%)<br>5.8 ml Heat-inactivated FBS (1%)	510 mls GMEM (Gibco, 21710-082)<br>5.1 mls Non-essential amino acids (Gibco, 11140-035) (0.1mM)<br>5.1 mls Pyruvate (Sigma, S8636) (1mM)<br>0.51 mls 2-ME working solution (0.1mM)<br>8.4 mls KSR (1.5%)	500 ml DMEM/F-12 GlutaMAX<br>5 ml N2 supplement<br>2.5 ml Penicillin/Streptomycin

Supplemental Experimental Procedures

DNA constructs

DNA constructs used are listed in Supplemental Table 1.

2.1.GFP promoter construct was courtesy of J. Nathans. Bovine Rhodopsin promoter (*Rhop.RFP*) was cloned and characterized in the laboratory. Knockdown constructs targeting mouse Nrl and Nr2e3 genes with an RFP reporter were cloned and amplified as described in the following section. Plasmid encoding ShH10 capsid was kindly provided by J. Flannery.

Nrl and *Nr2e3* knockdown constructs

Artificial miRNA-like hairpins targeting the coding sequence of mouse *Nrl* and *Nr2e3* genes were designed using siDESIGN software (Dharmacon). Candidates with highest scores and lowest predicted off-target effects were synthesised. Oligonucleotides coding the miRNA cassette were ligated into U6 plasmid designed for miRNA expression. Positive colonies after transformation were checked by restriction digestion (with *Asc*I and *Sna*BI enzymes) and sequencing. Knockdown efficiency was evaluated in 293T cells by qPCR.

Nrl knockdown target sequences and the short hairpin sequences synthesised were as follows:

sh-Nrl target sequence

5'-GATTTGATGAAGTTCGAAA-3'

short hairpin cassette oligonucleotides sequences:

5'-TTTGGCGAGATTTGATGAAGTTCGAAACTGTGAAGCCACAGATGGGTTTCGAACTTCATCAAATC CTGCTTTT-3'

and

5'-CTAGAAAAGCAGGATTTGATGAAGTTCGAAACCCATCTGTGGCTTCACAGTTTCGAACTTCATCA AATCTCGC -3'

Target sequences and miRNA cassette sequences that showed substantial reduction of *Nr2e3* levels are as follows:

sh-Nr2e3 target sequence

5'-GGACAGCAGCAGTGGGAAA-3',

short hairpin cassette oligonucleotides sequences:

5'-TTTGGCGAGGACAGCAGCAGTGGGAAACTGTGAAGCCACAGATGGGTTTCCCACTGCTGCTGT CCCTGCTTTT-3'

and

5'–CTAGAAAAGCAGGGACAGCAGCAGTGGGAAACCCATCTGTGGCTTCACAGTTTCCCACTGCTG CTGTCCTCGC-3'

sequences forming the hairpin regions are underlined.

Recombinant adeno-associated viral (rAAV) vector production

Production of recombinant AAV vectors was carried out in HEK 293T cell line transfected with three plasmids encoding construct of interest, viral capsid and helper genes (on pHGTI plasmid). The plasmids were transfected using polyethyleneimine (PEI). HEK 293T cells were seeded onto 150 mm tissue culture plates (Greiner Bio-One Ltd., UK) at 10^6 cells per plate in DMEM Glutamax® (Life Technologies Ltd., UK) medium containing 10% foetal bovine serum (Life Technologies Ltd., UK) and penicillin, streptomycin and antifungal agent mix (Life

Technologies Ltd., UK). The following day cells reached approximately 70% confluency. 20 plates of cells were used for a batch of virus. Transfection mixture contained 52.5 ml DMEM, 100 µg capsid plasmid, 30 µg pHGTI helper, 100 µg gene transfer plasmid and 1.2 ml PEI. Transfection solution was incubated at room temperature for 10 minutes and then added dropwise onto plates with cells. Medium was replaced following day. 72 hr from transfection, cells were harvested by scraping off plates using a cell scraper (Greiner Bio-One Ltd., UK). Medium with cells was collected in 50 ml Falcon tubes and spun down at 2000 xg for 5 minutes and resuspended in TD buffer pH 7.4. Release of viral particles from harvested cells and adequate lysis was facilitated by 3 cycles of freezing (in -80°C), thawing (at 37°C) and mixing on a vortex shaker for 5 minutes. This was followed by treating the cell lysate with 50 units of benzonase (Sigma-Aldrich Ltd., UK). Benzonase was used to remove remaining free plasmid DNA. Cell lysate was subsequently centrifuged at 18000 xg and supernatant filtered through membrane filters, first 5 µm pores, then 0.45 µm and, finally, 0.22 µm. Viral vector was purified from this supernatant by means of ion exchange fast protein liquid chromatography (FPLC). ÄKTA™ prime or ÄKTA™ pure (GE Healthcare Ltd., UK) FPLC apparatus was used to purify vector particles on anionic sephacryl S300 and a POROS 50HQ columns prior to elution using increasing salt gradient. This yielded approximately 20 mls of eluate which was concentrated on Vivaspin 4 columns (Sartorius AG., Germany) to a final volume of 250 µl. Viral preparation was aliquoted and stored at -80°C until use. Concentration of copies of viral genome in ml of vector preparation was determined by quantitative real time PCR using primers specific for viral inverted terminal repeat (ITR). Number of copies of viral genome was determined by comparison of amplification of samples with amplification of standards of known quantity of vector.

Quantitative PCR

50 ng of cDNA was loaded per well of 96-well plate (Life Technologies Ltd., UK) mixed with 2x Fast Start TaqMan® Probe Master Mix (Roche Ltd., UK), gene-specific forward and reverse primers at a final 900 nM concentration and an appropriate hydrolysis probe binding to the amplified region at a final concentration of 250 nM (Roche Diagnostics Ltd., UK), all dissolved in DNase and RNase free water up to 20 µl final volume. Each cDNA samples was run in triplicate. The reactions were then run on an ABI Prism 7900HT Fast Real-time Sequence Detection System (Applied Biosystems Ltd., UK) equipped with SDS 2.2.2 software for amplication results analysis. From amplification curves Ct values were obtained for each sample. Expression levels were normalized to beta actin (*Actb* gene) mRNA levels for each sample to assess relative expression of particular genes in different experimental conditions. Cycling conditions were as follows 40 cycles of 95°C for 30 sec. and 60°C for 1 minute. Supplementary Table 2 contains the list of gene-specific primer sequences used.

Immunohistochemistry

Tissue from was frozen in OCT embedding matrix (Pyramid Innovation Ltd., UK) and sectioned at 18 µm on a Bright OTF 5000 cryostat (Bright Instruments Ltd., UK). Cryosections were thawed and rehydrated by adding PBS. For certain antibody staining sections were treated with 1 or 4% paraformaldehyde solution for 10 minutes followed by a wash with PBS. Slides were blocked with a solution of 5% goat or donkey serum, 1% BSA, 0.1% Triton X-100 in PBS for 2 hours at room temperature. This was followed by 5 washes with PBS, after which primary antibodies were added dissolved in 1% BSA, 0.1% Triton X-100 in PBS and incubated overnight at 4°C. List of primary antibodies used in this study can be found in Table 3. Following day slides were washed 5 times with PBS, before adding Alexa Fluor®-conjugated secondary antibodies (Life Technologies Ltd., UK) diluted 1:100-1:500 (depending on the primary antibody used) in 1% BSA, 0.1% Triton X-100 in PBS. Slides were incubated with secondary antibodies for 2 hours at room temperature. Subsequently, they were washed 3 times with PBS and nuclei counterstained with 4'-6-diamidino-2-phenylindole (DAPI; Sigma-Aldrich Ltd., UK) and mounted using coverslides and fluorescent microscopy mounting medium (DAKO Ltd., UK).

All antibodies used are listed in Supplemental Table 3.

Cell counts

Quantification of the percentage of cells expressing cone markers in retinal organoids was performed using 10 µm thick confocal stacks (acquired under 40x objective) using Cell Counter plug-in in ImageJ software. Numbers of marker-positive cells were normalized either to DAPI-stained nuclei or CrxGFP+ cells in the same images. Multiple individual organoids from at least 3 differentiation cultures were used for each quantification as specified in respective figure descriptions.

CrxGFP+/S opsin+ cells were counted on 18 µm-thick P8 CrxGFP transgenic mouse retina cryosections. Using a 40x objective, 10 µm-thick confocal Z stacks were acquired from dorsal, mid-central and ventral retina, 9 images per retina, n=3 retinae from 3 individual mice. Results were averaged for each retina analysed.

Rxrγ-positive nuclei and M, S opsin-positive photoreceptor processes were counted in 15 µm-thick confocal stacks (40x objective) and normalized to CrxGFP+ photoreceptors present in the image. A minimum of 3 images from each transplanted retinae were used, with 3 transplanted retinae examined.

Mouse ESC maintenance culture

Maintenance of undifferentiated mouse ES cells state was performed by culture in the presence of leukemia inhibitory factor (LIF, Millipore Ltd., UK) or, alternatively the '2i' medium containing CHIR99021 and PD03259010, inhibitors of GSK3β and MEK respectively. LIF was used at 1000U/ml, whilst CHIR99021 at 3 µM and PD03259010 at 1 µM. Cells were kept as adherent cultures on 0.1% gelatin-coated dishes. To avoid spontaneous differentiation, the cells were kept at low (below 30%) confluence and passaged every other day. Cells were dissociated for 5 minutes at 37°C using 0.25% trypsin-EDTA solution (Life Technologies Ltd., UK). Viability was assessed using trypan blue dye. Concentration of cells was determined with a haemocytometer and 1.5 x 10^5 cells were added per 60 mm plate, for the larger 100 mm plate 4.2 x 10^5 cells were plated in maintenance medium containing 1000U/ml of LIF. Cells were cultured at 37°C in 5% CO_2.

Retinal differentiation

Day 0. Maintenance cultures were dissociated using 0.25% trypsin-EDTA solution. Cells were subsequently plated into Nunclon Sphera ultra low-binding 96-well plates (Thermo-Fisher Scientific Ltd, UK). Total of 3.0 x 10^5 cells was resuspended in 10 ml of differentiation medium, mixed through pipetting and 100 µl of suspension added per well of the 96-well plate, so that an average of 3000 cells was plated into each well subsequently aggregating to form an embryoid body.

Day 1. 24 hours after plating the aggregates were examined under a light microscope to assess embryoid body formation followed by addition of matrigel solution to trigger continuous retinal neuroepithelia formation. Matrigel solution was prepared by rapidly dissolving 1 ml of growth factor reduced (GFR) Matrigel working solution (1:35 dilution) in 4 ml of differentiation medium per 96-well plate. Next, 50 µl of this freshly prepared Matrigel solution was added per well to give a final concentration of 2% Matrigel. Following Matrigel addition aggregates were cultured for another 8 days at 37°C in 5% CO_2.

Day 9. Organoids were transferred into a low-binding 24-well tissue culture plate (Thermo-Fisher Scientific Ltd, UK) using wide bore pipette tips (VWR Ltd, UK), 12 aggregates per single well of a 24-well plate. Differentiation medium carried over with the embryoid bodies was gently aspirated using a 1 ml pipette and replaced with 1 ml of pre-warmed retinal maturation medium (RMM).

Day 14. 5 µl of retinoic acid working solution was added per ml of RMM (500 nM final concentration) and 3 µl per ml of taurine working solution (150 nM final concentration).

Media components are listed in Supplemental Table 4.

Treatment with with Notch inhibitors DAPT and RO4929097

Organoids at either day 16 or 21 of culture had their media replaced with pre-warmed RMM supplemented with 150 nM taurine and containing DMSO as a vehicle control or 10 µM final concentration of DAPT (Sigma-Aldrich Ltd, UK) or RO4929097 (BioVision Inc., USA; 1 µl of 10 mM working solution and 10 µl of 1 mM working solution, respectively). Cells were next incubated with inhibitors for 48 hours before processing for analysis by flow cytometry or collection for quantitative PCR.

Dissociation of retinal organoids

Papain-based Miltenyi Biotec Neural Tissue Dissociation Kit (Miltenyi Biotec GmbH. Germany) was used according to manufacturer's recommendations. Briefly, solution 2 from the kit was pre-warmed for 15 minutes in a water bath set at 37°C, 960 µl was used per 70-100 aggregates. Embryoid bodies were collected with wide-bore pipette tips into 15 ml Falcon tubes (aggregates from 6-8 wells into a single tube). Embryoid bodies were left for several minutes to sink down to the conical bottom of the tube. RMM medium was removed from above the aggregates and they were washed with 10 ml of PBS (without Ca^{2+} and Mg^{2+} ions; Thermo-Fisher Scientific Ltd, UK). PBS was aspirated and organoids suspended in pre-heated solution 2. To each tube 25 µl of solution 1, 10

µl of solution 3 and 5 µl of solution of 4 was added and mixed with the organoids in suspension. The falcon tubes were then incubated at 37°C in a water bath for 15-25 minutes, mixed every 3-5 minutes. Following the incubation, organoids were mechanically dissociated by gentle trituration with a P1000 pipette. Partially dissociated samples were then incubated at 37°C for another 5 minutes and again triturated. Tubes were then centrifuged at 1000 rpm for 7 minutes. Supernatant was aspirated and cells resuspended in 1 ml of resuspension solution composed of 66% Eagle's Minimum Essential Medium (MEM-E HEPES), 33% Hank's Balanced Salt Solution (HBSS), 1% heat-inactivated FBS (all Thermo-Fisher Scientific Ltd, UK) and supplemented with 20 units/ml of DNase I (Sigma-Aldrich Ltd, UK). Once homogenous, the solution was passed through a 40µm cell strainer (BD Biosciences Ltd, UK).

Fluorescence-activated cell sorting

Cell sorting was performed either using BD Influx Cell Sorter (Beckman Dickinson Inc., USA) or MoFlo XDP (Beckman Coulter Inc., USA). Both sorters were fitted with a 200 mW 488 nm blue laser (adjusted to 150 mW for sorting) that was used to excite GFP or RFP with the GFP signal detected in 530/40 nm channel, whilst RFP signal in 613/20 nm channel. Sorting of photoreceptor cells was performed at 50 psi and a 70 nozzle was used. Flow-sorted mESC-derived GFP+ cells were on average >95% pure GFP-positive, and >80% viable.

Flow cytometry analysis

DRAQ7 (Biostatus, DR71000) dead cell stain was added to the samples at a final concentration of 0.3µM for 5 minutes at room temperature, followed by sample acquisition. All of the samples were analysed using a BD LSRFortessa X-20 flow cytometer (Beckman Dickinson Inc., USA), fitted with 5 lasers (i.e. 355nm, 405nm, 488nnm, 561nm & 640nm lasers). Results were subsequently analysed using FlowJo software.

Viability and cell yields assessment

Cell viability and number in dissociated samples was determined using Vi-Cell^{TM} XR cell viability analyser. Live/dead discrimination was performed using trypan blue dye. Cell yields were calculated by combining cell number and viability with flow cytometry results. In each experiment the results were normalised to either untreated or DMSO control conditions (given value of 100) to assess treatment effect. Three independent experiments with at least 36 organoids each were performed.

Animal models

Care of all animals used in this study was according to standards described in the Animal (Scientific Procedures) Act 1986. All the procedures were performed in accordance with the Association for Research in Vision and Ophthalmology (ARVO) Statement on the Use of Animals in Ophthalmic and Vision Research. Transplantation of photoreceptor precursors was performed at 8-16 weeks of age in both wild type and *Aipl1*$^{-/-}$ animals. Animals used were housed in conditions of standard 12 hour light-darkness cycle with food and water *ad libitum*.

Anaesthesia

Animals were anaesthetised by intraperitoneal injections of an anaesthetic solution composed of Dormitor (1 mg/ml, Pfizer Pharmaceuticals Ltd, UK) and ketamine (100 mg/ml, Fort Dodge Animals Health Ltd, UK) mixed with sterile water for injections (Thermo-Fisher Scientific Ltd, UK) in a ratio of 5:3:42. Young adult animals that were used for subretinal injections weighing approximately 200g received 0.2 ml of anaesthetic solution. Once anaesthetised, a small drop of Viscotears (Alcon Ltd, UK) was used topically on the cornea to prevent it from drying during the procedure. After the injections, in order to reverse the effects of the anaesthetic, 0.2 ml of Antisedan (0.1 mg/ml, Pfizer Pharmaceuticals Ltd, UK) was administered through intraperitoneal injection and the mice were placed on a heat mat until they regained normal mobility.

Subretinal transplantation

Cells sorted into 15 ml Falcon tubes were centrifuged for 10 minutes at 1000 rpm and resuspended in HBSS (+Ca^{2+}, +Mg^{2+}) supplemented with DNase I (50 units/ml). Viability was determined using trypan blue staining, cells were counted using Neubauer haemocytometer and resuspended to a final of 10^5 cells per µl. Before surgery, pupils of the animals were dilated by topical administration of 1% Tropicamide solution (Chauvin Pharmaceuticals Ltd, UK). Surgery was performed under direct retinoscopy utilising an operating microscope (Leica AG, Germany). In preparation for injections, the eye was protruded forward by application of small

pressure on both sides of the eye and kept in place by holding a section of conjunctiva and extraocular muscle using a pair of forceps. By placing a coverslip on the cornea covered in Viscotears solution (Alcon Ltd, UK) acting as coupling medium, a contact lens system was created enabling visualisation of the fundus. Injections were performed with a 1.5 cm, 34-gauge hypodermic needle mounted on a 5 µl Hamilton syringe (Thermo-Fisher Scientific Ltd, UK). Tip of the syringe was placed underneath the coverslip and then guided to the sclera and then inserted tangentially through it creating a wound tunnel that self-seals. Once the needle was inserted its tip was brought into focus between the retina and the RPE and 1 µl of cell suspension containing 10^5 cells selected for GFP expression was injected. This procedure was performed for the superior and inferior hemispheres of the eye.
